# Base-Promoted Iridium-Catalyzed Deuteration and C–H Bond Activation of *N*‑Heterocycles

**DOI:** 10.1021/acs.joc.5c00174

**Published:** 2025-06-05

**Authors:** Ben J. Tickner, Claire Condon, Victoria Annis, Richard J. Gammons, Adrian C. Whitwood, Simon B. Duckett

**Affiliations:** † Centre for Hyperpolarisation in Magnetic Resonance, 8748 University of York, Heslington YO10 5NY, U.K.; ‡ Department of Chemistry, 8748 University of York, Heslington YO10 5DD, U.K.

## Abstract

Hydrogen isotope exchange (HIE) of *N*-heterocycles is highly important in synthesis, where it is often used to prepare probes suitable for pharmaceutical studies. In this work, we show that pharmaceuticals such as anastrozole, trimethoprim, and bisacodyl can be easily deuterated in up to 84–95%, with high site selectivity using an [IrCl­(COD)­(IMes)]/H_2_/NaOMe/methanol-*d*_4_ derived catalytic system. We studied in detail the deuteration of quinoxaline using NMR spectroscopy, mass spectrometry, and X-ray crystallography and characterized a range of C–H bond activated products that include [Ir­(H)_2_(quinoxaline)­(IMes)­(κ^2^-μ_2_-C,N-quinoaxline)_2_Ir­(H)_2_(quinoxaline)­(IMes)]; [Ir­(H)_2_(quinoxaline)­(IMes)­(κ^2^-μ_2_-C,N-quinoxaline)_2_Ir­(Cl)­(H)­(quinoxaline)­(IMes)]; [Ir­(H)_2_(IMes)­(κ^2^-μ_2_-C,N-quinoxaline)_2_Ir­(H)_2_(IMes)]; and [Ir­(H)_2_(IMes)­(κ^2^-μ_2_-C,N-quinoxaline)_2_(μ_2_–H)­Ir­(H)­(quinoxaline)­(IMes)]. Reaction scope is demonstrated by the examination of 17 structurally diverse substrates, with functional groups spanning pyridines, quinoxalines, quinolines, purine/xanthines, triazoles, and pyrimidines. Four analogous C–H-bond-activated X-ray structures were obtained for the additional substrates. Catalytic turnover was found to dramatically increase upon the addition of NaOMe and is linked to a demonstrated role for trihydride species [Ir­(H)_3_(COD)­(IMes)] in the HIE process, with the reactive fragment {IrH­(IMes)} implicated in the formation of catalytically competent dinuclear C–H bond activation products.

## Graphical abstract

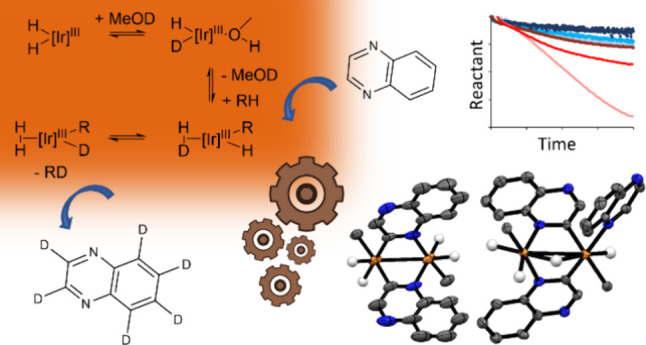

## Introduction

The deuterium labeling of drugs and other agents has become a key area in biomedicine and the pharmaceutical sector.^1–4^ Furthermore, deuterium-labeled probes are widely used in mechanistic and biochemical studies, as the fate of a particular molecular site can be readily tracked by following the deuterium label. This approach has proven particularly useful in elucidating the reaction pathways of many organic transformations and for monitoring metabolism using deuterium metabolic imaging.^2,4,5^ Therefore, the development of novel reactions that can swap specific proton sites of interest for deuterium is highly sought after.^3,6–11^ This is typically achieved via reactions of a target molecule with a metal catalyst and a feedstock containing deuterium. A common feedstock is D_2_, as it can be introduced to unsaturated groups using well understood hydrogenation and hydroformylation-type reactions.^12,13^ Other sources of deuterium include deuterated solvents, which typically exchange their deuterium label into a target site(s), often catalyzed by a metal center,^14–19^ or facilitated by the addition of a base.^20,21^ Metal catalysts involved in these reactions are typically based on late transition metals, such as Ir, Rh, Ru, and Pd, although period 4 metals such as Mn can also be used.

One such precursor family that is known to form isotope exchange catalysts is based on Ir­(I) square planar complexes of the form [IrX­(COD)­(NHC)], where COD is 1,5-cyclooctadiene and X is a phosphine or halide, although related systems using bidentate Ir complexes have also been used.^22–25^ In this work, we exploit the precursor [IrCl­(COD)­(IMes)], which is noted for its air stability and is already widely used as a precatalyst for hydrogen isotope exchange (HIE),^26–28^ hydrogenation reactions,^29^ and the signal amplification by reversible exchange (SABRE) hyperpolarization method.^29,30^ In the latter processes, the precatalyst [IrCl­(COD)­(IMes)] reacts with H_2_ and a suitable ligating molecule like an *N*-heterocycle (L) to form a [Ir­(H)_2_(IMes)­(L)_3_]Cl or [Ir­(Cl)­(H)_2_(IMes)­(L)_2_] type species. Notably, these species exchange H_2_ and L reversibly, and this facet is exploited to drive hydrogenation, or magnetization transfer from *para*hydrogen (*p*H_2_).^30–33^ If D_2_ is used in place of H_2_, then these catalytic systems can incorporate deuterium into L through a process known as the HIE reaction. Interestingly, D_2_ is not necessarily required as the deuterium feedstock, as HIE can occur when complexes of this type are reacted with H_2_ in methanol-*d*_4_. In this case, solvent adducts such as [Ir­(H)_2_(target)_2_(ODCD_3_)­(IMes)]Cl are proposed to form^34,35^ that serve as catalysts to scramble the Ir–H sites with Ir-D, leading ultimately to deuteration of L.^36,37^ As the metal complex, solvent, L, and H_2_ are all in dynamic exchange, the deuterium label is introduced into the metal hydride positions and, consequently, HD and H_2_ gas can form.^38–43^ Mechanistic studies involving Ir-D species, formed either from oxidative addition of D_2_ to the metal center, or from exchange with a deuterated solvent, have been interpreted to suggest that C–H bond activation within the ligand yields Ir­(H-D)­(H)­(R) or Ir­(D_2_)­(H)­(R)-type reaction intermediates which, can form the required deuterated products in line with a σ bond assisted metathesis process (Figure 1).^25,44–47^

**Figure 1:**
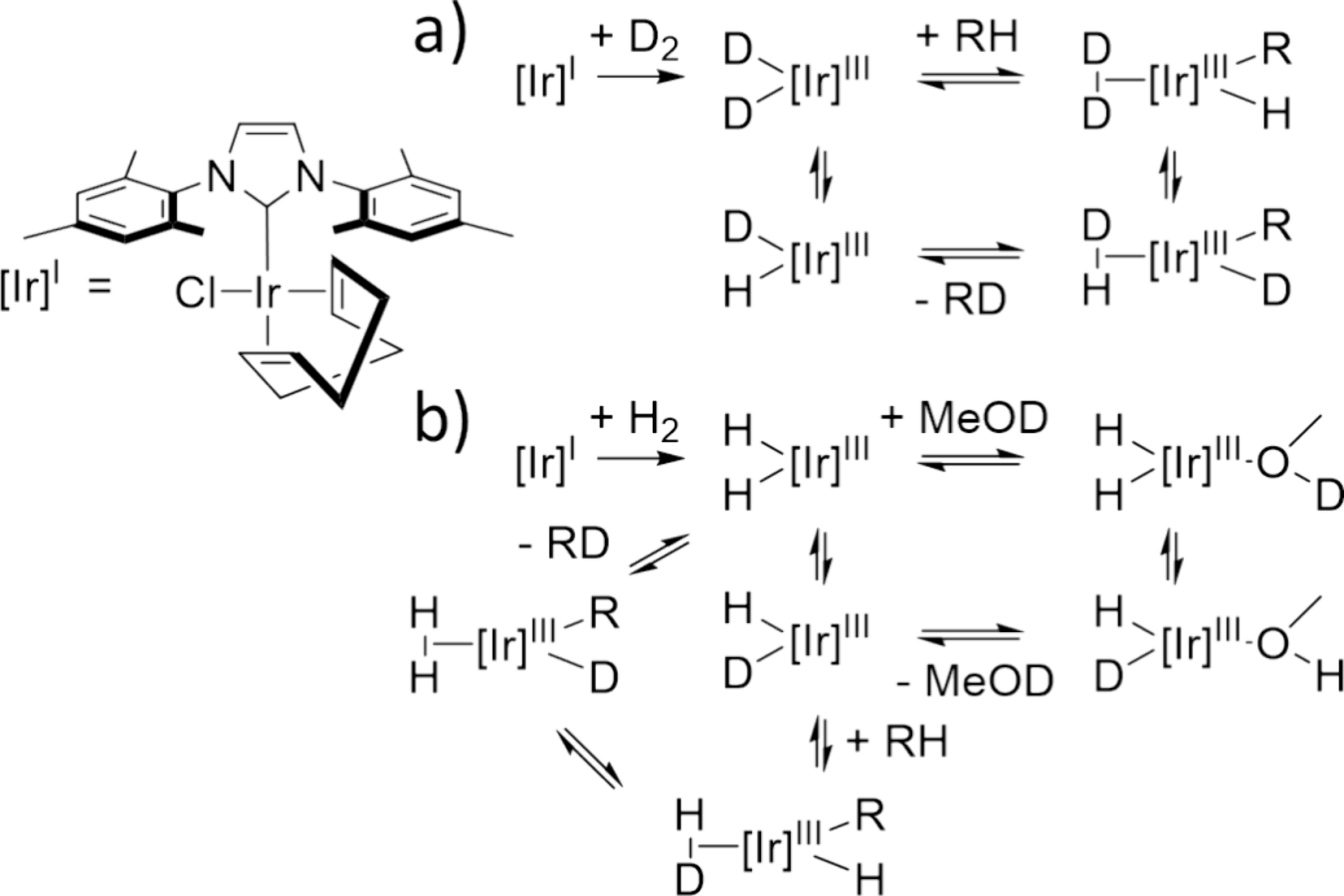
Target molecules (RH) can become deuterated by iridium-catalyzed (a) reaction with D_2_, or (b) reaction with H_2_ in methanol-*d*_4_. In the latter case, methanol-bound adducts serve to introduce a deuterium label to the metal coordination sphere before C–H bond activation occurs, enabling a new C–D bond to form in line with a σ bond-assisted metathesis process that transfers the deuterium label to the target. The target often contains a donor group to help direct the site of CH label exchange. In these cases, the target may act as a bidentate ligand.

In this work, we explore the deuterium exchange that occurs when [IrCl­(COD)­(IMes)] reacts with a target molecule and H_2_ in methanol-*d*_4_. Specifically, we investigate the types of molecular scaffolds that can be deuterated by this catalytic system. While various catalytic systems, based on other transition metals, have been shown to facilitate the deuteration of N-heterocycles,^48,49^ the catalytic system employed here, [IrCl­(COD)­(IMes)], is noted for its air stability. Furthermore, it is already widely used as a precatalyst for HIE,^26–28^ hydrogenation reactions,^29^ and the SABRE hyperpolarization method.^33^ During SABRE, target molecules have been observed to undergo deuteration.^36,37,50^ Building on these observations, we employ similar reaction conditions utilizing a methanol-*d*_4_ feedstock to investigate deuteration reactions that can occur under SABRE-like conditions. Based on our findings, we aim to expand the applicability of this system and demonstrate its utility as a viable synthetic route for the selective and efficient deuteration of a range of N-heterocycles.

Accordingly, we begin by examining the extent and scope of deuteration under conditions typically employed for SABRE ([IrCl­(COD)­(IMes)] (5 mM) and target N-heterocycle (50 mM) under a H_2_ (3 bar) environment with methanol-*d*_4_ (0.6 mL)). Notably, deuteration levels in the products can be dramatically improved by the addition of the base NaOMe. We use a combination of NMR spectroscopy, mass spectrometry, and X-ray crystallography to monitor these reactions and characterize a range of species that play a role in this reactivity. We show that the catalytic pathway changes when a base is added, as metal trihydride species are now readily formed. These catalytic systems deuterate a large range of *N*-heterocyclic scaffolds with high conversion (up to *ca* 90% site deuteration) and good selectivity, as demonstrated for the pharmaceuticals anastrozole and trimethoprim.

## Results and Discussion

### Deuteration of Quinoxaline (A) and 3,5-Dichloropyridine (B)

Our study begins with experiments on the test substrates quinoxaline (**A**) and 3,5-dichloropyridine (**B**). **A** consists of fused benzene and pyrazine rings, and its derivatives feature in a wide range of pharmaceuticals (particularly antibiotics) and dyes. Similarly, the pyridyl scaffold of **B** is found in many drugs and pharmaceuticals.^51^ When **A** or **B** (50 mM) are studied with [IrCl­(COD)­(IMes)] (**1**) (5 mM) (COD, *cis*,*cis*-1,5-cyclooctadiene; IMes, 1,3-bis­(2,4,6-trimethyl-phenyl)­imidazole-2-ylidene) and H_2_ (3 bar) in methanol-*d*_4_ (0.6 mL), all of their proton sites become isotopically labeled with deuterium. This is most readily discerned by the visible decrease in ^1^H NMR signal intensity for the three resonances in **A** at δ 8.92, 8.14, and 7.90, which fall by 21, 18, and 11%, respectively (and arise from H_a_, H_c_, and H_b_ of Figure 2a) after 13 h at 298 K. Similarly, the H_a_ and H_b_ sites of **B**, which provide resonances at δ 8.50 and 7.99, respectively, drop in intensity by 31 and 17% over in the same period (Figure 2b). Interestingly, when these experiments are repeated in the presence of the base NaOMe (50 mM), the signals for the H_a_, H_c_, and H_b_ sites of **A** decrease by 74, 36, and 23%, respectively, over the same time period (Figure 2a), while in the analogous experiment for **B**, the two sites show signal decreases of 77 and 13%, respectively (Figure 2b). This suggests that the rate of iridium-catalyzed deuteration at the *ortho* sites within **A** and **B** is improved by factors of up to 3.5 and 2.5 in the presence of base. Consequently, the turnover numbers of **A** and **B** increase from 2.6 and 3.2, respectively, to 9.3 and 8.0 when NaOMe is added. Turnover frequencies similarly increase from 5.5 × 10^–5^ and 6.7 × 10^–5^ s^–1^ for **A** and **B**, respectively, to 1.9 × 10^–4^ and 1.7 × 10^–4^ s^–1^ when NaOMe is added.^52–54^ For context, the deuteration in **A** and **B** was independently confirmed by mass spectrometry (see the Supporting Information, Section S3). When the reaction times under basic conditions are extended from 13 to 24 h, the extent of deuteration of the H_a_ and H_c_ sites of **A** increases slightly to 79 and 40%, while that of H_b_ remains largely unchanged at 23%. Similarly, the extent of deuteration of **B** can be increased to 79 and 14% for the *ortho* and *para* sites, respectively, when the reaction time is increased to 24 h. For **A** and **B** reacting without base, the amount of deuteration incorporation after 24 h was comparable to that achieved after 13 h. It is worth noting that of all the proton sites in **A** and **B**, the H_b_ site of **B** is the only one that does not display increased deuteration incorporation after 13 or 24 h when NaOMe is included. However, when the reaction time for **B** was extended to a week reaction at room temperature, the deuteration incorporation increased to 85 and 20% for the H_a_ and H_b_ sites, respectively. At this time point, deuteration of the H_b_ site of **B** is now marginally higher with NaOMe than the equivalent reaction without it, which seems to plateau at 17% by 13 h.

**Figure 2:**
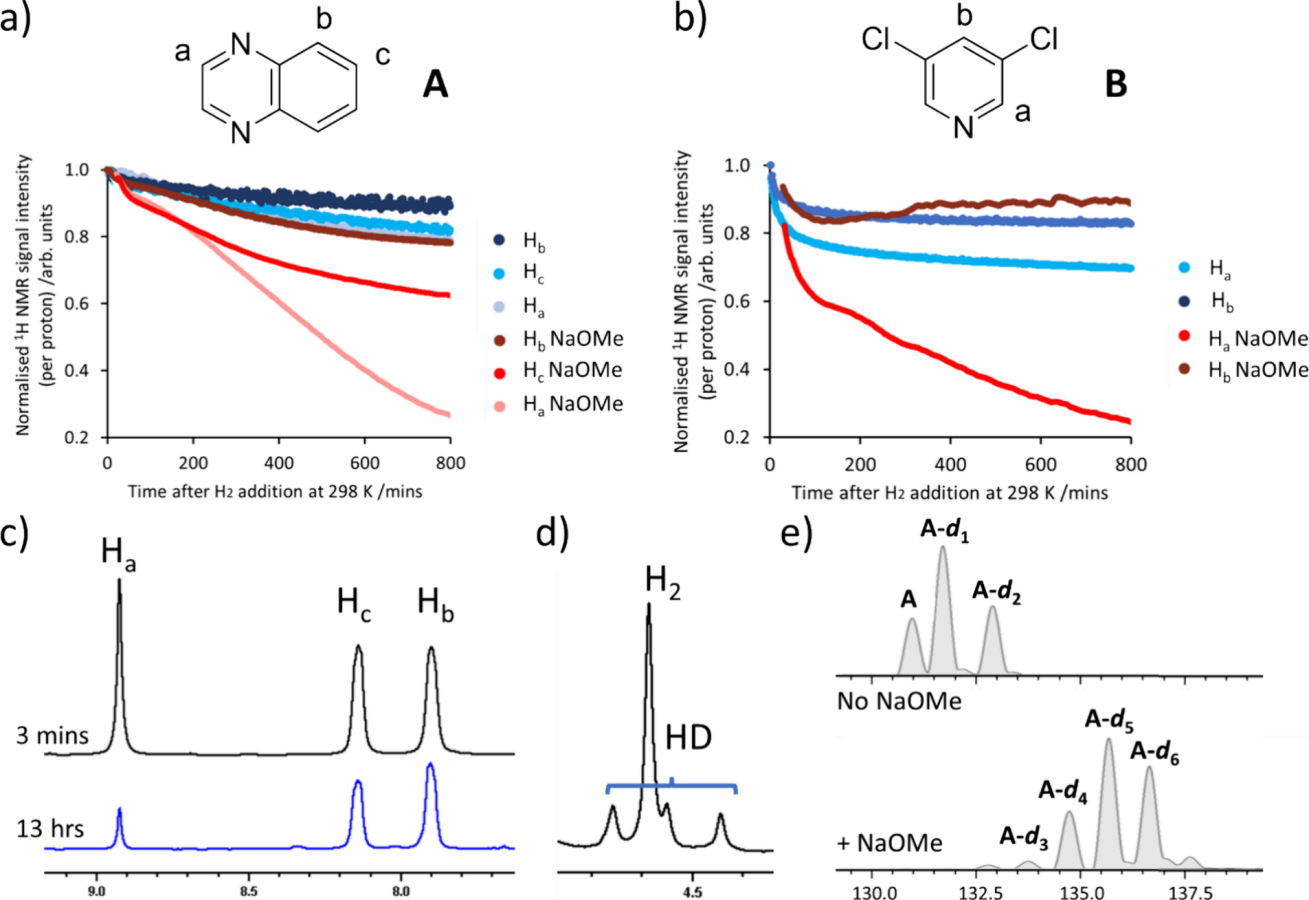
Kinetic time courses, determined from ^1^H NMR spectroscopy at 9.4 T, showing the drop in the ^1^H signal intensity of (a) **A** and (b) **B** (50 mM) after they are reacted with [IrCl­(COD)­(IMes)] (**1**) (5 mM) and H_2_ (3 bar) in methanol-*d*_4_ (0.6 mL) as their proton sites become isotopically labeled with deuterium. For those traces labeled NaOMe, the reaction mixture also contained NaOMe (50 mM). The starting ^1^H NMR signal intensity for each site was normalized to one. (c) Partial ^1^H NMR spectra of the quinoxaline region for solutions containing **A** and NaOMe and 3 min (upper) and 13 h (lower) reaction time used to generate the time courses in a). (d) Partial ^1^H NMR spectra showing the formation of HD for a solution containing **A** and NaOMe 3 min after H_2_ addition. (e) Corresponding mass spectra of these solutions containing **A** after 24 h room temperature deuteration.

### Mechanistic Rationalization of Base-Promoted Deuteration

The experiments detailed so far have confirmed that both **A** and **B** are deuterated relatively slowly in the [IrCl­(COD)­(IMes)]/H_2_/CD_3_OD catalyst system, with and without NaOMe. A series of control experiments were performed to probe the mechanistic pathways operating during these reactions. As deprotonation of the substrate by the base could lead to deuteration, these control experiments started without the iridium catalyst. Accordingly, solutions containing either **A** or **B** (50 mM) in methanol-*d*_4_ (0.6 mL) were examined and their ^1^H NMR signals did not undergo any changes in ^1^H signal intensity or chemical shift in the presence of an excess of NaOMe. Collectively, this suggests that these substrates are not deprotonated by methoxide under these conditions. This is as expected as the p*K*_a_ of the C–H bonds in pyridine-type substrates is reported to be ca. 40,^55,56^ but the p*K*_a_ of methanol (the conjugate acid of methoxide base) is only 15.5,^57^ meaning methoxide is not a strong enough base to deprotonate the substrates of this study on its own. Furthermore, when the precatalyst **1** (5 mM) is introduced into these solutions without any H_2_, no deuteration of **A** or **B** is observed to take place at room temperature over 24 h. Hence, precatalyst **1** is not responsible for their subsequent deuteration.

When the ^1^H NMR experiments described earlier are examined in more detail, it is found that the H_2_ signal at δ 4.59 rapidly became flanked by a signal for HD in a 1:1:1 triplet (^1^ *J*_HD_ = 43 Hz) centered at δ 4.55. Collectively, these points suggest a mechanistic pathway in which the deuterium label of the solvent methanol moves into **A** or **B** and H_2_. Accordingly, the metal species that catalyze this process must drive HIE into the substrate, H_2,_ and methanol. In fact, it takes just 3 min after the reaction of **A** or **B** begins under basic conditions at 298 K for the H_2_ amount in solution to decrease to 10% of its starting value. The initial drop of the free H_2_ signal in solution observed in these data is partly due to the irreversible hydrogenation of the diene ligand of **1**. At this point, of the remaining H_2_, HD now accounts for *ca* 50% of the NMR visible dihydrogen with D_2_ unaccounted for. From this point onward, the overall concentration of dihydrogen (H_2_, HD, and D_2_) should remain constant, but interestingly, over the next 13 h the proportion of H_2_ begins to recover, with the proportion of HD also rising. The associated rise in signal intensity is a consequence of the exchange of the *protio* label of **A** and **B** with D_2_ to form HD and H_2_, which is more prevalent in samples containing NaOMe when the CD_3_OD present has become CD_3_OH. Running the reverse experiment, starting under D_2_, instead of H_2_, in CD_3_OH, instead of CD_3_OD, resulted in the formation of HD and H_2_, thereby confirming that D_2_ would form in the deuteration reactions. Interestingly, in these reverse reactions, the extent of substrate deuteration after 13 h is lower (30, 21, and 22% for the H_a_, H_c_, and H_b_ sites of **A**, respectively, and 51 and 13% for the H_a_ and H_b_ sites of **B**, respectively), which is likely a consequence of the fact that substrate deuteration now competes with deuteration of the much larger pool of solvent protons.

This behavior is markedly different from that observed in the absence of NaOMe, as now the decrease in H_2_ signal intensity takes place much more slowly, taking 15 and 25 min, respectively, for the visible H_2_ concentration to decrease to 10% of its starting value in the associated reactions with **A** and **B**, respectively. In both cases, the increase in H_2_ concentration seen at longer reaction times is much less pronounced and commensurate with the lower proportion of the D-label incorporated into the substrate in these experiments. While repeating these deuteration experiments for **A** and **B** under H_2_ using methanol-*d*_*1*_ as the D-source yielded similar reaction profiles, no deuteration was observed when methanol-*d*_*3*_ is employed. Collectively, these points confirm that the deuterium label that is incorporated into both the substrate and H_2_ originates from the -OD position of the solvent.

### Characterization of Catalytic Species Involved in Base-Promoted Deuteration of A

In order to rationalize the observed difference between the efficiency of deuteration of **A** with and without base, we used NMR spectroscopy to probe for detectable species in solution. The discernible species formed with **A**, without a base, are already relatively well understood. For example, the reaction of **A** with **1** in methanol-*d*_4_ in the presence of H_2_ forms [IrCl­(H)_2_(IMes)­(**A**)_2_] (**2**) as the dominant product, as confirmed by 2D NMR spectroscopy at 245 K (see the Supporting Information, Section S1.1). The presence of further species such as [Ir­(H)_2_(IMes)­(**A**)_2_(methanol)] (**3**) has also been reported for pyridine.^34,35^ Such species have also been detected indirectly using CEST and are likely to play a role in this process, not least because they facilitate H/D exchange between H_2_ and CD_3_OD.

We then directed our attention to precisely probe the solutions of **A**, **1** and H_2_ with added NaOMe in methanol-*d*_4_. To achieve this, a solution of **A** (50 mM) with (**1**) (5 mM), NaOMe (50 mM) in methanol-*d*_4_ (0.6 mL) was reacted with H_2_ (3 bar) for 30 min at room temperature before being cooled to 245 K to probe the metal hydride species present. These NMR spectra revealed the presence of hydride ligand signals for a range of very different complexes in addition to those of **2**. For example, a species with hydride ligand signals at δ –9.10 and –13.49 is seen, corresponding to known [Ir­(H)_3_(COD)­(IMes)] (**4**) (Figure 3).^58,59^ ^1^H NMR signals are also observed for a now dominant binuclear C–H bond activation product at δ –11.06 and –20.75 whose identity is confirmed by 2D NMR spectroscopy to be [Ir­(H)_2_(**A**)­(IMes)­(κ^2^-μ_2_-C,N-**A’**)_2_Ir­(H)_2_(**A**)­(IMes)] (**5**) (where **A’** is a C–H bond activated quinoxaline) (see Supporting Information, Section S1.2). A minor set of three signals for a related binuclear C–H bond activation product is also observed, at δ –8.21, –21.23, and –24.11. This product could not be fully characterized by 2D NMR due to its low abundance and high signal overlap of the quinoxaline and IMes resonances. However, its chemical shifts and NOE data are consistent with the species [Ir­(H)_2_(**A**)­(IMes)­(κ^2^-μ_2_-C,N-**A’**)_2_Ir­(Cl)­(H)­(**A**)­(IMes)] (**6**) (see Supporting Information, Section S1.3). When this solution is allowed to react further at room temperature, the signals for **4** rapidly disappear, while those of **5** increase and those of **6** remain largely stable. Additional minor hydride ligand signals are also visible in these measurements, but the species giving rise to these signals could not be confirmed from NMR experiments due to their low abundance, and in some cases, transient nature.

**Figure 3:**
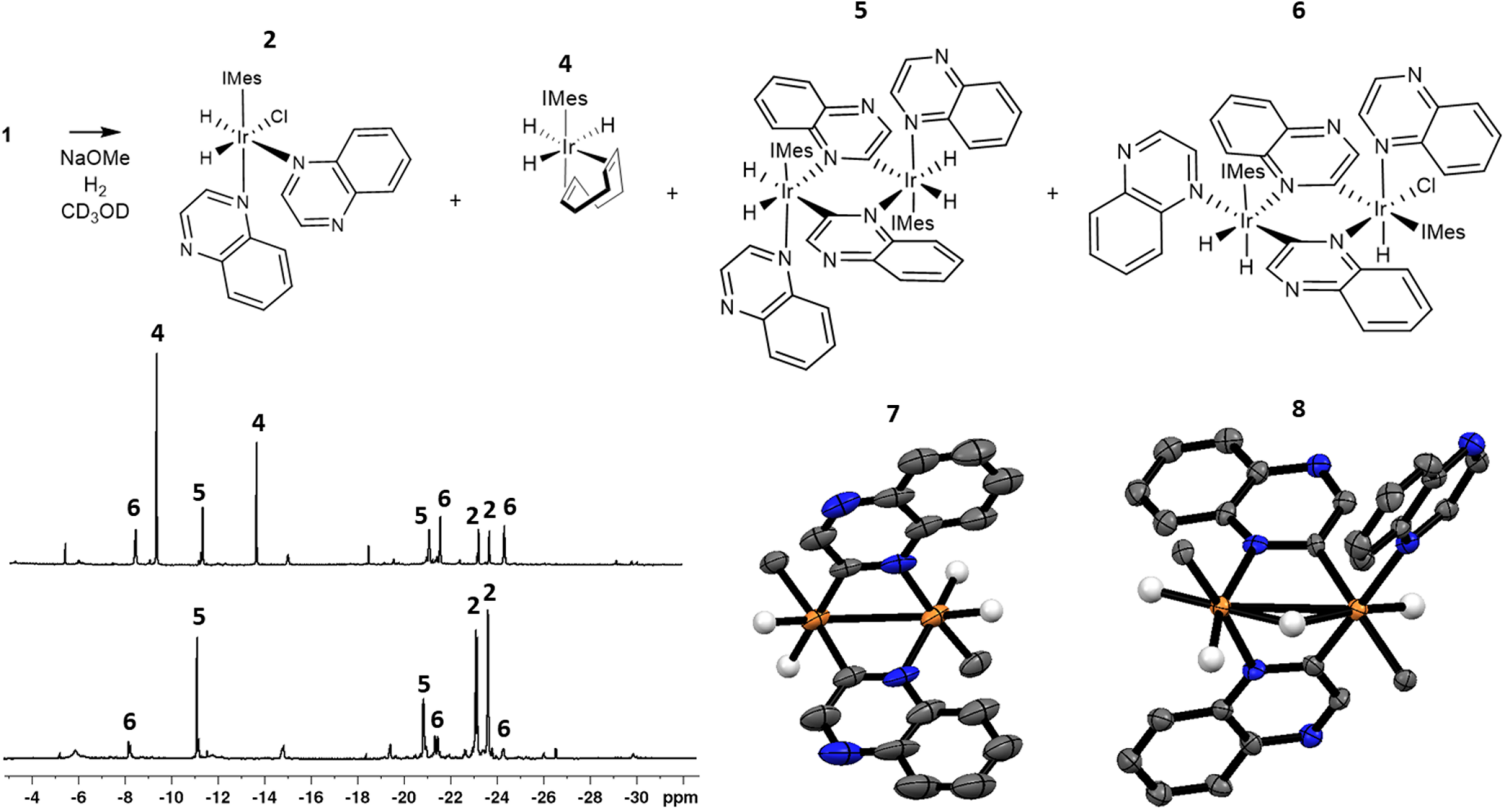
Reaction products formed upon the reaction of **A** (50 mM) with **1** (5 mM) and NaOMe (50 mM) and H_2_ (3 bar) in methanol-*d*_4_ (0.6 mL). Example ^1^H NMR spectra for the hydride region of these solutions after reaction for 30 min at room temperature before being cooled to 245 K (upper). The lower spectrum is recorded after the solution reacts for 2–3 h at room temperature before being cooled to 245 K. X-ray crystal structures of [Ir­(H)_2_(IMes)­(κ^2^-μ_2_-C,N-**A**)_2_Ir­(H)_2_(IMes)] (**7**) and [Ir­(H)_2_(IMes)­(κ^2^-μ_2_-C,N-**A**)_2_(μ_2_–H)­Ir­(H)­(**A**)­(IMes)] (**8**) are shown with thermal ellipsoids at 50% probability. White, gray, orange, and blue atoms correspond to hydrogen, carbon, iridium, and nitrogen, respectively. All nonhydride hydrogen atoms and solvent of crystallization are omitted for clarity. Only the carbene carbon of the IMes ligand is shown, with the rest of the ligand omitted for the sake of clarity. Crystal refinement details can be found in the Supporting Information, Section S2.1.

Interestingly, several species proved to precipitate from these solutions as single crystals when they are left at 278 K for several weeks. These were characterized by using X-ray diffraction. Notably, they reflect the neutral binuclear C–H bond activation products [Ir­(H)_2_(IMes)­(κ^2^-μ_2_-C,N-**A’**)_2_Ir­(H)_2_(IMes)] (**7**) and [Ir­(H)_2_(IMes)­(κ^2^-μ_2_-C,N-**A’**)_2_(μ_2_–H)­Ir­(H)­(**A**)­(IMes)] (**8**) (Figure 3 and Supporting Information Section S2.1). Both **7** and **8** are likely products of **5** and **6**, if they react further through the release of **A** and Cl^–^, with the metal centers of the products increasing their electron density through the formation of Ir–Ir bonds; NMR signals for these low solubility products are not discerned in the solution state NMR measurements.

More specifically, the loss of what must be a sterically costly **A** ligand to form **7** and **8** is accompanied by a rearrangement of the binding mode of the bridging C–H bond-activated quinoxaline ligands such that pairs of Ir–C and Ir–N interactions are now localized on the same metal center. In **7**, both metal centers achieve an 18-electron count, despite being formally Ir­(II) and Ir­(IV) as a consequence of the Ir–Ir bond. In contrast, **8** retains one of the original η^1^-**A** ligands, alongside a bridging hydride ligand, which again allows both metal centers to remain Ir­(III). π-stacking interactions between the quinoxaline and IMes mesityl groups within the crystal structure of **7** are likely maximized by these orientations. Interestingly, the 3.022 Å Ir–Ir bond length is longer than reported for other Ir–Ir dimers^29^ and clusters.^59^ The inclusion of the terminal quinoxaline ligand in **8** limits π stacking interactions such that more methanol solvent and now quinoxaline are required to fill the packing voids (see Supporting Information, Section S2.1). Complexes **7** and **8** are both catalytically competent, as when they are fully dissolved in DCM-*d*_2_ and reacted with fresh **A**, NaOMe, and D_2_, ^1^H NMR signals for HD and D_2_ were observed at room temperature after a few hours reaction, which suggests that H_2_ and hydride ligand sites within **7** and **8** are able to interchange. Furthermore, when these solutions are heated at 308 K for 3 days, 64% deuteration for the H_a_ site of **A** is evident, suggesting that they are themselves able to catalyze substrate deuteration or lead to species that can. In fact, when this reaction was continued for 1 week, the H_a_ site deuteration increased to 78%, and now 11% and 2% deuteration of the H_b_ and H_c_ sites, respectively, are achieved. By 3 weeks, these values had plateaued at 86%, 28%, and 11%, respectively.

Collectively, the ready formation of these C–H bond activation products (**5**-**8**) with a base suggests that unstable trihydride species **4** plays an important role in their formation. For example, if transient **4** were to lead to a monodeuteride complex by dihydrogen loss, subsequent C–H bond activation would lead to an Ir­(H)­(D)­(C) core. Hence, under what is effectively D_2_, any reversibility in the C–H bond activation process would then account for the observed HIE required to form labeled **A** and **B**.

### Extension to Deuterate a Wider Range of Targets

We completed this study by showing that this approach can be used to deuterate a wide range of molecules. For example, we show that isoquinoline (**C**), 3,5-dibromopyridine (**D**), 2-phenylpyridine (**E**), 2,5-lutidine (**F**), nicotine (**G**), caffeine (**H**), nicotinamide (**I**), isozianid (**J**), anastrazole (**K**), trimethoprim (**L**), and bisacodyl (**M**) can all be deuterated in methanol-*d*_4_ (0.6 mL) when solutions of them (50 mM) are brought into contact with **1** (5 mM), NaOMe (50 mM), and H_2_ (3 bar) (Figure 4). Their deuteration was confirmed by ^1^H NMR spectroscopy, which reveals the expected decreases in the ^1^H NMR signal intensity for the associated deuterated sites, and from mass spectrometry, which yields molecular ion peaks spanning several mass units higher than those of the *protio* isotopologue, depending on how many deuterium labels are incorporated (Supporting Information, Section S3). In some cases, facile deuteration can be achieved without NaOMe, although the inclusion of NaOMe promotes the process. Generally, the sites *ortho* to the substrate nitrogen are most readily deuterated with those in **C**, **F**, **G**, **I**, and **L** attaining over 90% deuteration in our time limited reactions. The highest deuteration levels are achieved for pyridines (**B**, **D**, **E**, **F**, **G**, **I**, **J** and **M**), although other *N*-heterocyclic scaffolds can also be used such as quinoxalines (**A**), isoquinolines (**C**), purine/xanthines (**H**), triazoles (**K**), and pyrimidines (**L**). Notably, this leads to 92% selective deuteration for the aromatic proton in the pyrimidine ring of **L**, which is an antibiotic used to treat a range of conditions, including bladder and ear infections, diarrhea, and even HIV/AIDS. Significant (>80% deuteration) can also be achieved for anastrozole (**K**), an estrogen blocker used to treat breast cancer, and bisacodyl (**M**), a commonly prescribed laxative. Deuteration of **K** is selective to the triazole ring, and site-selective deuteration is achieved in **M**.

**Figure 4:**
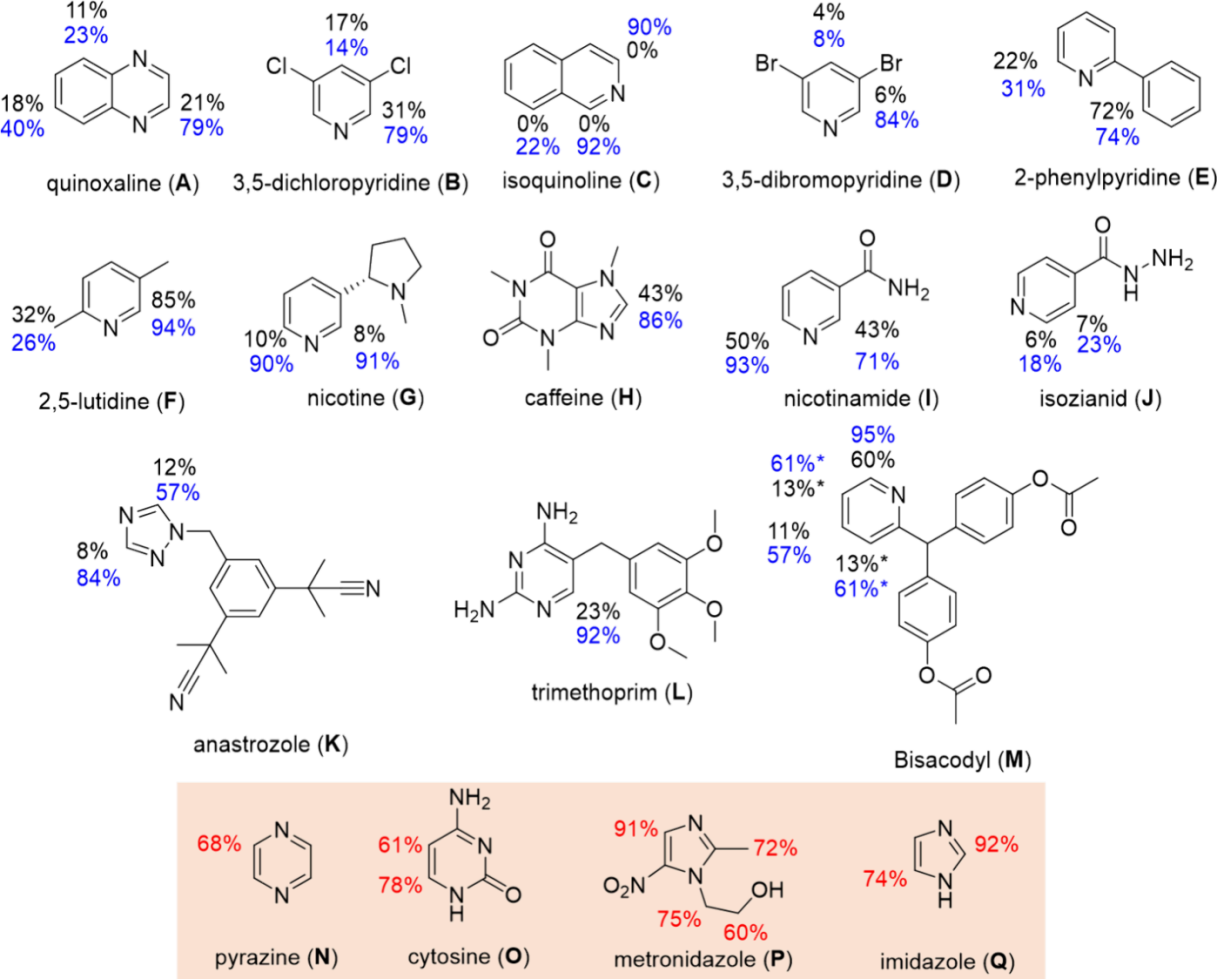
Substrates used in this work with % deuteration of the ^1^H sites determined from ^1^H NMR spectroscopy are shown. Values in black are achieved after reaction with no added base for 24 h at 298 K (50 mM substrate with **1** (5 mM) and H_2_ (3 bar) in methanol-*d*_4_ (0.6 mL)), whereas values in blue are recorded under analogous conditions but with NaOMe added (50 mM). *Indicates signal overlap. Those substrates highlighted in the red box could only be deuterated when they (50 mM) were reacted for 24 h at 298 K (50 mM substrate with preformed **4** (prepared after reaction of **1** (5 mM) with NaOMe (50 mM) and H_2_ (3 bar) in methanol-*d*_4_ (0.6 mL) for *ca* 18 h at 253 K).

In all of these reaction mixtures, ^1^H NMR spectroscopy reveals the presence of **4** in the early stages of the reaction, which further supports the suggestion that it forms on the route to the C–H bond activation products needed for substrate deuteration. It is also worth noting that a series of other molecular scaffolds were examined, containing imidazole, indole, and benzimidazole functionality. Substrates containing these groups were not deuterated when reacted with **1**, NaOMe, H_2,_ and methanol-*d*_4_. In these cases, no ^1^H NMR signals were observed for **4**, which has already been linked to stronger substrate binding for molecules like imidazole.^59^ Complex **4** has been suggested to form from the deprotonation of an [Ir­(H)_2_(η^2^-H_2_)­(COD)­(IMes)]^+^ intermediate by base.^59^ This intermediate forms after the loss of substrate from [Ir­(substrate)­(H)_2_(COD)­(IMes)]^+^ and subsequent binding of H_2_. The role of the base in this deuteration process is therefore linked to deprotonation of the dihydride dihydrogen intermediate required to form trihydride **4**, which goes on to form HIE catalysts. Accordingly, metal–ligand bond strength is a key parameter in determining how much **4** can form, and strongly ligating substrates, such as imidazole, can disfavor the formation of **4** completely. These observations further suggest that **4** likely acts as a precursor to other species containing C–H activated substrates (**5**-**8** in the case of **A**) that play a role in the deuteration of the substrate. To confirm the role of **4**, substrates **N**-**P** (50 mM) were reacted with unstable **4** that had been preformed from **1** (5 mM) in NMR scale reactions involving NaOMe and H_2_ in methanol-*d*_4_ overnight at 253 K. Consequently, in these reactions, a strongly ligating target can no longer prevent formation of **4**, and when these deuteration reactions are allowed to react for 24 h at room temperature, significant deuteration for **N**-**P** is observed (Figure 4), an observation which is particularly notable given that no deuteration was achieved when these substrates are reacted with precatalyst **1** and base. Despite this refined approach, structures with indole and benzimidazole functionalities were still undeuterated.

Single crystals were also obtained from solutions containing **B**, **D**, and **E** and found by X-ray diffraction to correspond to [Ir­(H)_2_(IMes)­(κ^2^-μ_2_-C,N-**B’**)_2_(μ_2_–H)­Ir­(H)­(**B**)­(IMes)] (**9**), [Ir­(Cl)­(H)­(IMes)­(κ^2^-μ_2_-C,N-**B’**)_2_(μ_2_–H)­Ir­(H)­(**B**)­(IMes)] (**10**), [Ir­(H)_2_(IMes)­(κ^2^-μ_2_-C,N-**D’**)_2_Ir­(H)_2_(IMes)] (**11**) and [Ir­(κ^2^-**E’**)_2_(H)­(IMes)] (**12**) where X’ denotes a C–H bond activated variant of X (Figure 5, Supporting Information Sections S2.2 and S2.3 for crystallographic details). These products again all contain C–H bond-activated substrates and are not obtained from analogous solutions in the absence of NaOMe. Species **9**, **10,** and **11** are directly analogous to **8** and **7**, respectively, while **12** reflects a mononuclear Ir­(III) product that results from the binding and activation of two molecules of 2-phenylpyridine (**E**) to a single metal center. These results confirm the clear potential of these Ir­(NHC)-derived complexes to facilitate C–H bond activation reactions.

**Figure 5:**
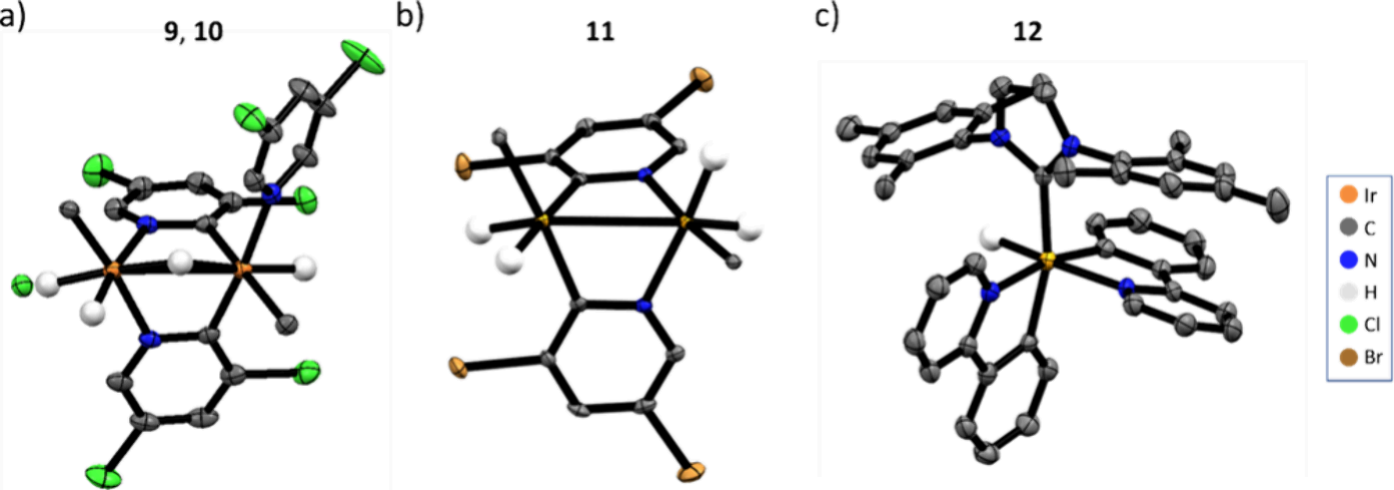
X-ray crystal structures of (a) [Ir­(H)_2_(IMes)­(κ^2^-μ_2_-C,N-**B**)_2_(μ_2_–H)­Ir­(H)­(**B**)­(IMes)] (**9**), and [Ir­(Cl)­(H)­(IMes)­(κ^2^-μ_2_-C,N-**B**)_2_(μ_2_–H)­Ir­(H)­(**B**)­(IMes)] (**10**) which are disordered at 41 and 59% occupancy, respectively, within the unit cell, (b) [Ir­(H)_2_(IMes)­(κ^2^-μ_2_-C,N-**D**)_2_Ir­(H)_2_(IMes)] (**11**) and (c) [Ir­(κ^2^-**E**)_2_(H)­(IMes)] (**12**). Thermal ellipsoids are shown at 50% probability, and all nonhydride hydrogen atoms and solvent of crystallization are omitted for clarity. Only the carbene carbon of the IMes ligand is shown in (a) and (b), with the rest of this ligand omitted for clarity. Crystal refinement details can be found in the Supporting Information, Section S2.

## Experimental Section

### Safety Statement

Caution! The NMR-scale reaction procedure controlled the risk by using millimolar concentrations of chemicals. Please refer to the SDS of the individual substrates for the individual hazards.

Caution! Extreme care should be taken in both the handling of the cryogen liquid nitrogen and its use in the Schlenk line trap to avoid the condensation of oxygen from air.

Caution! High-pressure hydrogen (3 bar) was used in this procedure. Extreme care should be taken when handling pressurized NMR tubes. Hydrogen is classified as a GHS Flammable Gas, Category 1- keep away from open flames, hot surfaces, and sources of ignition.

### General Information

All starting compounds were purchased from Sigma-Aldrich, Fluorochem, or Alfa-Aesar and used as supplied without further purification. [IrCl­(COD)­(IMes)] was synthesized in our laboratory according to a literature procedure.^60^ Samples were prepared in a 5 mm NMR tube that was fitted with a J. Young’s tap. The NMR samples were subsequently degassed by three freeze–pump–thaw cycles using liquid nitrogen on a Schlenk line. ^1^H NMR spectra were then recorded, and the reaction commenced at room temperature by filling the tube with H_2_. The reaction was left to proceed in the NMR tube at room temperature for the indicated time period (usually 24 h) before a ^1^H NMR spectrum was taken to assess the level of deuteration. The % deuteration values were determined by dividing the ^1^H NMR signal integral of the site after 24 h reaction normalized to the integral of an internal standard (grease at δ 0.12) by the corresponding value recorded before the reaction commenced. In cases where the deuteration time course was monitored using NMR spectroscopy, the NMR tube was left to react at 298 K inside a 9.4 T NMR spectrometer, and ^1^H NMR spectra were recorded every 90 s for 13 h. Control experiments were performed on methanol-*d*_4_ solutions (0.6 mL) of **A** or **B** (50 mM) with increasing amounts of NaOMe solution (50–200 mM in 50 mM increments), and ^1^H NMR spectroscopy revealed a negligible change in chemical shift. 2D NMR characterization of **2** was achieved upon reaction of **A** (50 mM) with **1** (5 mM) and NaOMe (50 mM) and H_2_ (3 bar) in methanol-*d*_4_ (0.6 mL) for 30 min at room temperature before being cooled to 245 K. **5** and **6** were characterized by repeating this process but extending the reaction time to 3 h before cooling to 245 K for characterization.

All NMR measurements were carried out on a 400 MHz Bruker Avance III spectrometer at 298 K unless otherwise stated. Chemical shifts are quoted as parts per million and referenced to the residual solvent. Coupling constants (*J*) are quoted in Hertz. At this point, electrospray mass spectra were recorded on a Bruker Daltronics microOTOF spectrometer to confirm the extent of deuteration after 24 h reaction. Deuteration of **A**, **B,** and **E** with and without base was performed three times. Accordingly, the deuteration of the other substrates was performed once.

### General Procedure A for NMR-Scale HIE Reactions^61–63^

**1** (2 mg) and the indicated substrate (10 equiv relative to **1**) were dissolved in methanol-*d*_4_ (0.6 mL) to give final catalyst concentrations of 5 mM and 50 mM. The solution was degassed via three freeze–pump–thaw cycles using a Schlenk line before filling the tube with H_2_ (3 bar). For samples including base, a 25 wt % solution of NaOMe in methanol was added to give a NaOMe concentration of 50 mM. ^1^H NMR spectra were collected before the addition of H_2,_ and both ^1^H NMR and mass spectrometry were performed after 24 h reaction at room temperature. Control experiments involving methanol-*d*_3_ or methanol-*d* as the solvent instead of methanol-*d*_4_ were performed following the same general procedure A. Control experiments involving D_2_ gas, instead of H_2_, also follow the same general procedure. In this case, a cannister of D_2_ gas with a regulator set to 3 bar was connected to our Schlenk line, and the line was filled with D_2_ gas. The degassed NMR sample was connected to the Schlenk line, and the tube was opened to allow D_2_ to enter.

### General Procedure B for NMR-Scale HIE Reactions

**1** (2 mg) and NaOMe (7.2 μL of a 25 wt % in methanol, 10 equiv relative to **1**) were dissolved in methanol-*d*_4_ (0.5 mL). The solution was degassed via three freeze–pump–thaw cycles using a Schlenk line before filling the tube with H_2_ (3 bar), and reacting at 253 K for 16 h overnight to form [Ir­(H)_3_(COD)­(IMes)], which was confirmed by ^1^H NMR spectroscopy at 253 K. NMR data for **4** has been reported previously.^59^ At this point, the tube was warmed to 298 K, and the indicated substrate (10 equiv relative to **1**) in methanol-*d*_4_ (0.1 μL) was added to the NMR tube to give final Ir concentrations of 5 mM, substrate concentrations of 50 mM, and base concentrations of 50 mM. A ^1^H NMR spectrum was collected, and the NMR tube was repressurized with H_2,_ and the reaction was allowed to proceed for 24 h at room temperature.

More specific synthetic details are given in the Supporting Information, Section S3 with mass spectra and ^1^H NMR spectra presented in Sections S4 and S5 respectively.

### X-ray Crystallography

Suitable crystals were prepared by leaving the NMR tube in the refrigerator for several weeks to precipitate single crystals. These were selected and mounted on either an Oxford-Diffraction SuperNova dual-source X-ray diffractometer equipped with copper and molybdenum sources and a HyPix-6000HE detector with cooling using an Oxford Instruments Cryojet (for **7**-**10**) or a Rigaku XtaLAB Synergy-S X-ray diffractometer equipped with a copper source and a HyPix-Arc 100° detector with cooling using an Oxford Cryosystems Cryosteam-1000 (for **11** and **12**). Using Olex2,^61^ the structures were solved with the SHELXT^62^ structure solution program using Intrinsic Phasing and refined with the SHELXL^63^ refinement package using least squares minimization. Details of the structural refinement and key parameters of the unit cell(s) are given in the Supporting Information.

Crystals of **7** and **8** could be grown in sufficient quantities to allow their catalytic competency to be examined. Crystals were collected from a methanol-*d*_4_ solution by removing the majority of the solvent using a metal syringe needle. A flow of N_2_ gas was then passed through a syringe needle and over the crystals at the bottom of the tube to remove any remaining methanol. At this point, the crystals were fully dissolved in DCM-*d*_2_ and reacted with fresh **A** (50 mM), NaOMe (50 mM), and D_2_ (3 bar) analogous to general procedure A using the crystals in place of **1**.

## Conclusions

This study demonstrates the efficient deuteration of a range of *N*-heterocycles, thereby showcasing significant functional group tolerance. This deuteration was achieved by using the air-stable iridium catalyst [IrCl­(COD)­(IMes)] and H_2_ in methanol-*d*_4_, with the addition of NaOMe as a base. While the deuteration process proceeds even without NaOMe, its efficiency is dramatically enhanced by the base. Mechanistically, the HIE process proceeds via initial hydride-deuterium exchange with the OD label of the solvent, forming intermediates such as [Ir­(H)_2_(IMes)­(substrate)­(methanol-*d*_4_)], which enable Ir-D bonds to form. Further mechanistic insights were then gained through the identification of catalytically active species during the reaction. Notably, a number of binuclear and mononuclear C–H bond-activated Ir complexes were observed and characterized by NMR spectroscopy and X-ray crystallography, where possible.

Significantly, these products prove to be most readily accessed via solutions in which the trihydride complex [Ir­(H)_3_(COD)­(IMes)] is formed initially in appreciable amounts due to the addition of NaOMe. Furthermore, when molecular scaffolds containing imidazole, indole, and benzimidazole functionalities are examined, no product deuteration is observed. These reactions correspond to situations where the formation of [Ir­(H)_3_(COD)­(IMes)] is suppressed. For imidazole, deuteration could be achieved when preformed [Ir­(H)_3_(COD)­(IMes)] was used as the catalyst instead of [IrCl­(COD)­(IMes)]. The high reactivity of [Ir­(H)_3_(COD)­(IMes)] has recently been described, where its use as a source of {Ir­(H)_3_(IMes)} type building blocks plays a significant role in its chemistry.^58,59^ These results reported here suggest that [Ir­(H)_3_(COD)­(IMes)] acts to promote HIE catalysts by providing access instead to {IrH­(IMes)} fragments, the logical product of H_2_ loss from {Ir­(H)_3_(IMes)}. This observation is supported by the fact that X-ray structures were obtained for its potential trapped products [Ir­(H)_2_(IMes)­(κ^2^-μ_2_-C,N-**A**)_2_Ir­(H)_2_(IMes)] (**7**), [Ir­(H)_2_(IMes)­(κ^2^-μ_2_-C,N-**A**)_2_(μ_2_–H)­Ir­(H)­(**A**)­(IMes)] (**8**), [Ir­(H)_2_(IMes)­(κ^2^-μ_2_-C,N-**B**)_2_(μ_2_–H)­Ir­(H)­(**B**)­(IMes)] (**9**), [Ir­(Cl)­(H)­(IMes)­(κ^2^-μ_2_-C,N-**B**)_2_(μ_2_–H)­Ir­(H)­(**B**)­(IMes)] (**10**), and [Ir­(H)_2_(IMes)­(κ^2^-μ_2_-C,N-**D**)_2_Ir­(H)_2_(IMes)] (**11**), which contain two {IrH­(IMes)} building blocks and C–H bond activated substrate(s). In view of this hypothesis, single crystals of **7** and **8** proved able to catalyze the HIE process in suitable control experiments. Hence, a route to {IrH­(IMes)}, and therefore its deuterated counterpart {IrD­(IMes)} is deduced to be important for HIE catalysis by [IrCl­(COD)­(IMes)]. Furthermore, a role for the 16-electron intermediate {Ir­(H)­(IMes)­(L)­2} [L = substrate] is indicated. While mechanisms for the HIE reaction have been suggested previously,^25,44–47^ we have chosen not to include a further cycle here due to the complexity of this reaction and the propensity for these complexes to react further to form higher order clusters.^59^ We feel that the mechanism warrants further investigation before it can be reliably described.

High levels of deuteration were achieved for the pharmaceuticals anastrozole (84%), trimethoprim (92%), and bisacodyl (95%), with 100% site selectivity in the case of trimethoprim. Notably, all of these experiments were conducted at room temperature, underscoring the remarkable activity of {IrD­(IMes)} and its precursor [Ir­(H)_3_(COD)­(IMes)] in HIE catalysis. Although the reaction times were set to 24 h, they could likely be shortened by increasing the temperature. In order to further validate these measurements, the reported reactions with substrates **A**, **B,** and **E** were examined in triplicate for 22 and 46 h of reaction. The ^2^H levels achieved in the three samples, for any single substrate, provided comparable results, thereby confirming the statistical significance of our analyses. Furthermore, the levels of ^2^H labeling in **A** proved to increase from 57 to 97% at position H_a_ on moving from a reaction time of 22 to 46 h, thereby confirming that longer reaction times would lead to higher levels of ^2^H label incorporation (see ESI). In the case of quinoxaline, a further reaction was completed on a 200 mg scale over 8 days that led to an isolated yield of 66% with 99% ^2^H labeling at position H_a_, 86% at H_b_, and 85% at H_c_.

In this work, we have chosen to focus on using nonforcing conditions and believe the attainment of high deuteration % in room temperature conditions is a key benefit to our work and could provide cost and environmental benefits compared to shorter reaction times achieved at higher temperatures. Further optimization could be achieved by varying parameters such as the concentration of reagents, the proportion of the deuterium label (by using a greater excess of methanol-*d*_4_), the choice of base, and the steric or electronic properties of the carbene ligand (NHC), which will directly affect the reactivity of {IrH­(NHC)} and [Ir­(H)_3_(COD)­(NHC)]. However, we note that significant deuteration (over 90%) could be achieved in many cases without further optimization. Since methods for removing the iridium-based catalysts from such systems have been established elsewhere^64,65^ there should be no significant barrier to purifying these deuterated products. As such, further refining this process could offer considerable advantages over alternative methods, particularly in terms of efficiency and scalability.

## Supplementary Material



## Data Availability

The data underlying this study are available in the published article, in its Supporting Information, and openly available in the University of York data repository at https://pure.york.ac.uk/portal/en/datasets/base-promoted-iridium-catalysed-deuteration-and-ch-bond-activatio

## Notes

^1^H NMR and MS spectra for **A**-**Q;** NMR characterization data for **2**, **5**-**6**; and X-ray crystallography details for **7**-**10**(PDF)
