# Supplementary material for: Base-Promoted Iridium-Catalyzed Deuteration and C–H Bond Activation of N‑Heterocycles
Source: J Org Chem. 2025 Jun 5;90(24):8080–9. doi: 10.1021/acs.joc.5c00174 (PMC12186604; doi:10.1021/acs.joc.5c00174)
Supplement: Supplementary file 1 [file jo5c00174_si_001.pdf]

Supporting Information

Base-promoted iridium-catalyzed deuteration and C-H bond activation of *N*-heterocycles

Ben. J. Tickner,[a,b] Claire Condon,[a,b] Victoria Annis,[a,b] Richard J. Gammons,[b]

Adrian C. Whitwood[b] and Simon B. Duckett*[a,b]

[a]Centre for Hyperpolarisation in Magnetic Resonance, University of York, Heslington, United Kingdom, YO10 5NY

[b]Department of Chemistry, University of York, Heslington, United Kingdom, YO10 5DD

*Corresponding author email: simon.duckett@york.ac.uk

SUPPORTING INFORMATION

Contents

S1: NMR Characterisation of samples containing quinoxaline (A) SI-3
S1.1: NMR Characterisation of **2** SI-3
S1.2: NMR Characterisation of **5** SI-4
S1.3: NMR Characterisation of **6** SI-5
S2: X-ray crystallography SI-5
S2.1: X-ray diffraction of **7** and **8** SI-5
S2.2: X-ray diffraction of **9** and **10** SI-8
S2.3: X-ray diffraction of **11** SI-10
S2.4: X-ray diffraction of **12** SI-12
S3: Synthetic details of Deuterium Labelling Experiments SI-14
S4: Mass Spectrometry of Deuterium Labelling Experiments SI-21
S4.1: Mass spectrometry of **A** SI-21
S4.2: Mass spectrometry of **B** SI-22
S4.3: Mass spectrometry of **C** SI-23
S4.4: Mass spectrometry of **D** SI-24
S4.5: Mass spectrometry of **E** SI-25
S4.6: Mass spectrometry of **F** SI-26
S4.7: Mass spectrometry of **G** SI-27
S4.8: Mass spectrometry of **H** SI-28
S4.9: Mass spectrometry of **I** SI-29
S4.10: Mass spectrometry of **J** SI-30
S4.11: Mass spectrometry of **K** SI-31
S4.12: Mass spectrometry of **L** SI-32
S4.13: Mass spectrometry of **M** SI-33
S4.14: Mass spectrometry of **O** SI-34
S4.15: Mass spectrometry of **P** SI-34
S5: ^{1}H NMR spectroscopy of Deuterium Labelling Experiments SI-35
S5.1: ^{1}H NMR spectroscopy of **A** SI-35
S5.2: ^{1}H NMR spectroscopy of **B** SI-36
S5.3: ^{1}H NMR spectroscopy of **C** SI-37
S5.4: ^{1}H NMR spectroscopy of **D** SI-38
S5.5: ^{1}H NMR spectroscopy of **E** SI-39
S5.6: ^{1}H NMR spectroscopy of **F** SI-40
S5.7: ^{1}H NMR spectroscopy of **G** SI-41
S5.8: ^{1}H NMR spectroscopy of **H** SI-42
S5.9: ^{1}H NMR spectroscopy of **I** SI-43
S5.10: ^{1}H NMR spectroscopy of **J** SI-44
S5.11: ^{1}H NMR spectroscopy of **K** SI-45
S5.12: ^{1}H NMR spectroscopy of **L** SI-46
S5.13: ^{1}H NMR spectroscopy of **N** SI-47
S5.14: ^{1}H NMR spectroscopy of **O** SI-47
S5.15: ^{1}H NMR spectroscopy of **P** SI-48
S5.16: ^{1}H NMR spectroscopy of **Q** SI-48
S6: Large Scale Deuteration of Quinoxaline (A) SI-49
S7: Triplicate Studies on Quinoxaline (A), 3,5-Dichloropyridine (B) and 2-Phenylpyridine (E) SI-50

S1: NMR Characterisation of samples containing quinoxaline (A)

S1.1: NMR Characterisation of **2**

The structure of **2** is shown in Figure S1 and its NMR resonances are detailed below.

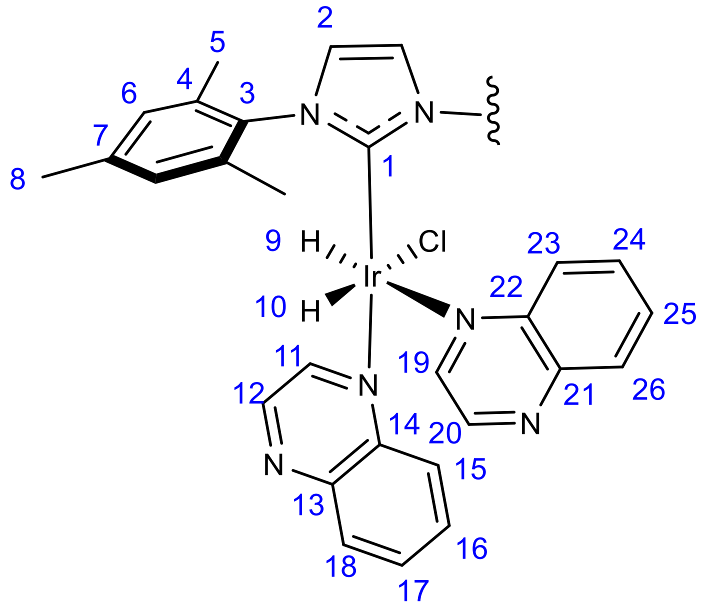

Figure S1: Structure of 2. Note that only one mesityl group of the IMes ligand is shown for clarity

^{1}H NMR (methanol-d_4, 400 MHz, 245 K): δ 9.19 (m, 1H, H-11), 8.60 (d, 1H, *J* = 8 Hz H-23), 8.18 (m, 1H, H-19), 7.93 (m, 1H, H-26), 7.89 (m, 1H, H-12), 7.65 (m, 1H, *J* = 8 Hz, H-25), 7.25 (m, 1H, H-24), 7.16 (s, 2H, H-2), 6.93 (s, 2H, H-6), 6.42 (s, 2H, H-6), 2.35 (s, 6H, H-8), 2.19 (s, 6H, H-5), 2.02 (s, 6H, H-5), −23.03 (d, 1H, $^{2}J_{HH}$ = 7 Hz, H-9), −23.50 (d, 1H, $^{2}J_{HH}$ = 7 Hz, H-10) ppm.
Note that a series of overlapping resonances between 7 and 9 ppm are also present which could not be definitively assigned to the individual H-15 to H-20 sites.

^{13}C {^{1}H} NMR (methanol-d_4, 125 MHz, 245 K): δ 151.71 (C-1), 145.30 (C-19), 138.08 (C-4), 136.16 (C-3), 135.31 (C-7), 133.96 (C-11), 130.20 (C-30), 129.20 (C-24), 128.30 (C-6), 128.19 (C-6), 122.23 (C-2), 19.85 (C-5), 17.74 (C-8), 17.59 (C-5) ppm.
Note that a series of overlapping resonances between 120 and 140 ppm are also present which could not be definitively assigned to the individual C-13 to C-18, C-20, C-21 to C-23 and C-26 sites.

At 298 K **2** appears with a single broad hydride resonance due to rapid exchange, but this splits into two resonances when cooled to 278 K. At 245 K these signals sharpen and four signals are present for the two inequivalent hydrides, and their deuterated counterparts (as methanol-d_4 solvent can lead to deuteration of the hydride ligand sites). Both hydrides are mutually coupled and close in space. Both hydrides show NOE connections to IMes resonances at δ 2.02 and 2.35 and a bound quinoxaline *ortho* signal at δ 9.19. The hydride *trans* to chloride at δ – 23.50 shows NOE effects to a second ligated quinoxaline at δ 8.18 and 8.60. The remaining resonances were located using a mixture of NOESY, HMQC/HSQC and COSY spectra.

S1.2: NMR Characterisation of **5**

The structure of **5** is shown in Figure S2 and its NMR resonances are detailed below.

^{1}H NMR (methanol-*d*$_4$, 400 MHz, 245 K): *δ* 9.45 (s, 2H, H-20), 9.01 (s, 2H, H-11), 7.75 (m, 2H, H-22), 7.66 (m, 4H, H-2), 7.36 (m, 2H, H-23), 7.02 (s, 4H, H-2), 6.98 (s, 8H, H-6), 2.39 (s, 12H, H-8), 1.82 (s, 24H, H-4), −11.06 (d, 2H, H-9), −20.75 (d, 2H, H-10) ppm.
Note that a series of overlapping resonances between 7.1 and 7.8 ppm are also present which could not be definitively assigned to the individual H-14 to H-17 and the H-24 and H-25 sites.

^{13}C {^{1}H} NMR (methanol-*d*$_4$, 125 MHz, 245 K): *δ* 169.21 (C-1), 144.03 (C-20), 141.30 (C-19), 137.70 (C-7), 136.14 (C-3), 135.06 (C-11), 134.98 (C-5), 126.13 (C-22), 125.47 (C-23), 129.10 (C-12), 128.69 (C-6), 112.40 (C-2), 18.64 (C-4), 18.34 (C-8) ppm.
Note that a series of overlapping resonances between 125 and 130 ppm are also present which could not be definitively assigned to the individual C-14 to C-17, C-21 and C-24 to C-26 sites.

The hydride ligand signals at *δ* – 11.06 and – 20.75 appear in a 1:1 ratio. ^{1}H DOSY measurements suggest that this species is a dimer. Control experiments involving use of [IrBr(COD)(IMes)], or NaOH base do not reveal a change in ^{1}H NMR shifts which confirm that the signal at *δ* – 20.75 does not lie *trans* to Cl or OMe. These chemical shifts are consistent with chemical shifts *trans* to carbon and nitrogen respectively, and it is likely the species reflects a symmetric dimer bridged by two C-H activated quinoxaline ligands. This is confirmed from 2D NMR characterisation at 245 K. The hydride ligand signal at *δ* – 11.06 is close in space to the other hydride, IMes resonances at *δ* 1.81 and 2.39, a signal at *δ* 9.00 which is consistent with an ortho proton of a bound quinoxaline. The hydride ligand signal at *δ* – 20.75 is close in space to the other hydride, an IMes resonance at *δ* 2.39, and the quinoxaline signal at *δ* 9.00. As this signal is close in space to both hydrides it is located on a quinoxaline ligand mutually *cis* to both hydrides. Furthermore, the hydride at *δ* – 20.75 is close in space to a resonance at *δ* 7.75 which is assigned to a bridging CH activated quinoxaline. The remaining ^{1}H and ^{13}C NMR resonances can be located from HMQC, HSQC, COSY and NOE measurements. The final structure is confirmed from additional NOE connections from the bridging quinoxaline at *δ* 7.75 to the IMes resonance at *δ* 2.39. Another bridging quinoxaline signal at *δ* 9.45 is connected to IMes resonances at *δ* 2.42 and 6.98. The non-bridging quinoxaline ligand signal at *δ* 9.00 couples to a resonance at *δ* 7.66 for the unsymmetric *ortho* position and this site shows NOE connections to IMes at *δ* 1.82 and 6.98.

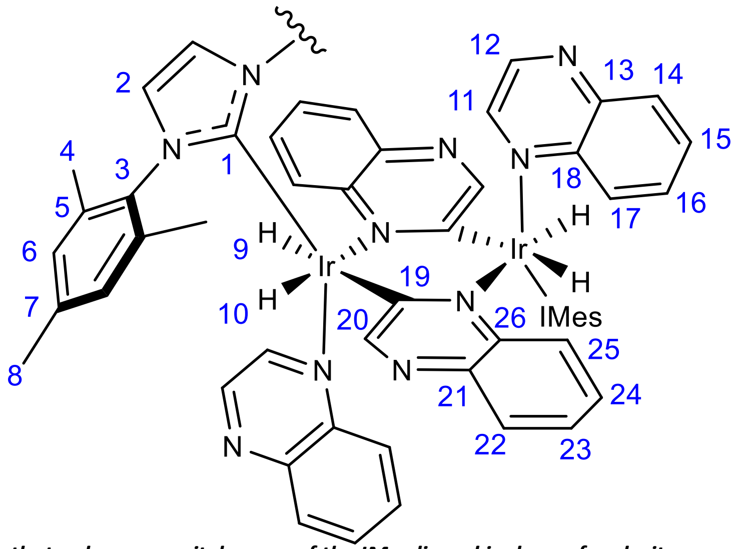

Figure S2: Structure of 5. Note that only one mesityl group of the IMes ligand is shown for clarity

S1.3: NMR Characterisation of 6

The structure of **6** is shown in Figure S3 and its NMR resonances are detailed below. **5** and **6** exist in equilibrium mixtures of the two complexes together.

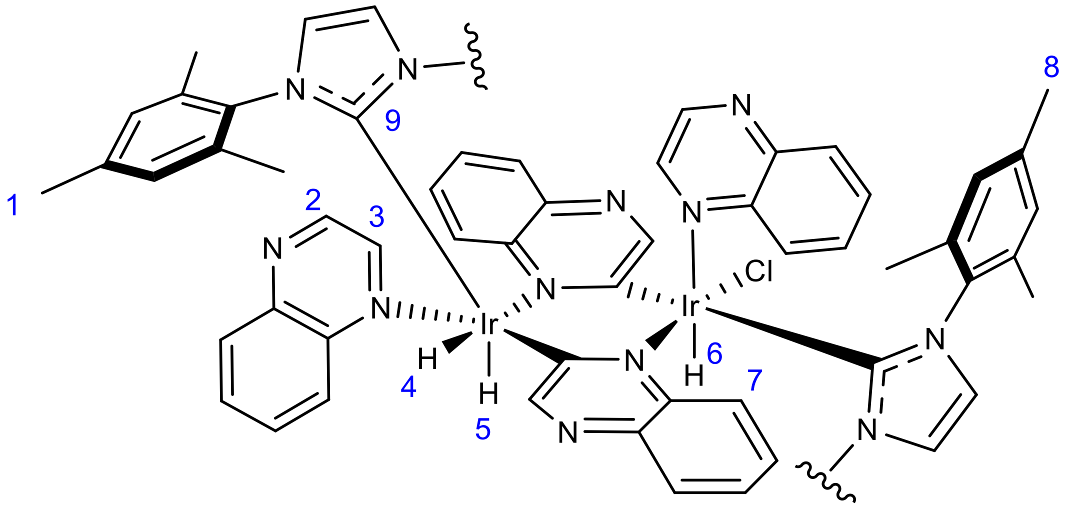

Figure S3: Structure of 6. Note that only one mesityl group of the IMes ligand is shown for clarity

^{1}H NMR (methanol-d_4, 400 MHz, 245 K): δ 9.47 (s, 1H, H-3), 8.52 (1H, H-2), 7.62 (m, 1H, H-7), 2.41 (s, 6H, H-1), 2.34 (s, 6H, H-8), –8.20 (*d*, 1H, $^{2}J_{HH}$ = 13 Hz, H-4), –21.22 (*d*, 1H, $^{2}J_{HH}$ = 13 Hz, H-5), –24.11 (*s*, 1H, H-6) ppm.

^{13}C {^{1}H} NMR (methanol-d_4, 125 MHz, 245 K): δ 164.55 (C-9), 148.88 (C-2), 144.19 (C-3), 129.08 (C-7), 17.66 (C-1) ppm.

The hydride ligand signals at δ – 8.20, – 21.22 and – 24.11 are all close in space from NOE connections. DOSY suggests the species is a dimer. Only the δ – 8.20, – 21.22 signals are coupled in a COSY and the two signals appear as doublets with a mutual 13 Hz J_{HH} coupling. Accordingly, they are likely located on the same Ir centre with the signal at δ– 24.11 corresponding to a hydride on a different Ir centre. Both hydrides at δ – 8.20, – 21.22 are close in space to an IMes at δ 2.41 with the other hydride at δ– 24.11 being close in space to an IMes at δ 2.34. The hydrides at δ – 8.20, – 21.22 are also both close in space to a bound *ortho* quinoxaline signal at δ 9.47. The hydride at δ– 24.11 is close in space to a signal at 7.62 which is likely from a bridging quinoxaline. Full characterisation data for this species could not be collected due to its low concentration and overlap of the IMes and quinoxaline signals with those for more dominant species in solution. These data are nonetheless consistent with **6**.

S2: X-ray crystallography

S2.1: X-ray diffraction of 7 and 8

A solution of **1** (5 mM), NaOMe (50 mM), and **A** (50 mM) were reacted with 3 bar H_2 in methanol-d_4 (0.6 mL) at room temperature for 24 hours in a J. Youngs tap NMR tube. At this point it was cooled to 278 K in a fridge and left for several weeks to form single crystals. These were found to be **7** and **8** and their crystallographic details are given in Table S1.

For **7** the hydrides were initially located by electron difference maps. The Ir-H bond lengths were restrained to be 1.7 angstroms and then the locations were allowed to refine. For **8**, hydrides were similarly located by electron difference maps. For the bridged hydride, the Ir-H bond-lengths were restrained to be equal. For terminal hydrides, the Ir-H bond-lengths were restrained to be 1.7 angstroms. The crystal contained some partially occupied and disordered solvent. There was a quinoxaline with a refined occupancy of 0.85(2), 0.48(2) and 0.38(2). For two of the solvent methanol molecules the C-O bond-lengths were restrained to be 1.42 angstroms (C77-O3 & C78-O4).

a)

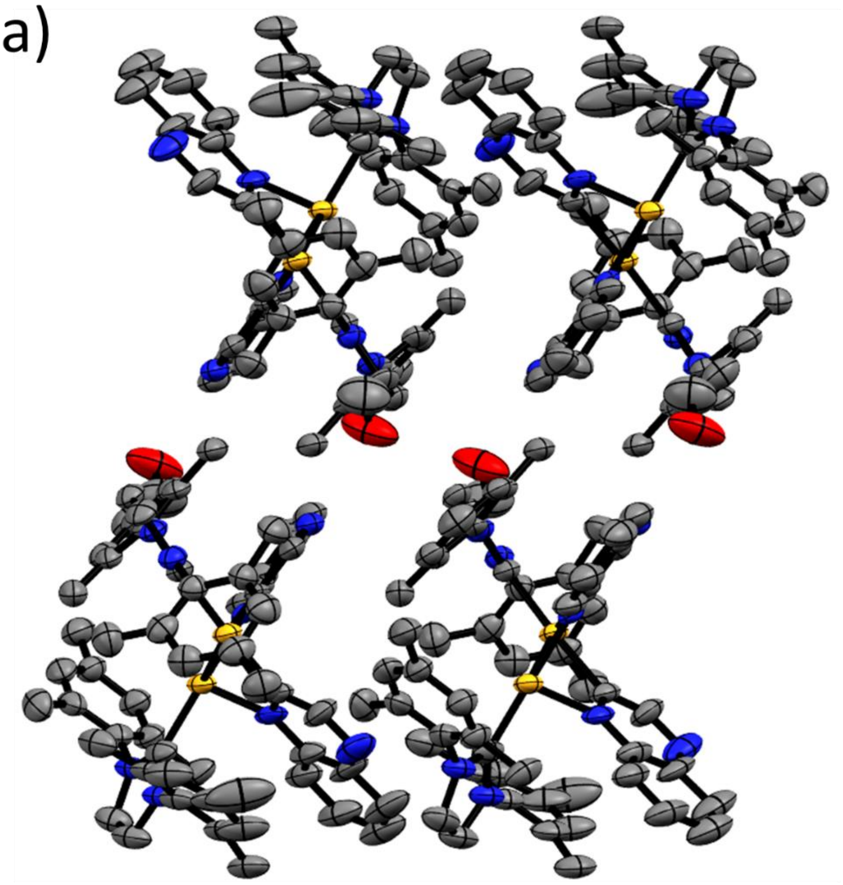

b)

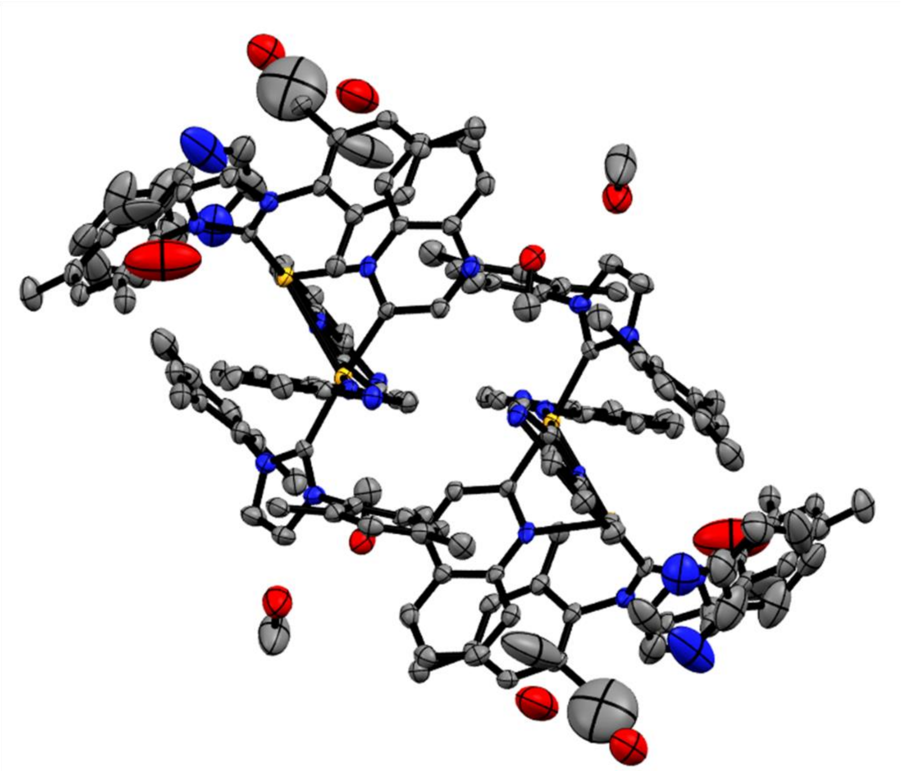

Figure S4: Crystal packing of a) 7 and b) 8 highlighting the role of pi stacking interactions within the crystal and the larger solvent-filled voids within crystals of 8 compared to 7. Thermal ellipsoids are shown at 50% probability.

Table S1: X-Ray crystallography details for 7 and 8.

Empirical formula	$C_{59}H_{66}Ir_2N_8O$	$C_{74.06}H_{86.12}Ir_2N_{11.09}O_{3.72}$
Formula weight/ Da	1287.59	1575.43
Temperature/K	111(2)	110.00(14)
Crystal system	monoclinic	triclinic
Space group	$P2_1/c$	P-1
a/Å	21.0753(11)	14.1785(3)
b/Å	15.6397(10)	14.5175(3)
c/Å	16.4568(13)	18.2615(3)
α/°	90	112.4703(18)
β/°	90.012(7)	95.9404(18)
γ/°	90	98.0147(18)
Volume/Å^3	5424.3(6)	3388.70(13)
Z	4	2
ρ_{calc}g/cm^3	1.577	1.544
μ/mm^{-1}	9.722	7.942
F(000)	2552.0	1584.0
Crystal size/mm^3	0.06 × 0.04 × 0.04	0.166 × 0.037 × 0.02
Radiation	Cu Kα (λ = 1.54184)	Cu Kα (λ = 1.54184)
2Θ range for data collection/°	7.798 to 134.142	7.582 to 136.498
Index ranges	-25 ≤ h ≤ 20, -15 ≤ k ≤ 18, -19 ≤ l ≤ 19	-17 ≤ h ≤ 17, -14 ≤ k ≤ 17, -21 ≤ l ≤ 16
Reflections collected	33785	37057
Independent reflections	9361 [R_{int} = 0.0555, R_{sigma} = 0.0480]	12356 [R_{int} = 0.0591, R_{sigma} = 0.0632]
Data/restraints/parameters	9361/5/658	12356/18/922
Goodness-of-fit on F^2	1.044	1.023
Final R indexes [I>=2σ (I)]	R_1 = 0.0585, wR_2 = 0.1304	R_1 = 0.0432, wR_2 = 0.0960
Final R indexes [all data]	R_1 = 0.0780, wR_2 = 0.1394	R_1 = 0.0627, wR_2 = 0.1039
Largest diff. peak/hole / e Å^{-3}	2.93/-1.65	1.74/-2.07

S2.2: X-ray diffraction of 9 and 10

A solution of **1** (5 mM), NaOMe (50 mM), and **B** (50 mM) were reacted with 3 bar H_2 in methanol-d_4 (0.6 mL) at room temperature for 24 hours in a J. Youngs tap NMR tube. At this point it was cooled to 278 K in a fridge and left for several weeks to form single crystals. These were found to contain **9** and **10** in the same unit cell and its crystallographic details are given in Table S2. The crystals contained a mixture of **9** and **10** and was modelled in a refined ratio of 0.408(4):0.592. Non-bridging Ir-hydride distances were restrained to be 1.7 angstroms. The methanol was modelled in two positions in a refined ratio matching that of **9** and **10**. The C-O bond length was restrained to be 1.41 angstroms.

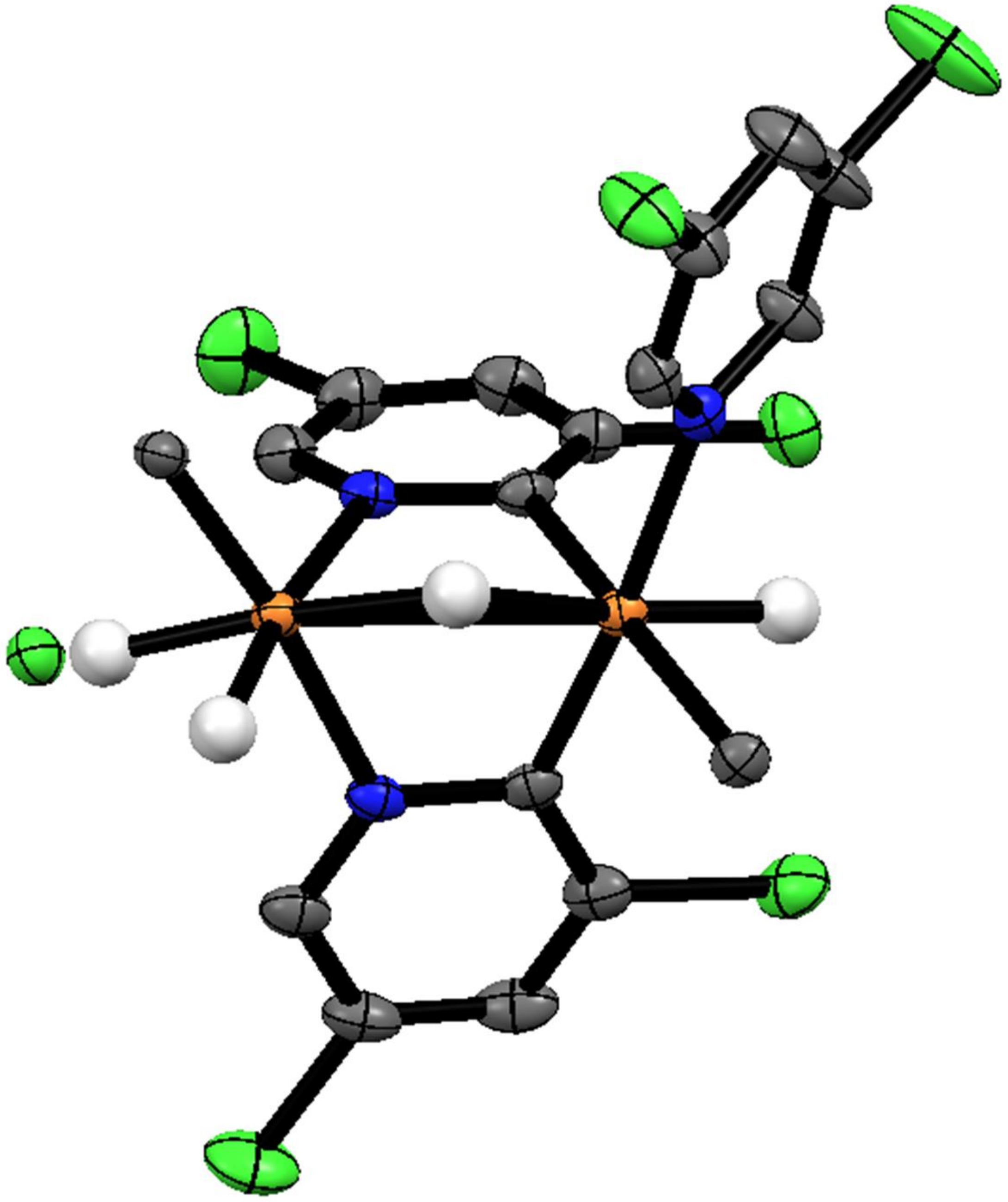

Figure S5: **X ray crystal structures of a) [Ir(H)$_2$(IMes)(κ^2-μ_2-C,N-B)$_2$(μ_2-H)Ir(H)(B)(IMes)] (9), and [Ir(Cl)(H)(IMes)(κ^2-μ_2-C,N-B)$_2$(μ_2-H)Ir(H)(B)(IMes)] (10) which are disordered at 41 and 59 % occupancy, respectively, within the unit cell,*Thermal ellipsoids are shown at 50% probability.***

Table S2: X-Ray crystallography details for crystals containing 9 and 10.

Empirical formula	$C_{58}H_{62.41}Cl_{6.59}Ir_2N_7O$
Formula weight/ Da	1491.57
Temperature/K	110.00(10)
Crystal system	monoclinic
Space group	C2/c
a/Å	40.9445(2)
b/Å	12.18284(8)
c/Å	23.35448(14)
α/°	90
β/°	99.7420(6)
γ/°	90
Volume/Å^3	11481.69(12)
Z	8
ρ_{calc}g/cm^3	1.726
μ/mm^{-1}	12.032
F(000)	5868.0
Crystal size/mm^3	0.15 × 0.11 × 0.06
Radiation	Cu Kα (λ = 1.54184)
2Θ range for data collection/°	7.682 to 134.156
Index ranges	-45 ≤ h ≤ 48, -11 ≤ k ≤ 14, -25 ≤ l ≤ 27
Reflections collected	52158
Independent reflections	10239 [R_{int} = 0.0441, R_{sigma} = 0.0298]
Data/restraints/parameters	10239/5/727
Goodness-of-fit on F^2	1.045
Final R indexes [I>=2σ (I)]	R_1 = 0.0257, wR_2 = 0.0623
Final R indexes [all data]	R_1 = 0.0291, wR_2 = 0.0642
Largest diff. peak/hole / e Å^{-3}	1.57/-0.87

S2.3: X-ray diffraction of **11**

A solution of **1** (5 mM), NaOMe (50 mM), and **D** (50 mM) were reacted with 3 bar H_2 in methanol-d_4 (0.6 mL) at room temperature for 24 hours in a J. Youngs tap NMR tube. At this point it was cooled to 278 K in a fridge and left for several weeks to form single crystals. These were found to contain **11** and its crystallographic details are given in Table S3.

The crystals contained disordered solvent for which a suitable discrete atom model could not be obtained. This was modelled using a solvent mask with a predicted volume of 184 cubic angstroms per asymmetric unit containing 70 electrons. This is equivalent to nearly 4 methanol molecules.

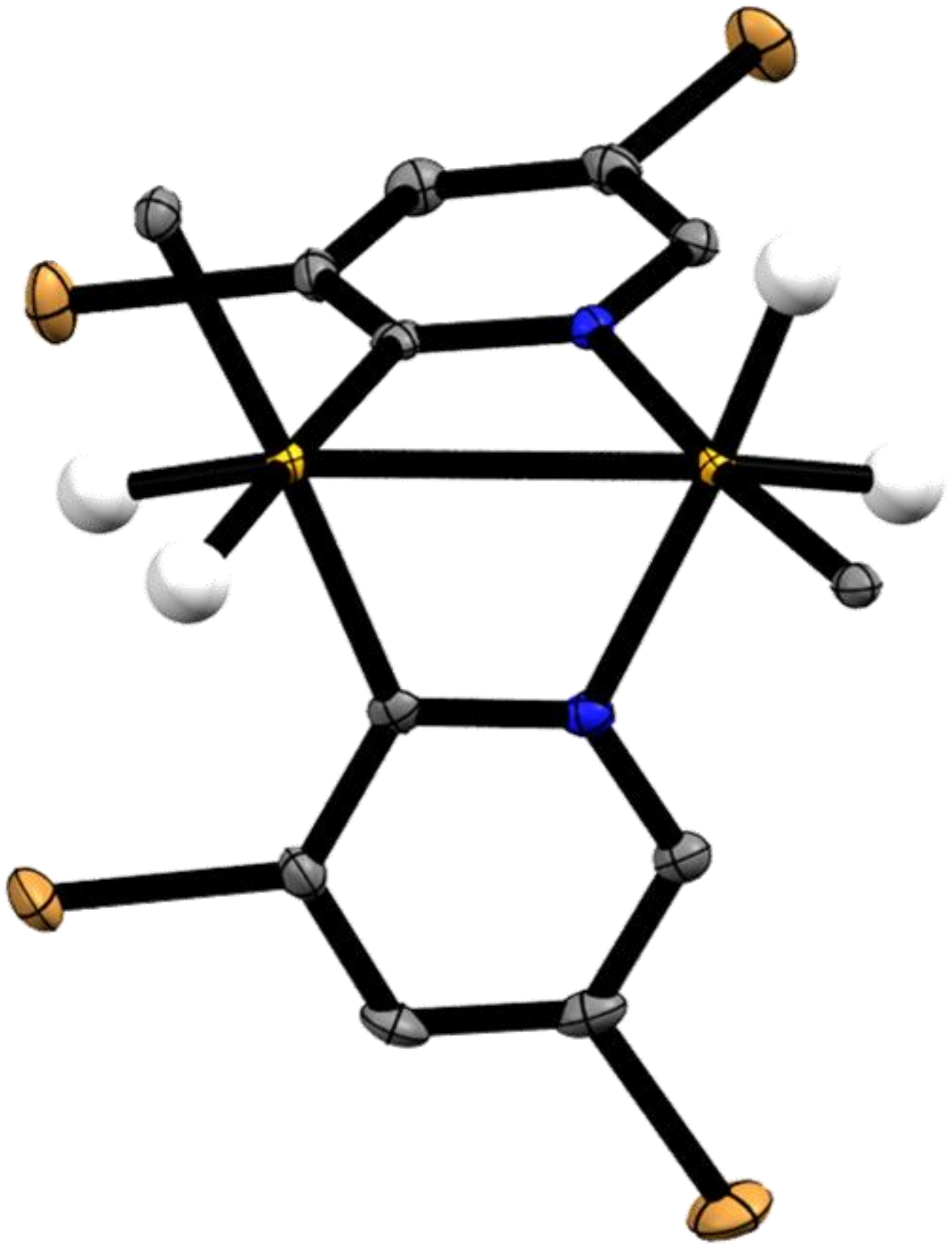

Figure S6: X ray crystal structure of 11. Thermal ellipsoids are shown at 50% probability.

Table S3: X-Ray crystallography details for crystals containing 11

Empirical formula	$C_{52}H_{56}Br_4Ir_2N_6$
Formula weight/ Da	1469.06
Temperature/K	110.00(10)
Crystal system	triclinic
Space group	P-1
a/Å	11.3377(2)
b/Å	12.4489(3)
c/Å	21.4959(4)
α/°	83.168(2)
β/°	78.696(2)
γ/°	69.159(2)
Volume/Å^3	2776.45(11)
Z	2
ρ_{calc}g/cm^3	1.757
μ/mm^{-1}	12.804
F(000)	1408.0
Crystal size/mm^3	0.172 × 0.126 × 0.099
Radiation	Cu Kα (λ = 1.54184)
2Θ range for data collection/°	7.61 to 136.5
Index ranges	-12 ≤ h ≤ 13, -13 ≤ k ≤ 14, -25 ≤ l ≤ 25
Reflections collected	30594
Independent reflections	10164 [R_{int} = 0.0194, R_{sigma} = 0.0162]
Data/restraints/parameters	10164/0/605
Goodness-of-fit on F^2	1.120
Final R indexes [I>=2σ (I)]	R_1 = 0.0211, wR_2 = 0.0498
Final R indexes [all data]	R_1 = 0.0213, wR_2 = 0.0499
Largest diff. peak/hole / e Å^{-3}	0.84/-0.86

S2.4: X-ray diffraction of **12**

A solution of **1** (5 mM), NaOMe (50 mM), and **E** (50 mM) were reacted with 3 bar H_2 in methanol-d_4 (0.6 mL) at room temperature for 24 hours in a J. Youngs tap NMR tube. At this point it was cooled to 278 K in a fridge and left for several weeks to form single crystals. These were found to contain **12** and its crystallographic details are given in Table S4.

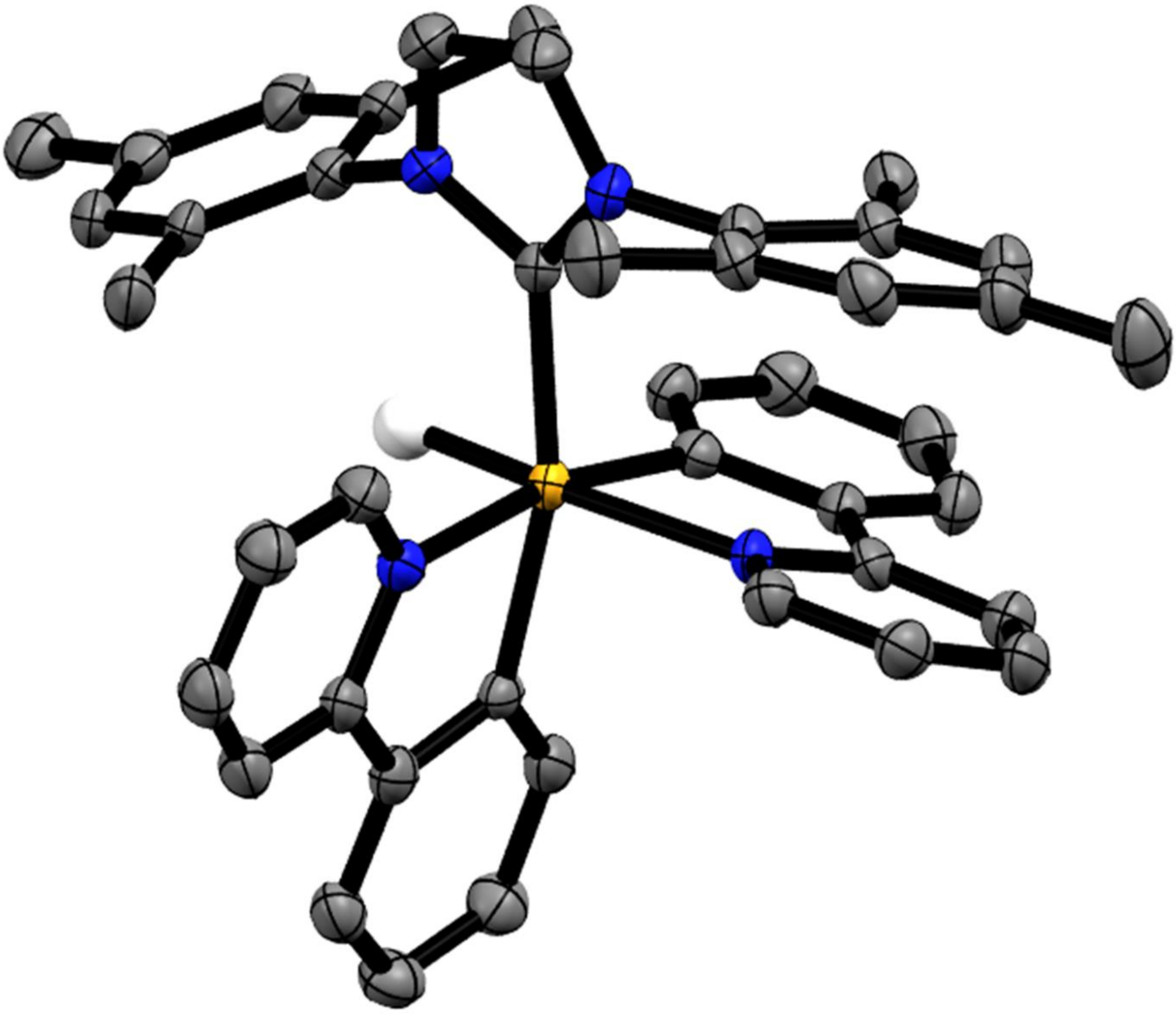

Figure S7: X ray crystal structure of 12. Thermal ellipsoids are shown at 50% probability.

Table S4: X-Ray crystallography details for 12.

Empirical formula	$C_{43}H_{41}IrN_4$
Formula weight/ Da	806.00
Temperature/K	110.00(10)
Crystal system	monoclinic
Space group	$P2_1/c$
a/Å	23.0394(3)
b/Å	16.1288(2)
c/Å	20.7130(2)
α/°	90
β/°	116.322(2)
γ/°	90
Volume/Å^3	6898.86(18)
Z	8
ρ_{calc}g/cm^3	1.552
μ/mm^{-1}	7.768
F(000)	3232.0
Crystal size/mm^3	0.298 × 0.054 × 0.039
Radiation	Cu Kα (λ = 1.54184)
2Θ range for data collection/°	6.954 to 136.498
Index ranges	$-27 \le h \le 27$, $-19 \le k \le 18$, $-20 \le l \le 24$
Reflections collected	50622
Independent reflections	12601 [R_{int} = 0.0333, R_{sigma} = 0.0282]
Data/restraints/parameters	12601/2/885
Goodness-of-fit on F^2	1.040
Final R indexes [I>=2σ (I)]	R_1 = 0.0297, wR_2 = 0.0706
Final R indexes [all data]	R_1 = 0.0359, wR_2 = 0.0736
Largest diff. peak/hole / e Å^{-3}	1.91/-0.93

S3: Synthetic details of Deuterium Labelling Experiments

The deuterium labelling of the substrates was achieved following General Procedure A or General Procedure B (see Experimental Section, main text). The % deuteration values were determined by dividing the ^{1}H NMR signal integral of the site after 24 hours reaction normalized to the integral of an internal standard (grease at δ 0.12) by the corresponding value recorded before the reaction commenced (*i.e.* before H_2 addition) using the equation below:

$$\% \text{ Deuteration} = 100 - [\left(\frac{\text{normalised integral after 24 h reaction}}{\text{normalised integral before } H_2 \text{ addition}}\right) \text{x } 100]$$

NMR data for the starting compounds are provided and are accompanied by details of the deuteration of all substrates as:
(a) Amount of catalyst
(b) Amount of substrate
(c) Amount of base
(d) Volume of solvent
(e) % D in labelled substrate
Corresponding Mass spectrometry data are given in Section S4 and NMR data in Section S5.

***A** (quinoxaline)*

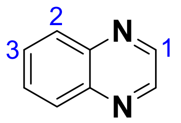

^{1}H NMR (methanol-*d*$_4$, 400 MHz, 298 K) δ 8.92 (s, 2H, H-1), 8.14 (m, 2H, H-2), 7.90 (m, 2H, H-3) ppm.

Following General Procedure A
Condition 1- no base
(a) **1** (1.95 mg, 0.003 mmol, 8.0 mol %), (b) **A** (4.96 mg, 0.038 mmol.), (c) N/A, (d) methanol-*d*$_4$ (0.6 mL), (e) H-1 21 % D, H-2 11 % D, H-3 18 % D.
Condition 2 – with base
(a) **1** (1.95 mg, 0.003 mmol, 8.1 mol %) (b) **A** (4.87 mg, 0.037 mmol), (c) NaOMe (7.2 µL of a 25% w/w solution in MeOH, 0.026 mmol), d) methanol-*d*$_4$ (0.6 mL), (e) H-1 79 % D, H-2 23 % D, H-3 40 % D.

***B** (3,5-dichloropyridine)*

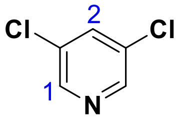

^{1}H NMR (methanol-*d*$_4$, 400 MHz, 298 K) δ 8.50 (d, 2H, $^{4}J_{HH}$ = 2 Hz, H-1), 7.99 (t, 1H, $^{4}J_{HH}$ = 2 Hz, H-2) ppm.

Following General Procedure A
Condition 1- no base
(a) **1** (2.00 mg, 0.003 mmol, 9.4 mol %), (b) **B** (4.87 mg, 0.033 mmol.), (c) N/A, (d) methanol-*d*$_4$ (0.6 mL), (e) H-1 31 % D, H-2 17 % D.
Condition 2 – with base
(a) **1** (2.00 mg, 0.003 mmol, 9.4 mol %), (b) **B** (4.93 mg, 0.037 mmol), (c) NaOMe (7.2 µL of a 25% w/w solution in MeOH, 0.026 mmol), d) methanol-*d*$_4$ (0.6 mL), (e) H-1 79 % D, H-2 14 % D.

***C** (isoquinoline)*

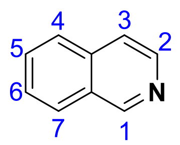

^{1}H NMR (methanol-d_4, 400 MHz, 298 K) δ 9.25 (s, 1H, H-1), 8.44 (d, 1H, $^3J_{HH}$ = 6 Hz, H-2), 8.12 (d, 1H, $^3J_{HH}$ = 8 Hz, H-7), 7.92 (d, 1H, $^3J_{HH}$ = 8 Hz, H-4), 7.87-7.79 (overlap, 2H, H-3, H-5), 7.72 (dt, 2H, $^3J_{HH}$ = 8 Hz, $^4J_{HH}$ = 1 Hz, H-6) ppm.

Following General Procedure A
Condition 1- no base
(a) **1** (1.92 mg, 0.003 mmol, 8.7 mol %), (b) **C** (4.44 mg, 0.034 mmol.), (c) N/A, (d) methanol-d_4 (0.6 mL), (e) H-1 0 % D, H-2 0 % D, H-7 0 % D.
Condition 2 – with base
(a) **1** (1.91 mg, 0.003 mmol, 9.1 mol %), (b) **C** (4.24 mg, 0.033 mmol), (c) NaOMe (7.2 µL of a 25% w/w solution in MeOH, 0.026 mmol), (d) methanol-d_4 (0.6 mL), (e) H-1 92 % D, H-2 90 % D, H-7 22 % D.

***D** (3,5-dibromopyridine)*

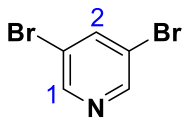

^{1}H NMR (methanol-d_4, 400 MHz, 298 K) δ 8.67 (d, 2H, $^4J_{HH}$ = 2 Hz, H-1), 8.30 (t, 1H, $^4J_{HH}$ = 2 Hz, H-2) ppm.

Following General Procedure A
Condition 1- no base
(a) **1** (1.97 mg, 0.003 mmol, 9.9 mol %), (b) **D** (7.37 mg, 0.031 mmol.), (c) N/A, (d) methanol-d_4 (0.6 mL), (e) H-1 6 % D, H-2 4 % D.
Condition 2 – with base
(a) **1** (1.97 mg, 0.003 mmol, 9.9 mol %), (b) **D** (7.37 mg, 0.031 mmol), (c) NaOMe (7.2 µL of a 25% w/w solution in MeOH, 0.026 mmol), (d) methanol-d_4 (0.6 mL), (e) H-1 84 % D, H-2 8 % D.

***E** (2-phenylpyridine)*

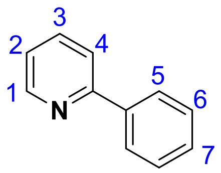

^{1}H NMR (methanol-d_4, 400 MHz, 298 K) δ 8.62 (d, 1H, $^3J_{HH}$ = 5 Hz, H-1), 7.92 (d, 1H, $^3J_{HH}$ = 7 Hz, H-5), 7.88 (overlap, 2H, H-3,4), 7.55-7.43 (overlap 2H, H-6,7), 7.38 (t, 1H, $^3J_{HH}$ = 5 Hz, H-2) ppm.

Following General Procedure A
Condition 1- no base
(a) **1** (1.93 mg, 0.003 mmol, 9.9 mol %), (b) **E** (4.35 µL, 0.030 mmol.), (c) N/A, (d) methanol-d_4 (0.6 mL), (e) H-1 22 % D, H-5 72 % D.
Condition 2 – with base
(a) **1** (1.95 mg, 0.003 mmol, 10.0 mol %), (b) **E** (4.35 µL, 0.030 mmol), (c) NaOMe (7.2 µL of a 25% w/w solution in MeOH, 0.026 mmol), (d) methanol-d_4 (0.6 mL), (e) H-1 31 % D, H-5 74 % D.

***F** (2,5-lutidine)*

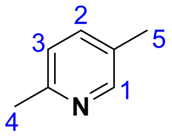

^{1}H NMR (methanol-*d*$_4$, 400 MHz, 298 K) *δ* 8.24 (d, 2H, $^4J_{HH}$ = 2 Hz, H-1), 7.57 (dd, 1H, $^3J_{HH}$ = 8 Hz, $^4J_{HH}$ = 2 Hz, H-2), 7.20 (d, 1H, $^3J_{HH}$ = 8 Hz, H-3), 2.49 (s, 3H, H-4), 2.33 (s, 3H, H-5) ppm.

Following General Procedure A
Condition 1- no base
(a) **1** (2.06 mg, 0.003 mmol, 10.1 mol %), (b) **F** (3.7 µL, 0.032 mmol.), (c) N/A, (d) methanol-*d*$_4$ (0.6 mL), (e) H-1 85 % D, H-4 32 % D.
Condition 2 – with base
(a) **1** (2.08 mg, 0.003 mmol, 10.2 mol %), (b) **F** (3.7 µL, 0.032 mmol), (c) NaOMe (7.2 µL of a 25% w/w solution in MeOH, 0.026 mmol), (d) methanol-*d*$_4$ (0.6 mL), (e) H-1 94 % D, H-4 26 % D.

***G** (nicotine)*

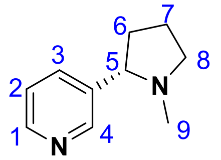

^{1}H NMR (methanol-*d*$_4$, 400 MHz, 298 K) *δ* 8.52 (s, 1H, H-4), 8.46 (d, 1H, $^3J_{HH}$ = 4 Hz H-1), 7.88 (d, 1H, $^3J_{HH}$ = 8 Hz, H-3), 7.45 (m, 1H, H-2), 3.25 (m, 1H), 2.46 (s, 1H), 2.41 (m, 1H), 2.28 (m, 1H), 2.20 (s, 3H, H-9), 2.00 (m, 1H), 1.90 (m, 1H), 1.79 (m, 1H) ppm.

Following General Procedure A
Condition 1- no base
(a) **1** (2.06 mg, 0.003 mmol, 10.1 mol %), (b) **G** (5.14 µL, 0.032 mmol.), (c) N/A, (d) methanol-*d*$_4$ (0.6 mL), (e) H-1 10 % D, H-4 8 % D.
Condition 2 – with base
(a) **1** (2.04 mg, 0.003 mmol, 10.0 mol %), (b) **G** (5.14 µL, 0.032 mmol), (c) NaOMe (7.2 µL of a 25% w/w solution in MeOH, 0.026 mmol), (d) methanol-*d*$_4$ (0.6 mL), (e) H-1 90 % D, H-4 91 % D.

***H** (caffeine)*

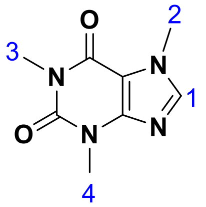

^{1}H NMR (methanol-*d*$_4$, 400 MHz, 298 K) *δ* 7.87 (s, 1H, H-1), 3.98 (s, 3H, H-2), 3.54 (s, 3H, H-4), 3.35 (s, 3H, H-3) ppm.

Following General Procedure A
Condition 1- no base
(a) **1** (2.06 mg, 0.003 mmol, 9.8 mol %), (b) **H** (6.36 mg, 0.033 mmol.), (c) N/A, (d) methanol-*d*$_4$ (0.6 mL), (e) H-1 43 % D.
Condition 2 – with base
(a) **1** (2.00 mg, 0.003 mmol, 9.8 mol %), (b) **H** (6.21 mg, 0.032 mmol), (c) NaOMe (7.2 µL of a 25% w/w solution in MeOH, 0.026 mmol), (d) methanol-*d*$_4$ (0.6 mL), (e) H-1 86 % D.

***I** (nicotinamide)*

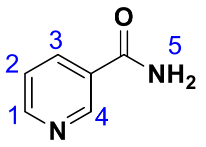

^{1}H NMR (methanol-*d*$_4$, 400 MHz, 298 K) *δ* 9.03 (s, 1H, H-4), 8.70 (m, 1H, H-1), 8.31 (d, 1H, $^3J_{HH}$ = 7.5 Hz, H-3), 7.56 (m, 1H, H-2) ppm.

Following General Procedure A
Condition 1- no base
(a) **1** (1.98 mg, 0.003 mmol, 9.7 mol %), (b) **I** (3.87 mg, 0.032 mmol), (c) N/A, (d) methanol-*d*$_4$ (0.6 mL), (e) H-1 50 % D, H-4 43 % D.
Condition 2 – with base
(a) **1** (1.94 mg, 0.003 mmol, 9.6 mol %), (b) **I** (3.87 mg, 0.032 mmol), (c) NaOMe (7.2 µL of a 25% w/w solution in MeOH, 0.026 mmol), (d) methanol-*d*$_4$ (0.6 mL), (e) H-1 93 % D, H-4 71 % D.

***J** (isoniazid)*

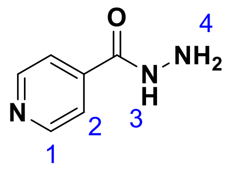

^{1}H NMR (methanol-*d*$_4$, 400 MHz, 298 K) *δ* 8.70 (d, 1H, $^3J_{HH}$ = 5 Hz, H-1), 7.77 (d, 1H, $^3J_{HH}$ = 5 Hz, H-2) ppm.

Following General Procedure A
Condition 1- no base
(a) **1** (2.08 mg, 0.003 mmol, 7.5 mol %), (b) **J** (5.93 mg, 0.043 mmol.), (c) N/A, (d) methanol-*d*$_4$ (0.6 mL), (e) H-1 6 % D, H-2 7 % D.
Condition 2 – with base
(a) **1** (2.08 mg, 0.003 mmol, 7.0 mol %), (b) **J** (6.36 mg, 0.046 mmol), (c) NaOMe (7.2 µL of a 25% w/w solution in MeOH, 0.026 mmol), (d) methanol-*d*$_4$ (0.6 mL), (e) H-1 18 % D, H-2 23 % D.

***K** (anastrozole)*

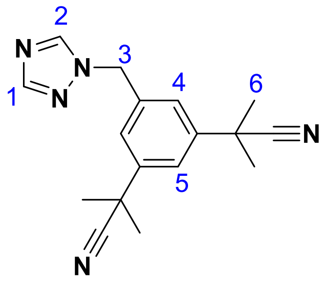

^{1}H NMR (methanol-*d*$_4$, 400 MHz, 298 K) *δ* 8.64 (s, 1H, H-2), 8.05 (s, 1H, H-1), 7.67 (s, 1H, H-5), 7.49 (s, 2H, H-4), 5.54 (s, 2H, H-3), 1.75 (s, 12H, H-6) ppm.

Following General Procedure A
Condition 1- no base
(a) **1** (1.94 mg, 0.003 mmol, 9.7 mol %), (b) **K** (9.12mg, 0.031 mmol.), (c) N/A, (d) methanol-*d*$_4$ (0.6 mL), (e) H-1 8 % D, H-2 12 % D.
Condition 2 – with base
(a) **1** (2.08 mg, 0.003 mmol, 9.7 mol %), (b) **K** (9.86 mg, 0.034 mmol), (c) NaOMe (7.2 µL of a 25% w/w solution in MeOH, 0.026 mmol), (d) methanol-*d*$_4$ (0.6 mL), (e) H-1 84 % D, H-2 57 % D.

***L** (trimethoprim)*

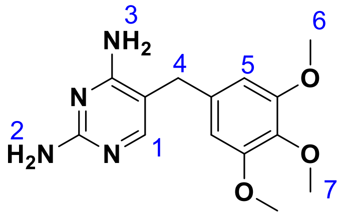

^{1}H NMR (methanol-d_4, 400 MHz, 298 K) δ 7.53 (s, 1H, H-1), 6.54 (s, 2H, H-4), 3.8-3.6 (overlap, 11H, H-4,6,7) ppm.

Following General Procedure A
Condition 1- no base
(a) **1** (2.07 mg, 0.003 mmol, 8.9 mol %), (b) **L** (10.49 mg, 0.036 mmol.), (c) N/A, (d) methanol-d_4 (0.6 mL), (e) H-1 23 % D.
Condition 2 – with base
(a) **1** (2.01 mg, 0.003 mmol, 10.1 mol %), (b) **L** (9.20 mg, 0.032 mmol), (c) NaOMe (7.2 µL of a 25% w/w solution in MeOH, 0.026 mmol), (d) methanol-d_4 (0.6 mL), (e) H-1 92 % D.

***M** (bisacodyl)*

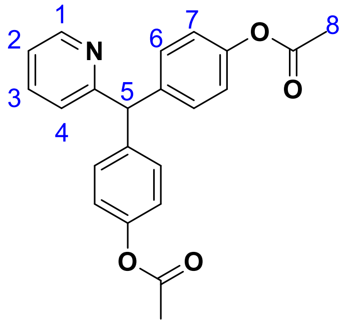

^{1}H NMR (methanol-d_4, 400 MHz, 298 K) δ 8.46 (s, 1H, H-1), 7.76 (s, 1H, H-3), 7.14 (overlap, 2H, H-2,4), 6.91 (s, 2H, H-6), 6.73 (s, 2H, H-7), 2.04 (s, 6H, H-8) ppm.

Following General Procedure A
Condition 1- no base
(a) **1** (2.05 mg, 0.003 mmol, 10 mol %), (b) **M** (54 mg of crushed bisacodyl tablet containing 5% active bisacodyl, 0.03 mmol), (c) N/A, (d) methanol-d_4 (0.6 mL), (e) H-1 60 % D, H-2/H-4 13 % D, H-3 11 % D.
Condition 2 – with base
(a) **1** (1.95 mg, 0.003 mmol, 10 mol %), (b) **M** (54 mg of crushed bisacodyl tablet containing 5% active bisacodyl, 0.03 mmol), (c) NaOMe (7.2 µL of a 25% w/w solution in MeOH, 0.026 mmol), (d) methanol-d_4 (0.6 mL), (e) H-1 95 % D, H-2/H-4 61 % D, H-3 57 % D.

***N** (pyrazine)*

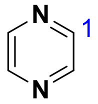

^{1}H NMR (methanol-d_4, 400 MHz, 298 K) δ 8.66 (s, 4H, H-1) ppm.

Following General Procedure A
Condition 1- no base
(a) **1** (2.00 mg, 0.003 mmol, 10 mol %), (b) **N** (2.43 mg, 0.03 mmol.), (c) N/A, (d) methanol-d_4 (0.6 mL), (e) No deuteration observed
Condition 2 – with base
(a) **1** (2.00 mg, 0.003 mmol, 10 mol %), (b) **N** (2.43 mg, 0.03 mmol.), (c) NaOMe (7.2 µL of a 25% w/w solution in MeOH, 0.026 mmol), (d) methanol-d_4 (0.6 mL), (e) No deuteration observed

Following General Procedure B
(a) **1** (2.00 mg, 0.003 mmol, 11.1 mol %), (b) **N** (2.25 mg, 0.028 mmol), (c) NaOMe (7.2 µL of a 25% w/w solution in MeOH, 0.026 mmol), (d) methanol-*d*4 (0.6 mL), (e) H-1 68 % D.

***O** (cytosine)*

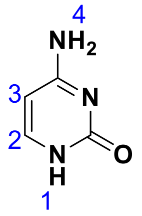

^{1}H NMR (methanol-*d*4, 400 MHz, 298 K) δ 7.44 (d, 1H, $^3J_{HH}$ = 7 Hz, H-2), 5.86 (d, 2H, $^3J_{HH}$ = 7 Hz, H-3) ppm.

Following General Procedure A
Condition 1- no base
(a) **1** (2.00 mg, 0.003 mmol, 10 mol %), (b) **O** (3.33 mg, 0.03 mmol.), (c) N/A, (d) methanol-*d*4 (0.6 mL), (e) No deuteration observed
Condition 2 – with base
(a) **1** (2.00 mg, 0.003 mmol, 10 mol %), (b) **O** (3.33 mg, 0.03 mmol.), (c) NaOMe (7.2 µL of a 25% w/w solution in MeOH, 0.026 mmol), (d) methanol-*d*4 (0.6 mL), (e) No deuteration observed

Following General Procedure B
(a) **1** (2.00 mg, 0.003 mmol, 7.4 mol %), (b) **O** (4.67 mg, 0.042 mmol), (c) NaOMe (7.2 µL of a 25% w/w solution in MeOH, 0.026 mmol), (d) methanol-*d*4 (0.6 mL), (e) H-2 78 % D, H-3 61 % D.

***P** (metronidazole)*

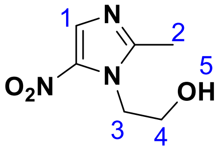

^{1}H NMR (methanol-*d*4, 400 MHz, 298 K) δ 7.96 (s, 1H, H-1), 4.50 (t, 2H, $^3J_{HH}$ = 5 Hz, H-3), 3.89 (t, 2H, $^3J_{HH}$ = 5 Hz, H-4), 2.55 (s, 3H, H-2), ppm.

Following General Procedure A
Condition 1- no base
(a) **1** (2.00 mg, 0.003 mmol, 10 mol %), (b) **P** (5.14 mg, 0.03 mmol.), (c) N/A, (d) methanol-*d*4 (0.6 mL), (e) No deuteration observed
Condition 2 – with base
(a) **1** (2.00 mg, 0.003 mmol, 10 mol %), (b) **P** (5.14 mg, 0.03 mmol.), (c) NaOMe (7.2 µL of a 25% w/w solution in MeOH, 0.026 mmol), (d) methanol-*d*4 (0.6 mL), (e) No deuteration observed

Following General Procedure B
(a) **1** (2.00 mg, 0.003 mmol, 7.9 mol %), (b) **P** (6.78 mg, 0.04 mmol), (c) NaOMe (7.2 µL of a 25% w/w solution in MeOH, 0.026 mmol), (d) methanol-*d*4 (0.6 mL), (e) H-1 91 % D, H-2 72 % D, H-3 75 % D, H-4 60 % D.

***Q** (imidazole)*

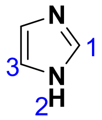

^{1}H NMR (methanol-d_4, 400 MHz, 298 K) δ 7.69 (s 1H, H-1), 7.07 (s, 2H, H-3) ppm.

Following General Procedure A
Condition 1- no base
(a) **1** (2.00 mg, 0.003 mmol, 10 mol %), (b) **Q** (2.04 mg, 0.03 mmol.), (c) N/A, (d) methanol-d_4 (0.6 mL), (e) No deuteration observed
Condition 2 – with base
(a) **1** (2.00 mg, 0.003 mmol, 10 mol %), (b) **Q** (2.03 mg, 0.03 mmol.), (c) NaOMe (7.2 µL of a 25% w/w solution in MeOH, 0.026 mmol), (d) methanol-d_4 (0.6 mL), (e) No deuteration observed

Following General Procedure B
(a) **1** (2.04 mg, 0.003 mmol, 7.2 mol %), (b) **Q** (3.01 mg, 0.044 mmol), (c) NaOMe (7.2 µL of a 25% w/w solution in MeOH, 0.026 mmol), (d) methanol-d_4 (0.6 mL), (e) H-1 92 % D, H-3 74 % D.

S4: Mass Spectrometry of Deuterium Labelling Experiments

For **A**-**M**, each was examined by mass spectrometry for a) a reference sample of the untreated compound in methanol b) a solution of each (50 mM) with [IrCl(COD)(IMes)] (5 mM) in methanol-d_4 after it has reacted with H_2 (3 bar) for 24 hours and c) a solution of each (50 mM) with [IrCl(COD)(IMes)] (5 mM) and NaOMe (50 mM) in methanol-d_4 after it has reacted with H_2 (3 bar) for 24 hours. In both b) and c) the solutions were diluted roughly 1 in 10 with methanol to produce appropriate concentrations for mass spectrometry.
O and **P** were examined by mass spectrometry for a) a reference sample of the untreated compound in methanol b) a solution of each (50 mM) after it has reacted for 24 hours at room temperature with H_2 (3 bar), NaOMe (50 mM) and preformed [Ir(H)$_3$(COD)(IMes)] in methanol-d_4. Note that **4** was preformed by prior reaction of [IrCl(COD)(IMes)] (5 mM) with NaOMe (50 mM) and H_2 (3 bar) in methanol-d_4 for 18 hours at 253 K.
N and **Q** could not be examined by mass spectrometry as their masses are too low to reliably detect using our mass spectrometer, which requires masses above *ca* 100 Da.

S4.1: Mass spectrometry of A

MS (ESI) m/z: [**A** + H]$^+$ Calcd. for $C_8H_7N_2$ 131.1, Found 130.8; [**A-*d*** + H]$^+$ Calcd. for $C_8H_6DN_2$ 132.1, Found 131.7; [**A-*d*$_2$** + H]$^+$ Calcd. for $C_8H_5D_2N_2$ 133.1, Found 132.9; [**A-*d*$_3$** + H]$^+$ Calcd. for $C_8H_4D_3N_2$ 134.1, Found 133.9; [**A-*d*$_4$** + H]$^+$ Calcd. for $C_8H_3D_4N_2$ 135.1, Found 134.9; [**A-*d*$_5$** + H]$^+$ Calcd. for $C_8H_2D_5N_2$ 136.1, Found 135.9; [**A-*d*$_6$** + H]$^+$ Calcd. for $C_8HD_6N_2$ 137.1, Found 136.9.

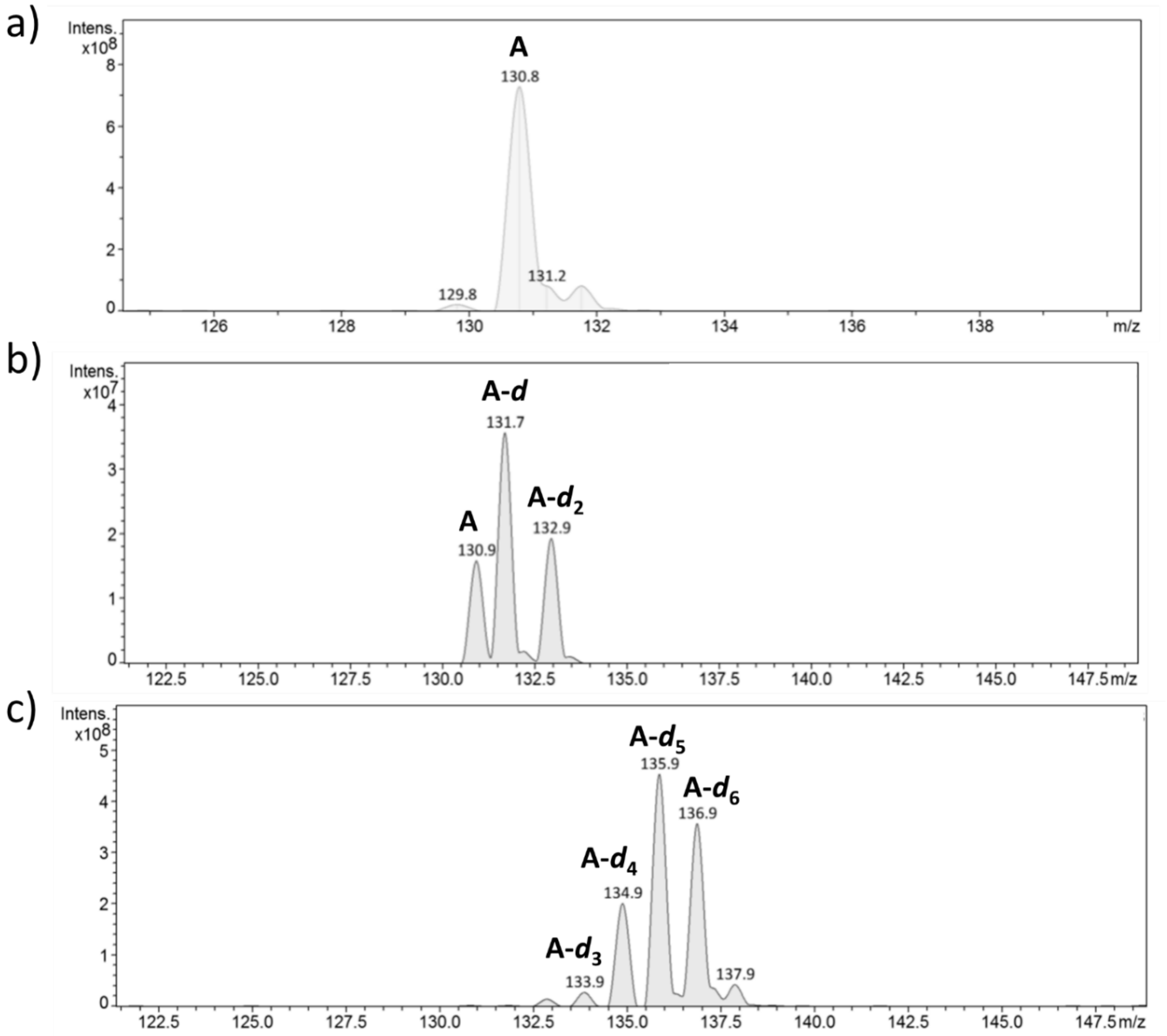

Figure S8: Mass spectra of a) A b) A after its reaction (50 mM) with [IrCl(COD)(IMes)] (5 mM) in methanol-d_4 and H_2 (3 bar) for 24 hours and c) A after its reaction (50 mM) with [IrCl(COD)(IMes)] (5 mM) and NaOMe (50 mM) in methanol-d_4 and H_2 (3 bar) for 24 hours.

S4.2: Mass spectrometry of B

MS (ESI) m/z: [**B**]$^+$ Calcd. for $C_5H_3Cl_2N$ 147.0, Found 146.8; [**B-*d***]$^+$ Calcd. for $C_5H_2DCl_2N$ 148.0, Found 147.8; [**B-*d*₂**]$^+$ Calcd. for $C_5HD_2Cl_2N$ 149.0, Found 148.7.

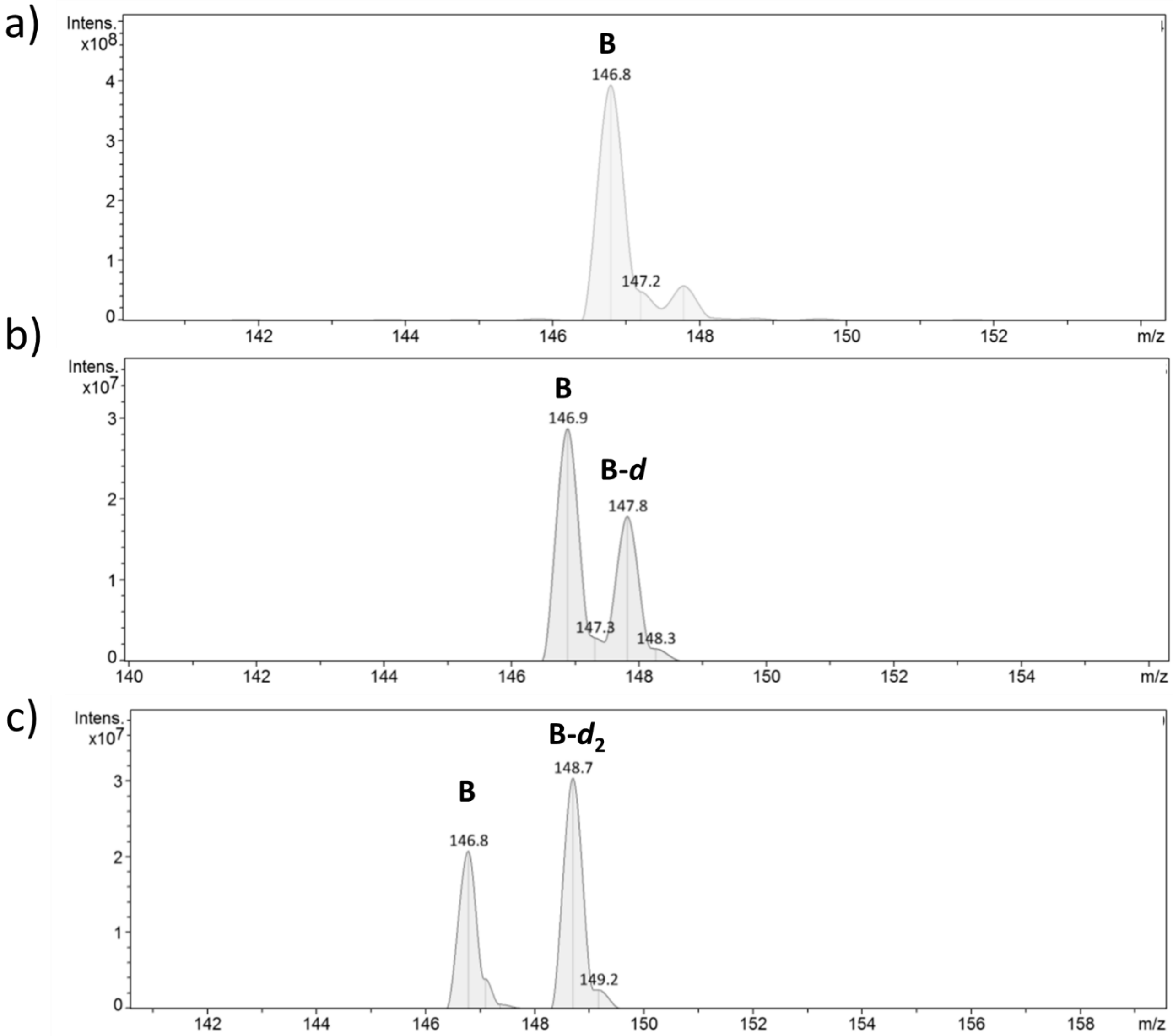

Figure S9: Mass spectra of a) B b) B after its reaction (50 mM) with [IrCl(COD)(IMes)] (5 mM) in methanol-d_4 and H_2 (3 bar) for 24 hours and c) B after its reaction (50 mM) with [IrCl(COD)(IMes)] (5 mM) and NaOMe (50 mM) in methanol-d_4 and H_2 (3 bar) for 24 hours.

S4.3: Mass spectrometry of **C**

MS (ESI) m/z: [**C** + H]$^+$ Calcd. for C_9H_8N 130.1, Found 129.8; [**C**-d_1 + H]$^+$ Calcd. for C_9H_7DN 131.1, Found 130.8; [**C**-d_2 + H]$^+$ Calcd. for $C_9H_6D_2N$ 132.1, Found 131.8; [**C**-d_3 + H]$^+$ Calcd. for $C_9H_5D_3N$ 133.1, Found 132.8.

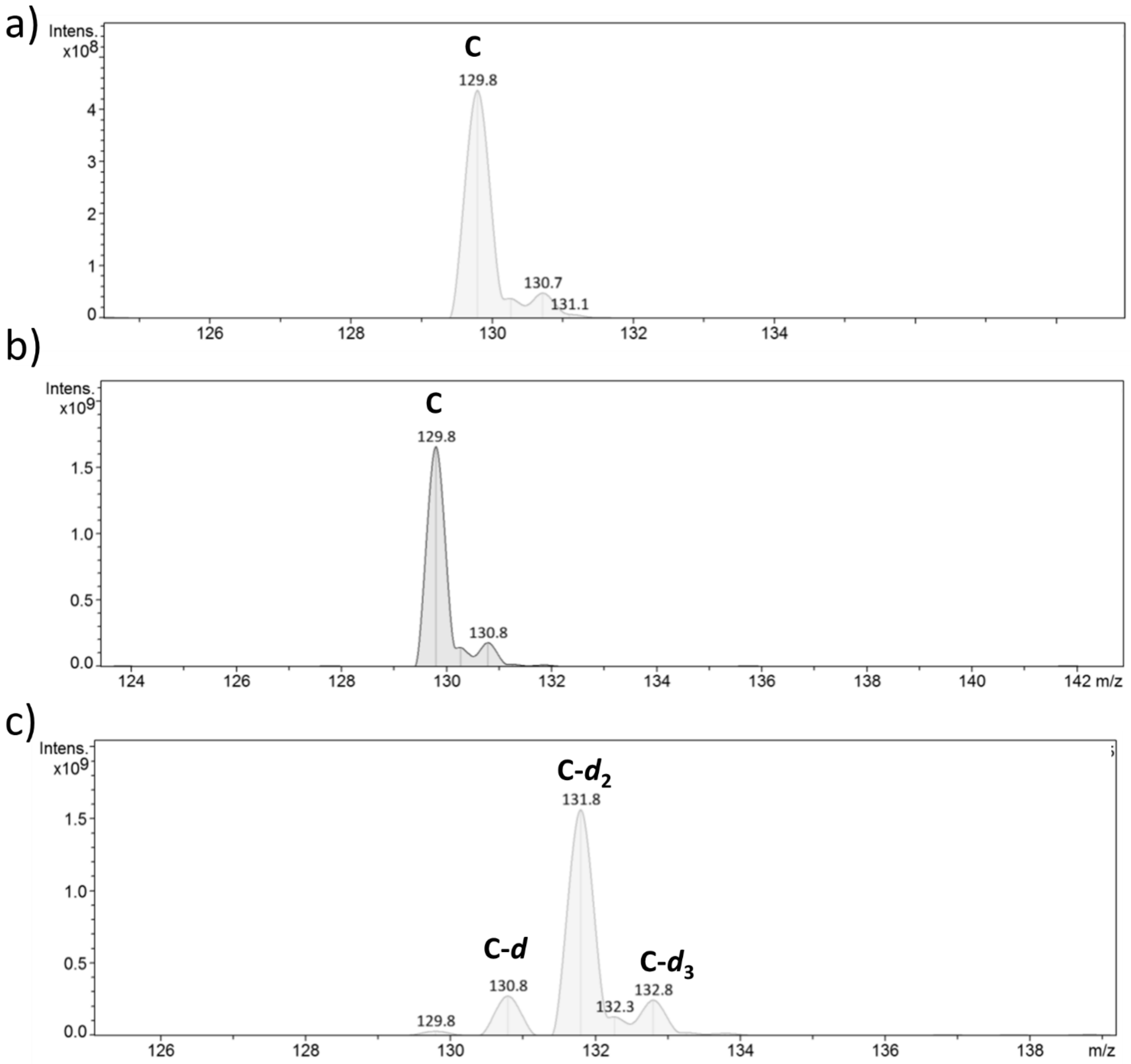

***Figure S10:** Mass spectra of a) C b) C after its reaction (50 mM) with [IrCl(COD)(IMes)] (5 mM) in methanol-d_4 and H_2 (3 bar) for 24 hours and c) C after its reaction (50 mM) with [IrCl(COD)(IMes)] (5 mM) and NaOMe (50 mM) in methanol-d_4 and H_2 (3 bar) for 24 hours.*

S4.4: Mass spectrometry of D

MS (ESI) m/z: [**D** + H]$^+$ Calcd. for $C_5H_4{}^{79}Br_2N$ 235.9, Found 235.6; Calcd. for $C_5H_4{}^{79}Br^{81}BrN$ 237.9, Found 237.6; Calcd. for $C_5H_4{}^{81}Br_2N$ 239.9, Found 239.6; [**D**-d_1 + H]$^+$ Calcd. for $C_5H_3D^{79}Br^{81}BrN$ 238.9, Found 238.7; Calcd. for $C_5H_3D^{81}Br_2N$ 240.9, Found 240.6; [**D**-d_2 + H]$^+$ Calcd. for $C_5H_2D_2{}^{79}Br_2N$ 237.9, Found 237.8; Calcd. for $C_5H_3D^{79}Br^{81}BrN$ 239.8, Found 239.9; Calcd. for $C_5H_3D^{81}Br_2N$ 241.8, Found 241.7.

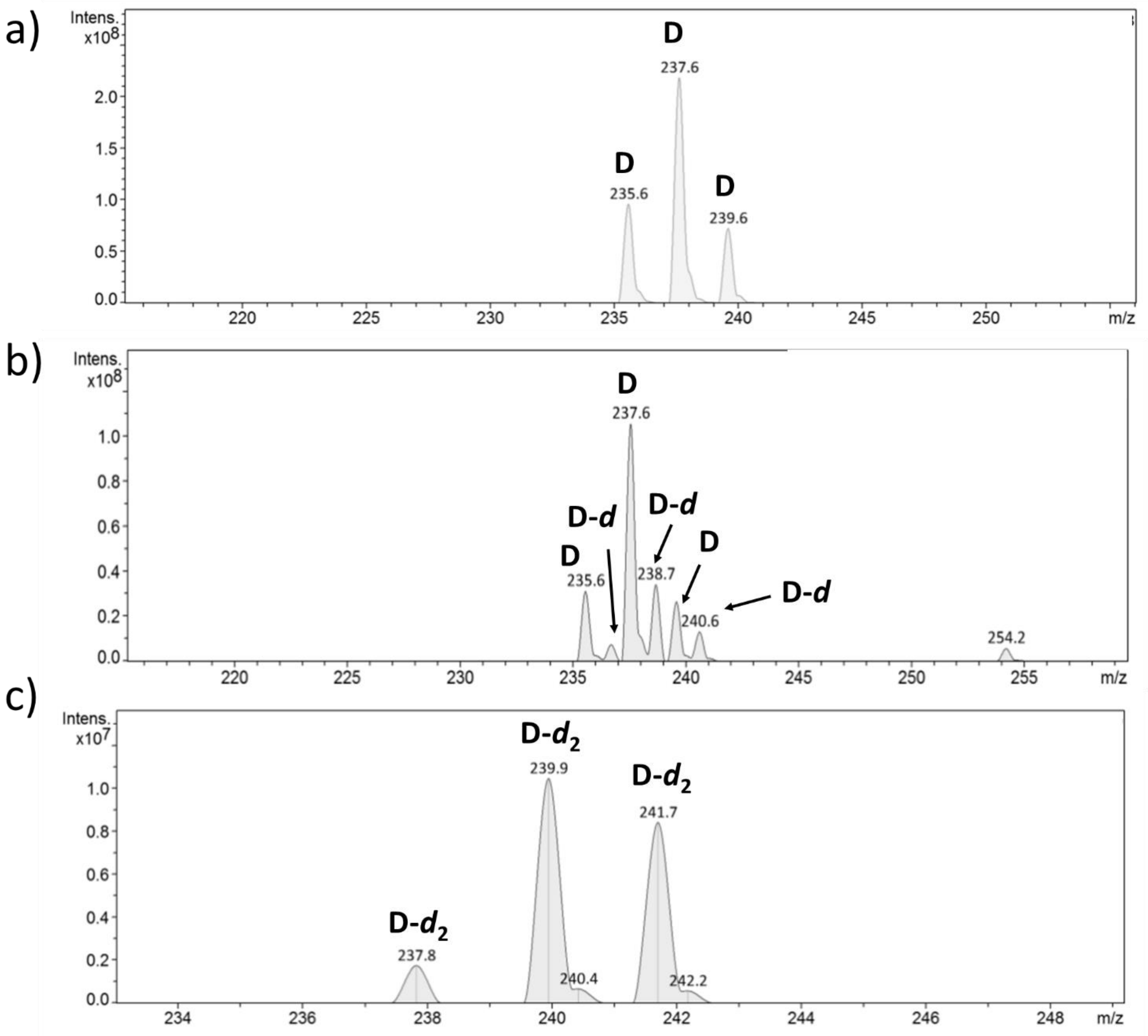

Figure S11: Mass spectra of a) D b) D after its reaction (50 mM) with [IrCl(COD)(IMes)] (5 mM) in methanol-d_4 and H_2 (3 bar) for 24 hours and c) D after its reaction (50 mM) with [IrCl(COD)(IMes)] (5 mM) and NaOMe (50 mM) in methanol-d_4 and H_2 (3 bar) for 24 hours.

S4.5: Mass spectrometry of **E**

MS (ESI) m/z: [**E** + H]$^+$ Calcd. for $C_{11}H_{10}N$ 156.1, Found 155.8; [**E**-d_1 + H]$^+$ Calcd. for $C_{11}H_9DN$ 157.1, Found 156.9; [**E**-d_2 + H]$^+$ Calcd. for $C_{11}H_8D_2N$ 158.1, Found 157.9.

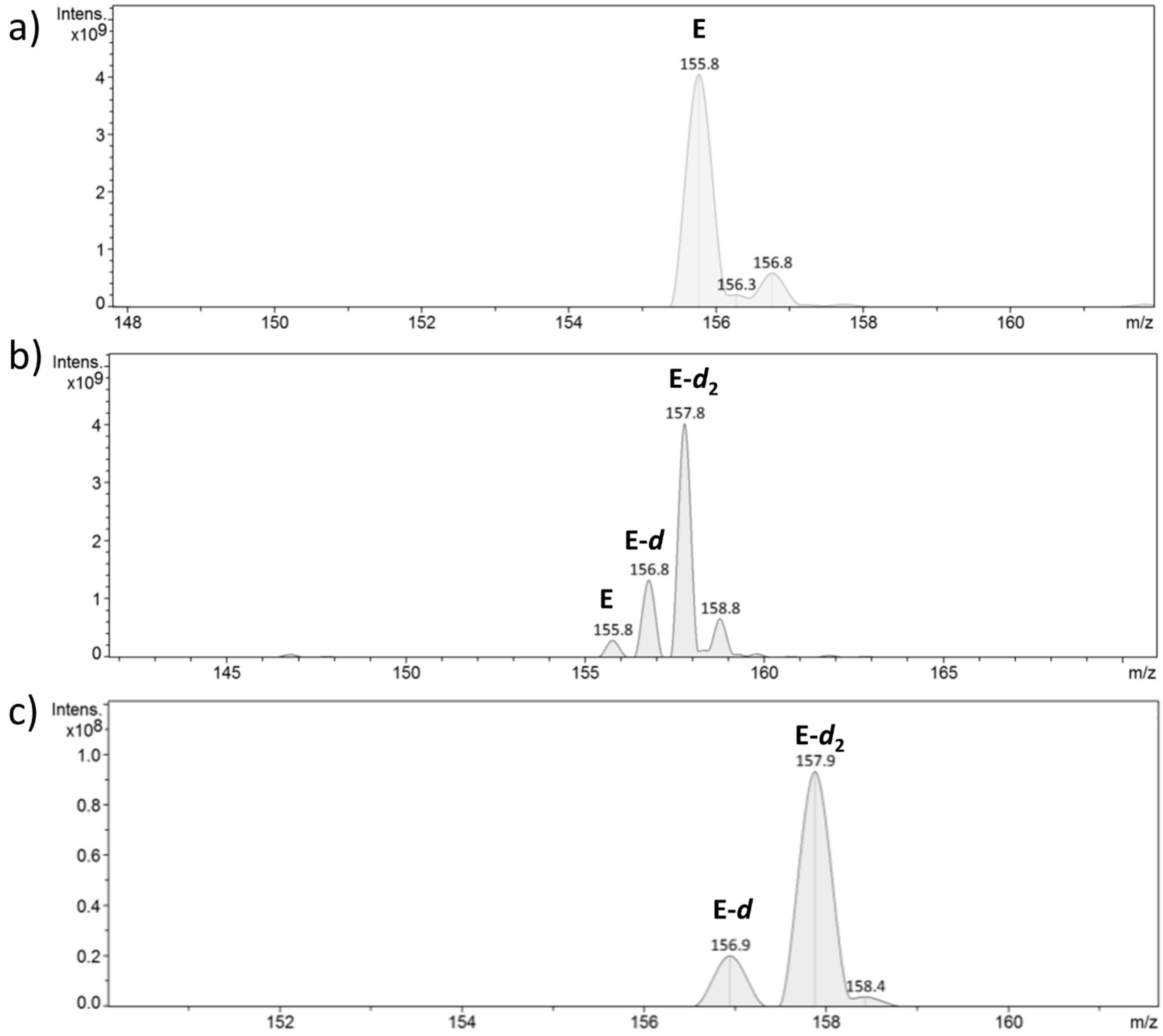

Figure S12: Mass spectra of a) E b) E after its reaction (50 mM) with [IrCl(COD)(IMes)] (5 mM) in methanol-d_4 and H_2 (3 bar) for 24 hours and c) E after its reaction (50 mM) with [IrCl(COD)(IMes)] (5 mM) and NaOMe (50 mM) in methanol-d_4 and H_2 (3 bar) for 24 hours.

S4.6: Mass spectrometry of F

MS (ESI) m/z: [**F** + H]$^+$ Calcd. for $C_7H_{10}N$ 108.1, Found 107.9; [**F**-d_1 + H]$^+$ Calcd. for C_7H_9DN 109.1, Found 108.9; [**F**-d_2 + H]$^+$ Calcd. for $C_7H_8D_2N$ 110.1, Found 109.9; [**F**-d_3 + H]$^+$ Calcd. for $C_7H_7D_3N$ 111.1, Found 110.9; [**F**-d_4 + H]$^+$ Calcd. for $C_7H_6D_4N$ 112.1, Found 111.9.

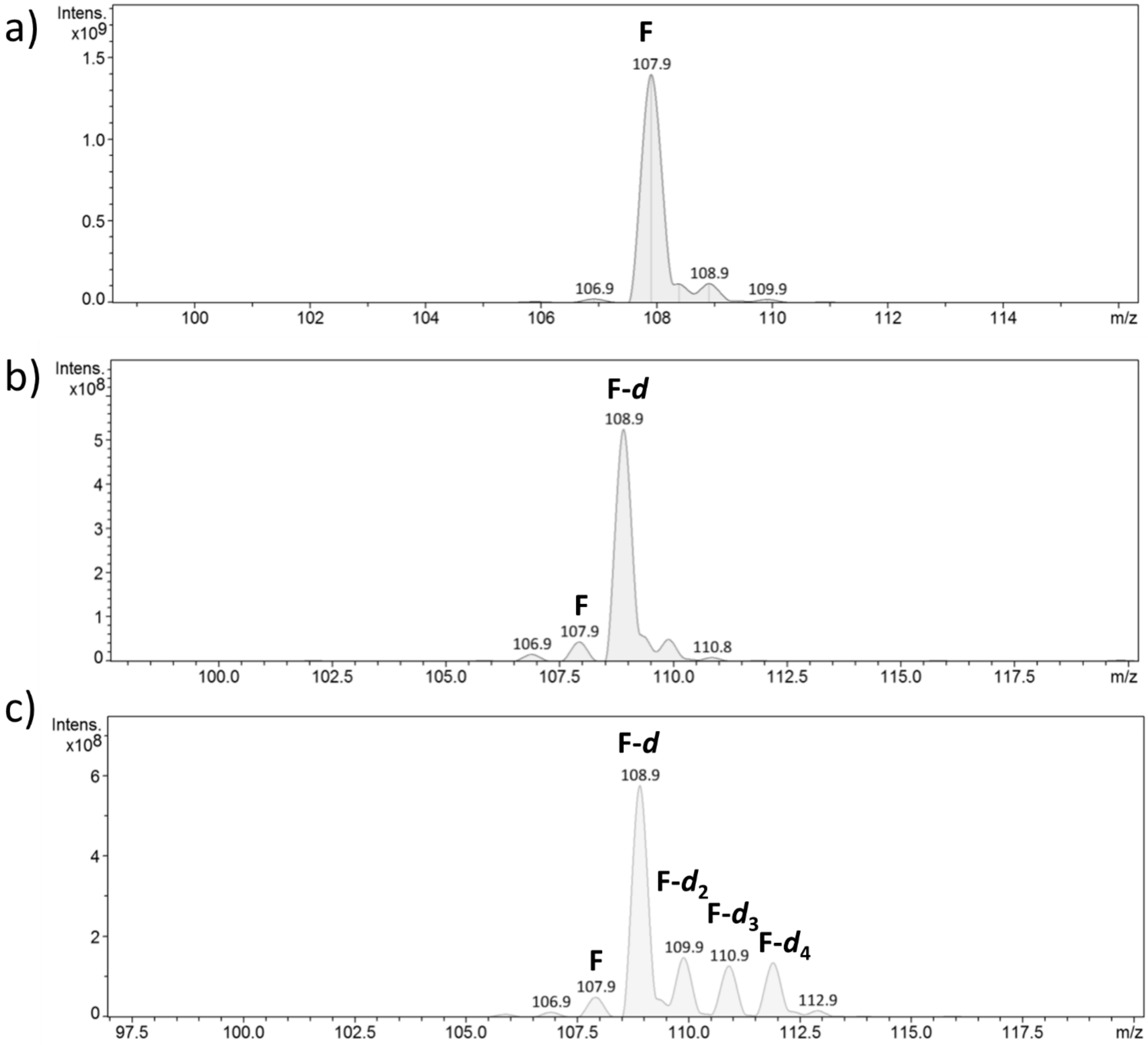

Figure S13: Mass spectra of a) F b) F after its reaction (50 mM) with [IrCl(COD)(IMes)] (5 mM) in methanol-d_4 and H_2 (3 bar) for 24 hours and c) F after its reaction (50 mM) with [IrCl(COD)(IMes)] (5 mM) and NaOMe (50 mM) in methanol-d_4 and H_2 (3 bar) for 24 hours.

S4.7: Mass spectrometry of G

MS (ESI) m/z: [**G** + H]$^+$ Calcd. for $C_{10}H_{15}N_2$ 163.1, Found 162.8; [**G**-d_1 + H]$^+$ Calcd. for $C_{10}H_{14}DN_2$ 164.1, Found 163.8; [**G**-d_2 + H]$^+$ Calcd. for $C_{10}H_{13}D_2N_2$ 165.1, Found 164.8.

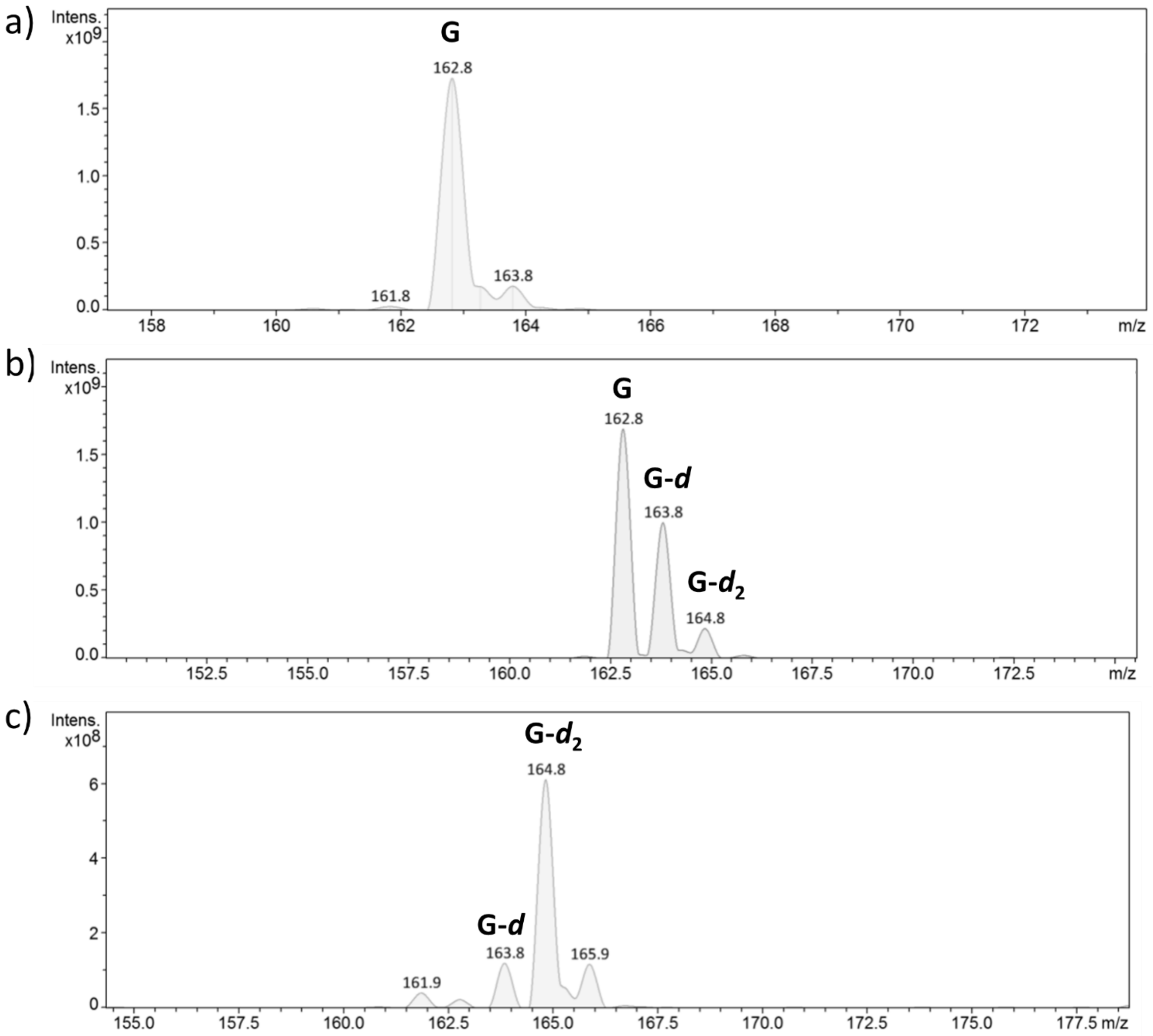

Figure S14: Mass spectra of a) G b) G after its reaction (50 mM) with [IrCl(COD)(IMes)] (5 mM) in methanol-d_4 and H_2 (3 bar) for 24 hours and c) G after its reaction (50 mM) with [IrCl(COD)(IMes)] (5 mM) and NaOMe (50 mM) in methanol-d_4 and H_2 (3 bar) for 24 hours.

S4.8: Mass spectrometry of H

MS (ESI) m/z: [**H** + H]$^+$ Calcd. for $C_8H_{11}N_4O_2$ 195.1, Found 194.8; [**H**-d_1 + H]$^+$ Calcd. for $C_8H_{10}DN_4O_2$ 196.1, Found 195.8.

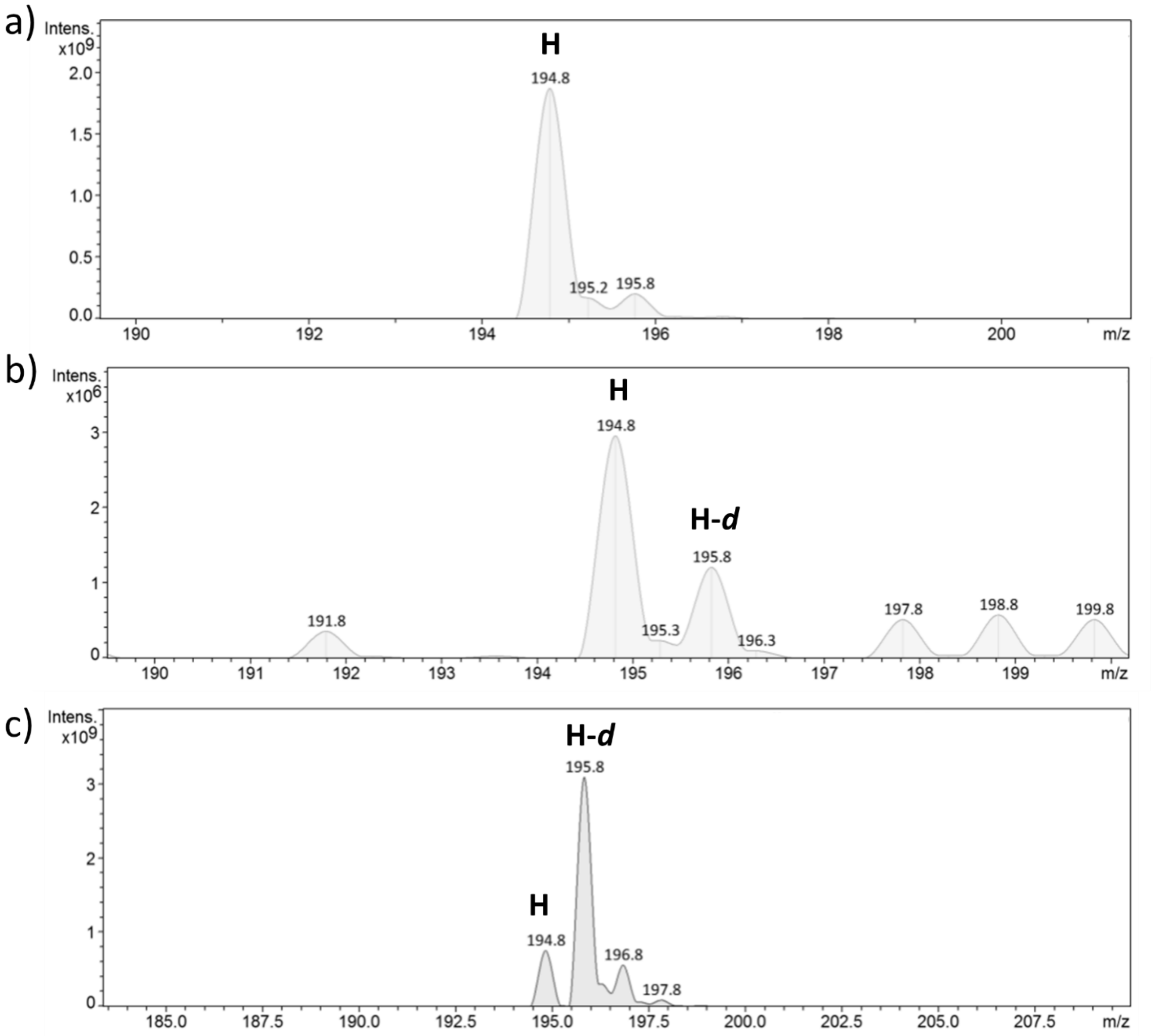

Figure S15: Mass spectra of a) H b) H after its reaction (50 mM) with [IrCl(COD)(IMes)] (5 mM) in methanol-d_4 and H_2 (3 bar) for 24 hours and c) H after its reaction (50 mM) with [IrCl(COD)(IMes)] (5 mM) and NaOMe (50 mM) in methanol-d_4 and H_2 (3 bar) for 24 hours.

S4.9: Mass spectrometry of I

MS (ESI) m/z: [**I** + H]$^+$ Calcd. for $C_6H_7N_2O$ 123.1, Found 122.8; [**I**-d_1 + H]$^+$ Calcd. for $C_6H_6DN_2O$ 124.1, Found 123.8; [**I**-d_2 + H]$^+$ Calcd. for $C_6H_5D_2N_2O$ 125.1, Found 124.8.

Mass Spectral analysis of **I** was complicated by the presence of a contaminant species on the column of our LCMS with m/z at 121.8/121.9, which is similar to that of **I** at 122.8.

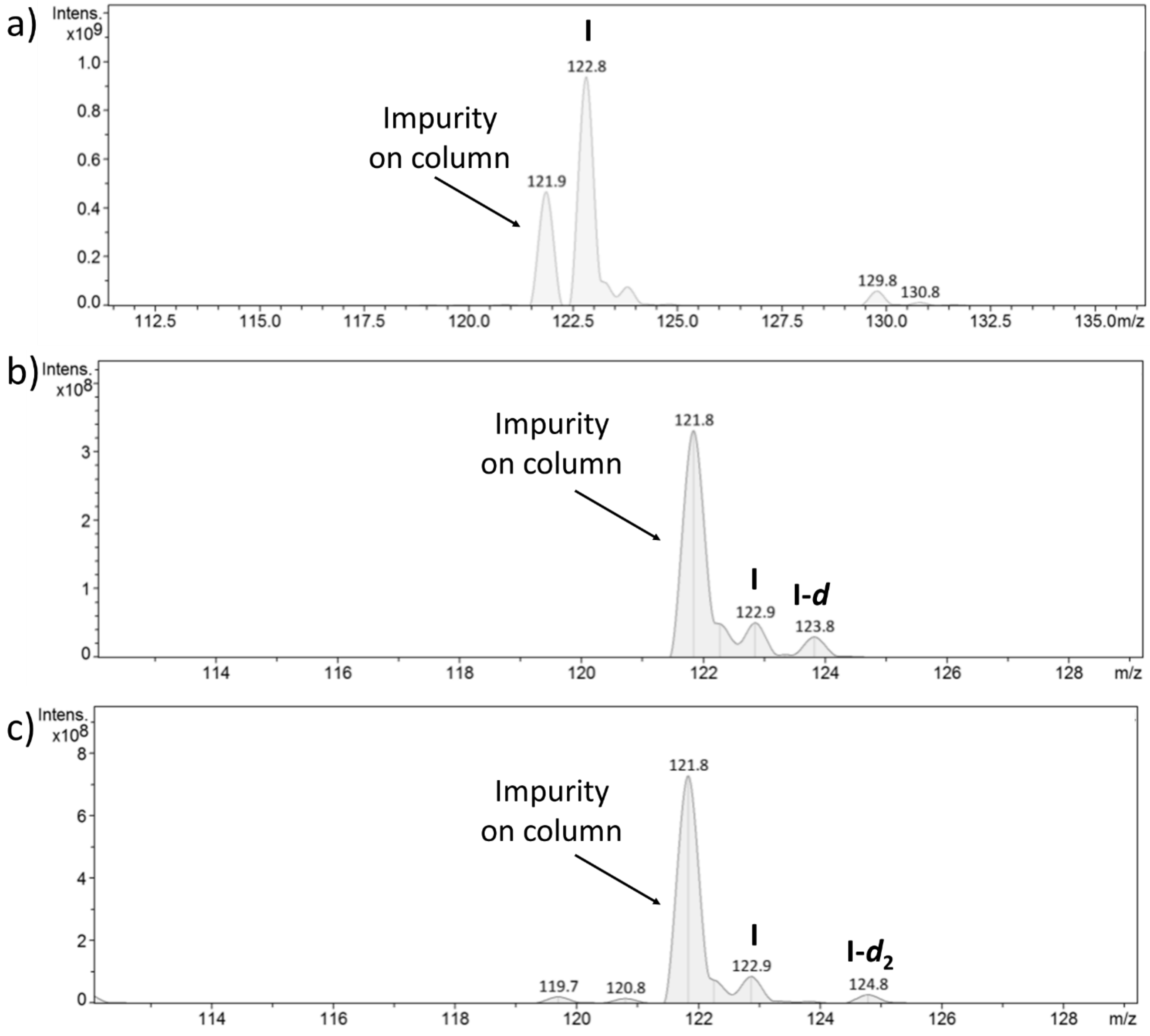

Figure S16: Mass spectra of a) I b) I after its reaction (50 mM) with [IrCl(COD)(IMes)] (5 mM) in methanol-d_4 and H_2 (3 bar) for 24 hours and c) I after its reaction (50 mM) with [IrCl(COD)(IMes)] (5 mM) and NaOMe (50 mM) in methanol-d_4 and H_2 (3 bar) for 24 hours.

S4.10: Mass spectrometry of J

MS (ESI) m/z: [**J** + H]$^+$ Calcd. for $C_6H_8N_3O$ 138.1, Found 137.9; [**J**-d_1 + H]$^+$ Calcd. for $C_6H_7DN_3O$ 139.1, Found 138.8; [**J**-d_2 + H]$^+$ Calcd. for $C_6H_6D_2N_3O$ 140.1, Found 139.8; [**J**-d_3 + H]$^+$ Calcd. for $C_6H_5D_3N_3O$ 141.1, Found 140.8.

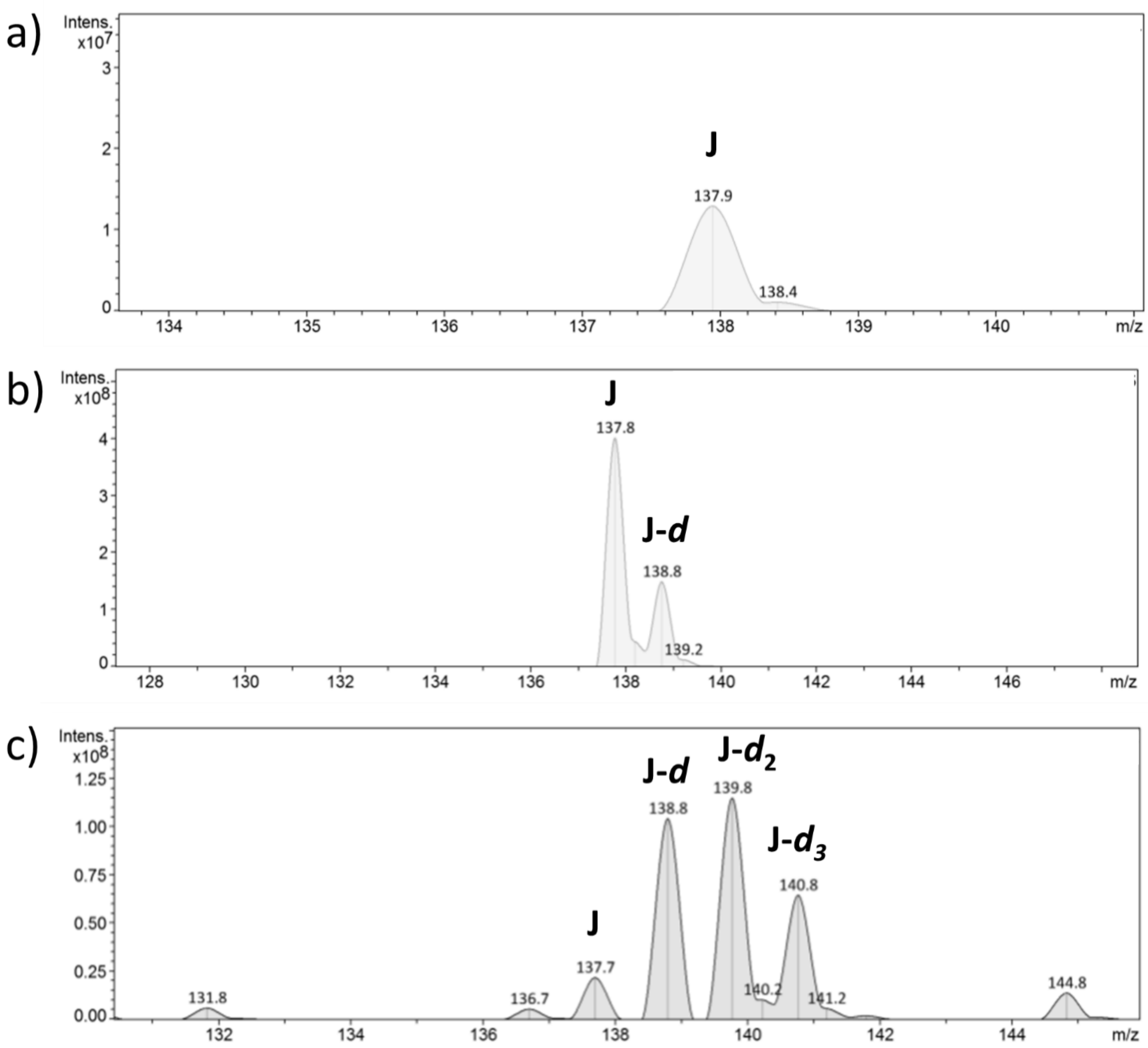

Figure S17: Mass spectra of a) J b) J after its reaction (50 mM) with [IrCl(COD)(IMes)] (5 mM) in methanol-d$_4$ and H$_2$ (3 bar) for 24 hours and c) J after its reaction (50 mM) with [IrCl(COD)(IMes)] (5 mM) and NaOMe (50 mM) in methanol-d$_4$ and H$_2$ (3 bar) for 24 hours.

S4.11: Mass spectrometry of K

MS (ESI) m/z: [**K** + H]$^+$ Calcd. for $C_{17}H_{20}N_5$ 294.2, Found 294.0; [**K**-d_1 + H]$^+$ Calcd. for $C_{17}H_{19}DN_5$ 295.2, Found 295.0; [**K**-d_2 + H]$^+$ Calcd. for $C_{17}H_{18}D_2N_5$ 296.2, Found 296.1.

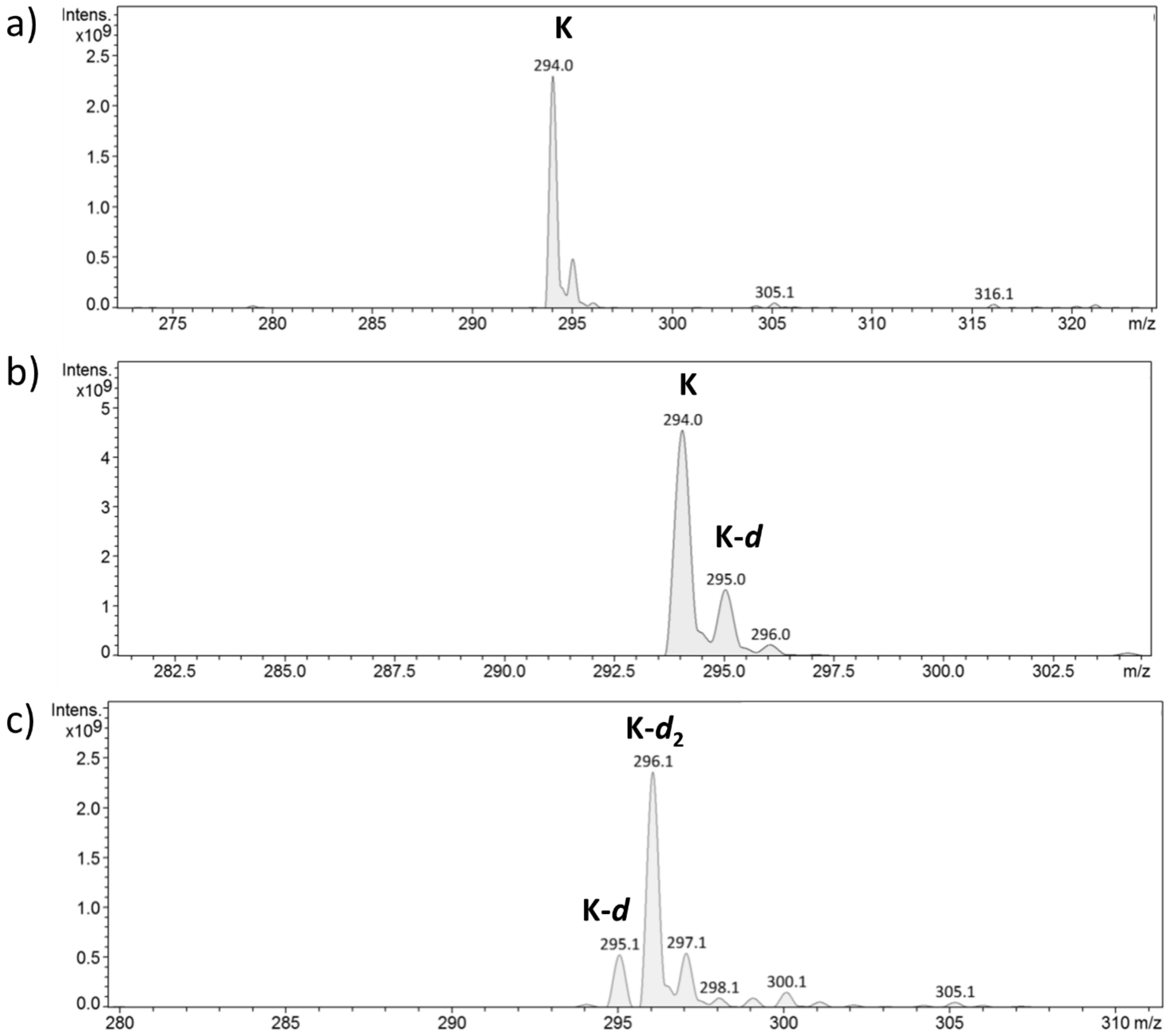

Figure S18: Mass spectra of a) K b) K after its reaction (50 mM) with [IrCl(COD)(IMes)] (5 mM) in methanol-d_4 and H_2 (3 bar) for 24 hours and c) K after its reaction (50 mM) with [IrCl(COD)(IMes)] (5 mM) and NaOMe (50 mM) in methanol-d_4 and H_2 (3 bar) for 24 hours.

S4.12: Mass spectrometry of **L**

MS (ESI) m/z: [**L** + H]+ Calcd. for $C_{14}H_{19}N_4O_3$ 291.1, Found 291.0; [**L**-d_1 + H]+ Calcd. for $C_{14}H_{18}DN_4O_3$ 292.1, Found 292.1.

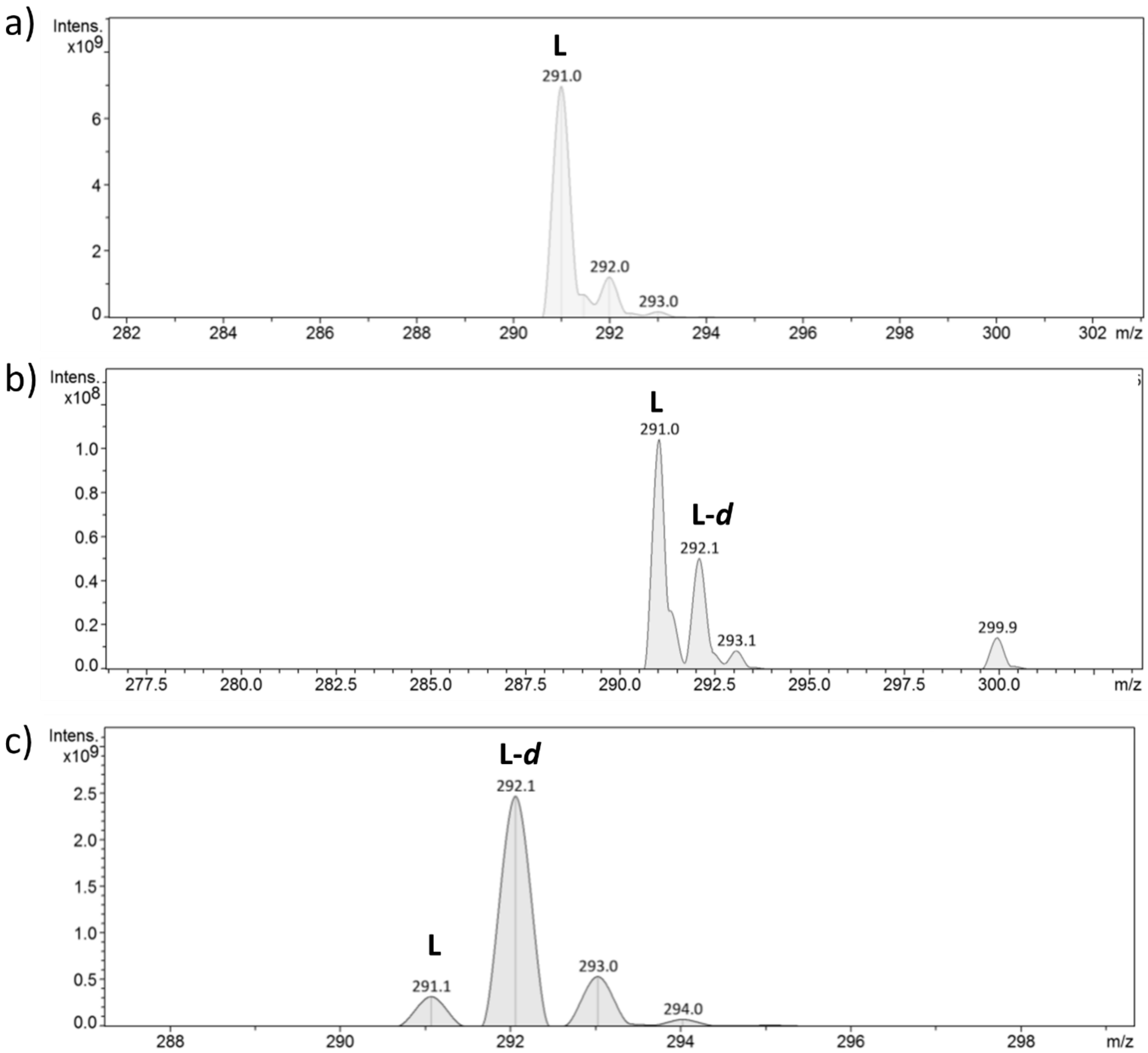

Figure S19: Mass spectra of a) L b) L after its reaction (50 mM) with [IrCl(COD)(IMes)] (5 mM) in methanol-d_4 and H_2 (3 bar) for 24 hours and c) L after its reaction (50 mM) with [IrCl(COD)(IMes)] (5 mM) and NaOMe (50 mM) in methanol-d_4 and H_2 (3 bar) for 24 hours.

S4.13: Mass spectrometry of M

MS (ESI) m/z: [**M** + H]$^+$ Calcd. for $C_{22}H_{20}NO_4$ 362.1, Found 362.1; [**M**-d_1 + H]$^+$ Calcd. for $C_{22}H_{19}DNO_4$ 363.1, Found 363.1; [**M**-d_2 + H]$^+$ Calcd. for $C_{22}H_{18}D_2NO_4$ 364.1, Found 364.1; [**M**-d_3 + H]$^+$ Calcd. for $C_{22}H_{17}D_3NO_4$ 365.1, Found 365.1; [**M**-d_4 + H]$^+$ Calcd. for $C_{22}H_{16}D_4NO_4$ 366.1, Found 366.1.

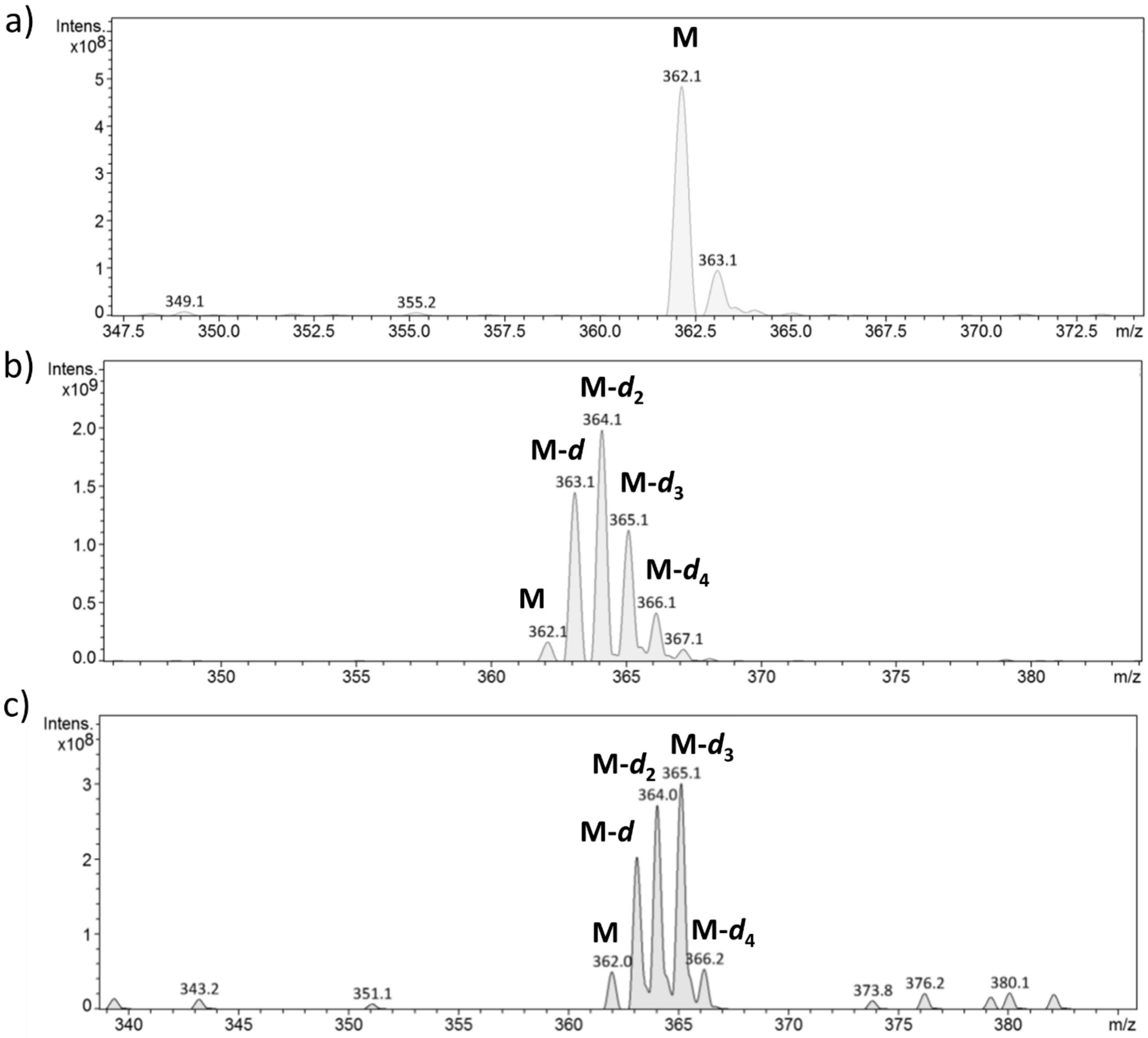

***Figure S20:** Mass spectra of a) M b) M after its reaction (50 mM) with [IrCl(COD)(IMes)] (5 mM) in methanol-d_4 and H_2 (3 bar) for 24 hours and c) M after its reaction (50 mM) with [IrCl(COD)(IMes)] (5 mM) and NaOMe (50 mM) in methanol-d_4 and H_2 (3 bar) for 24 hours.*

S4.14: Mass spectrometry of O

MS (ESI) m/z: [**O** + H]$^+$ Calcd. for $C_4H_6N_3O$ 112.1, Found 111.9; [**O**-d_1 + H]$^+$ Calcd. for $C_4H_5DN_3O$ 113.1, Found 112.9; [**O**-d_2 + H]$^+$ Calcd. for $C_4H_4D_2N_3O$ 114.1, Found 113.9.

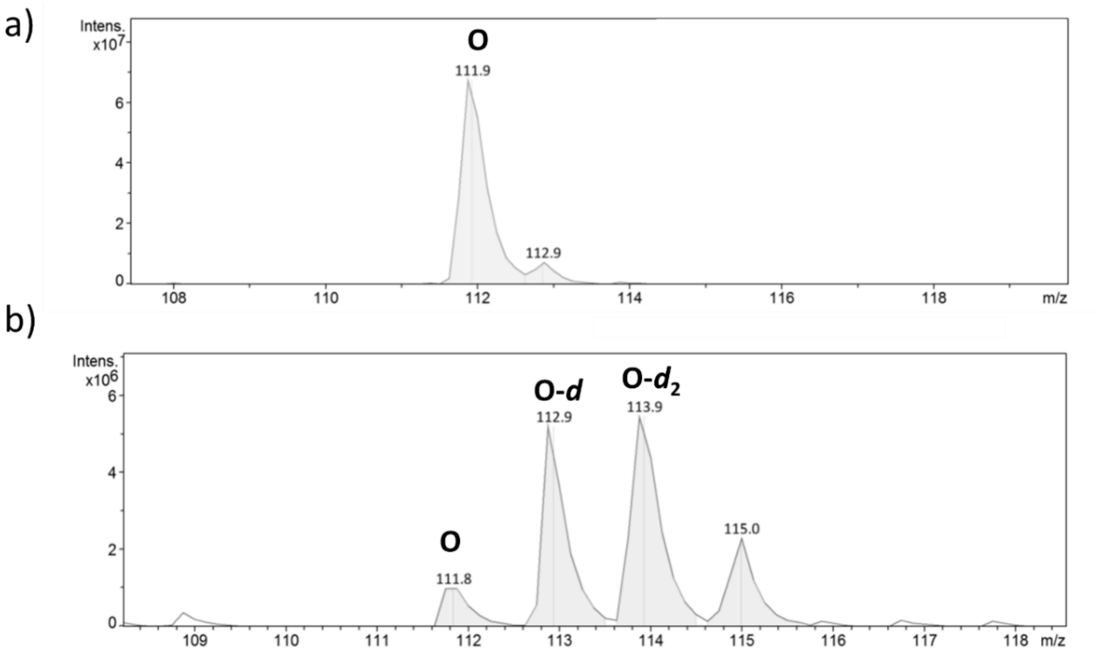

Figure S21: Mass spectra of a) O b) O after its reaction (50 mM) for 24 hours at room temperature with H_2 (3 bar), NaOMe (50 mM) and preformed [Ir(H)$_3$(COD)(IMes)] in methanol-d_4. Note that 4 was preformed by prior reaction of [IrCl(COD)(IMes)] (5 mM) with NaOMe (50 mM) and H_2 (3 bar) in methanol-d_4 for 18 hours at 253 K.

S4.15: Mass spectrometry of P

MS (ESI) m/z: [**P** + H]$^+$ Calcd. for $C_6H_{10}N_3O_3$ 172.1, Found 171.8; [**P**-d_1 + H]$^+$ Calcd. for $C_6H_9DN_3O_3$ 173.1, Found 172.9; [**P**-d_2 + H]$^+$ Calcd. for $C_6H_8D_2N_3O_3$ 174.1, Found 173.8; [**P**-d_3 + H]$^+$ Calcd. for $C_6H_7D_3N_3O_3$ 175.1, Found 174.8; [**P**-d_4 + H]$^+$ Calcd. for $C_6H_6D_4N_3O_3$ 176.1, Found 175.8; [**P**-d_5 + H]$^+$ Calcd. for $C_6H_5D_5N_3O_3$ 177.1, Found 176.9; [**P**-d_7 + H]$^+$ Calcd. for $C_6H_3D_7N_3O_3$ 179.1, Found 179.0.

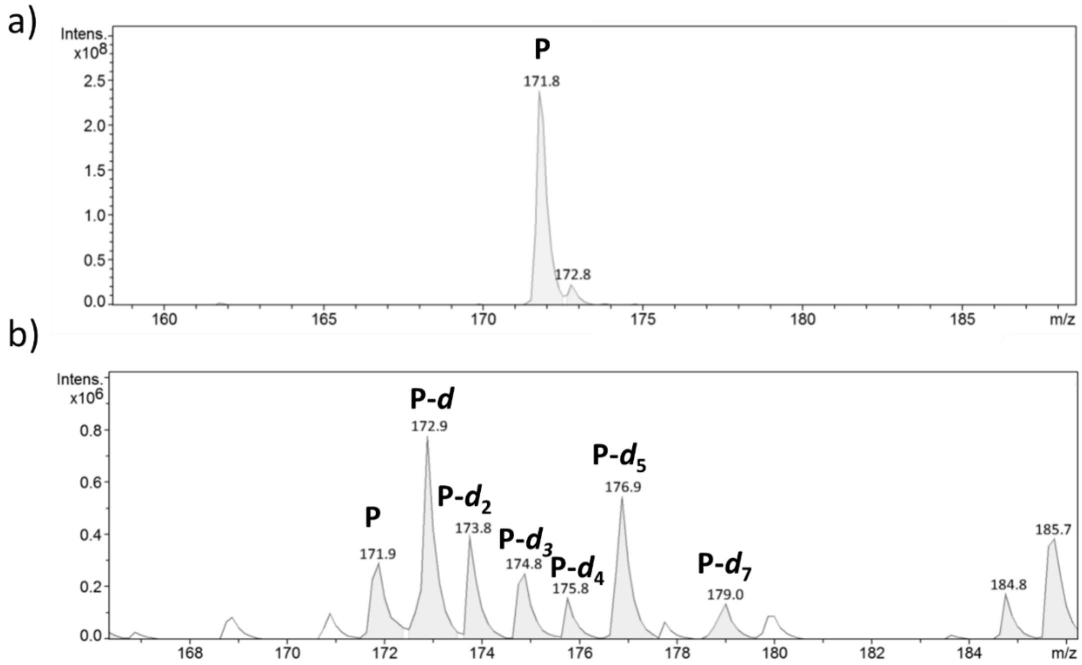

Figure S22: Mass spectra of a) P b) P after its reaction (50 mM) for 24 hours at room temperature with H_2 (3 bar), NaOMe (50 mM) and preformed [Ir(H)$_3$(COD)(IMes)] in methanol-d_4. Note that 4 was preformed by prior reaction of [IrCl(COD)(IMes)] (5 mM) with NaOMe (50 mM) and H_2 (3 bar) in methanol-d_4 for 18 hours at 253 K.

S5: ^{1}H NMR spectroscopy of Deuterium Labelling Experiments

For **A**-**M**, ^{1}H NMR spectra (400 MHz, 298 K) were collected for solutions containing the target compound (50 mM) with [IrCl(COD)(IMes)] (5 mM) in methanol-d_4 before it has reacted with H_2 and after it has reacted with H_2 (3 bar) for 24 hours at room temperature. This was repeated with the addition of NaOMe (50 mM). For **O**-**Q** which were deuterated using General Approach 2, ^{1}H NMR spectra were recorded immediately after a solution of each is added to preformed [Ir(H)$_3$(COD)(IMes)] in methanol-d_4, and again after 24 hours reaction at room temperature. Note that **4** was preformed by prior reaction of [IrCl(COD)(IMes)] (5 mM) with NaOMe (50 mM) and H_2 (3 bar) in methanol-d_4 for 18 hours at 253 K.

S5.1: ^{1}H NMR spectroscopy of A

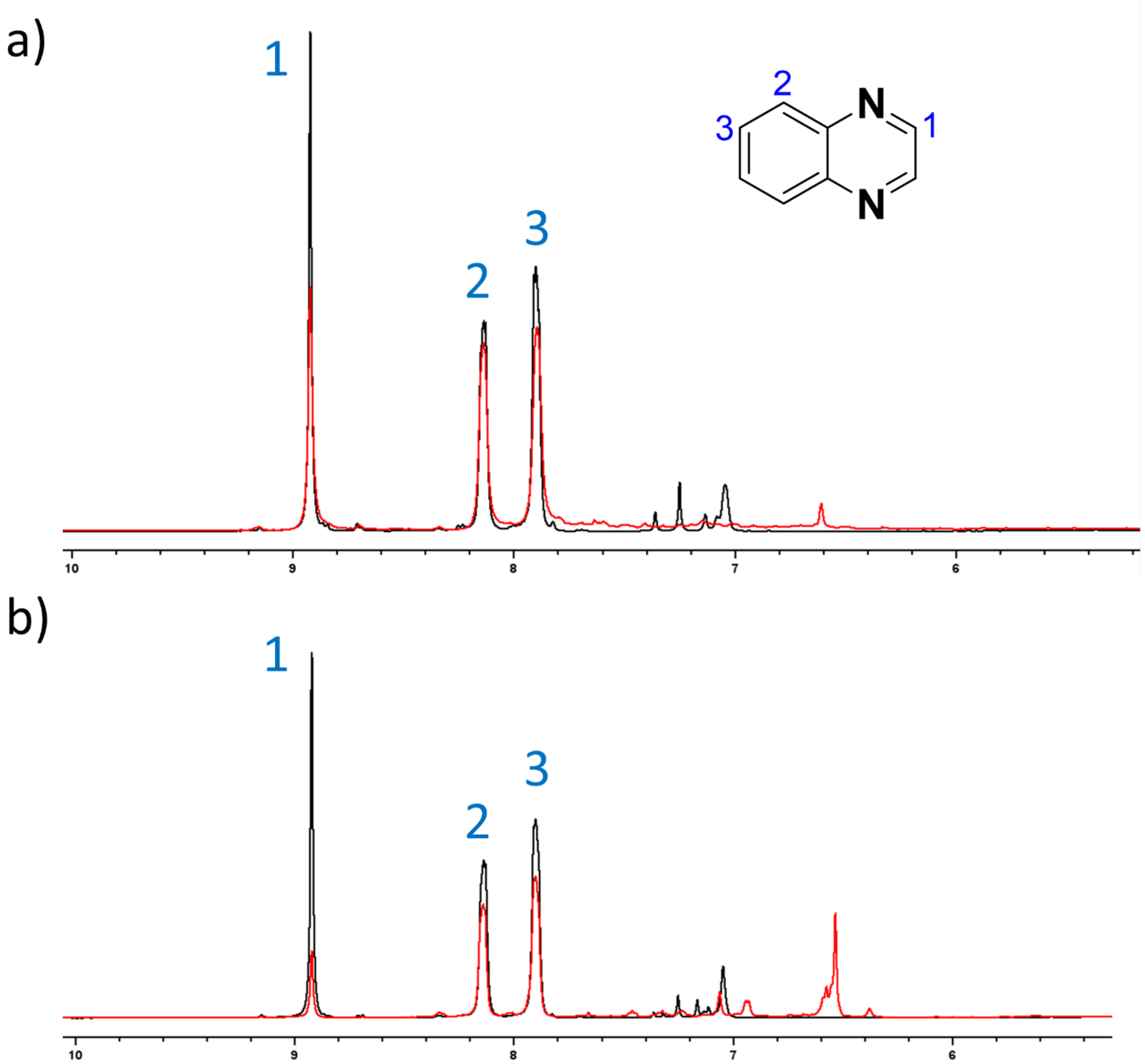

***Figure S23:** Partial ^{1}H NMR spectra of a) A before (black) and after (red) its reaction (50 mM) for 24 hours at room temperature with 1 (5 mM) and H_2 (3 bar) in methanol-d_4 b) A before (black) and after (red) its reaction (50 mM) for 24 hours at room temperature with 1 (5 mM), NaOMe (50 mM) and H_2 (3 bar) in methanol-d_4.*

S5.2: ^{1}H NMR spectroscopy of **B**

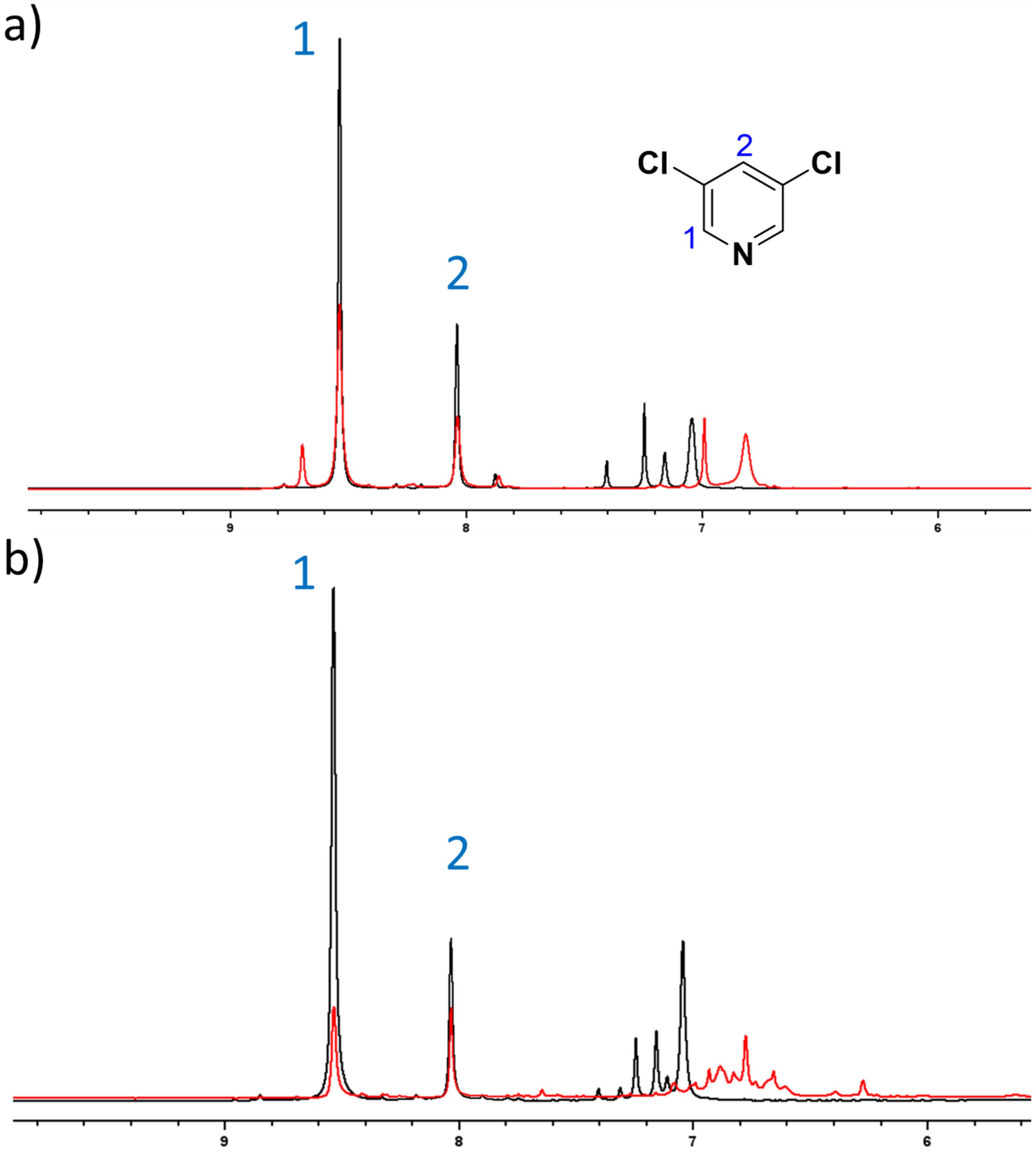

***Figure S24:** Partial ^{1}H NMR spectra of a) B before (black) and after (red) its reaction (50 mM) for 24 hours at room temperature with 1 (5 mM) and H_2 (3 bar) in methanol-d_4 b) B before (black) and after (red) its reaction (50 mM) for 24 hours at room temperature with 1 (5 mM), NaOMe (50 mM) and H_2 (3 bar) in methanol-d_4.*

S5.3: ^{1}H NMR spectroscopy of **C**

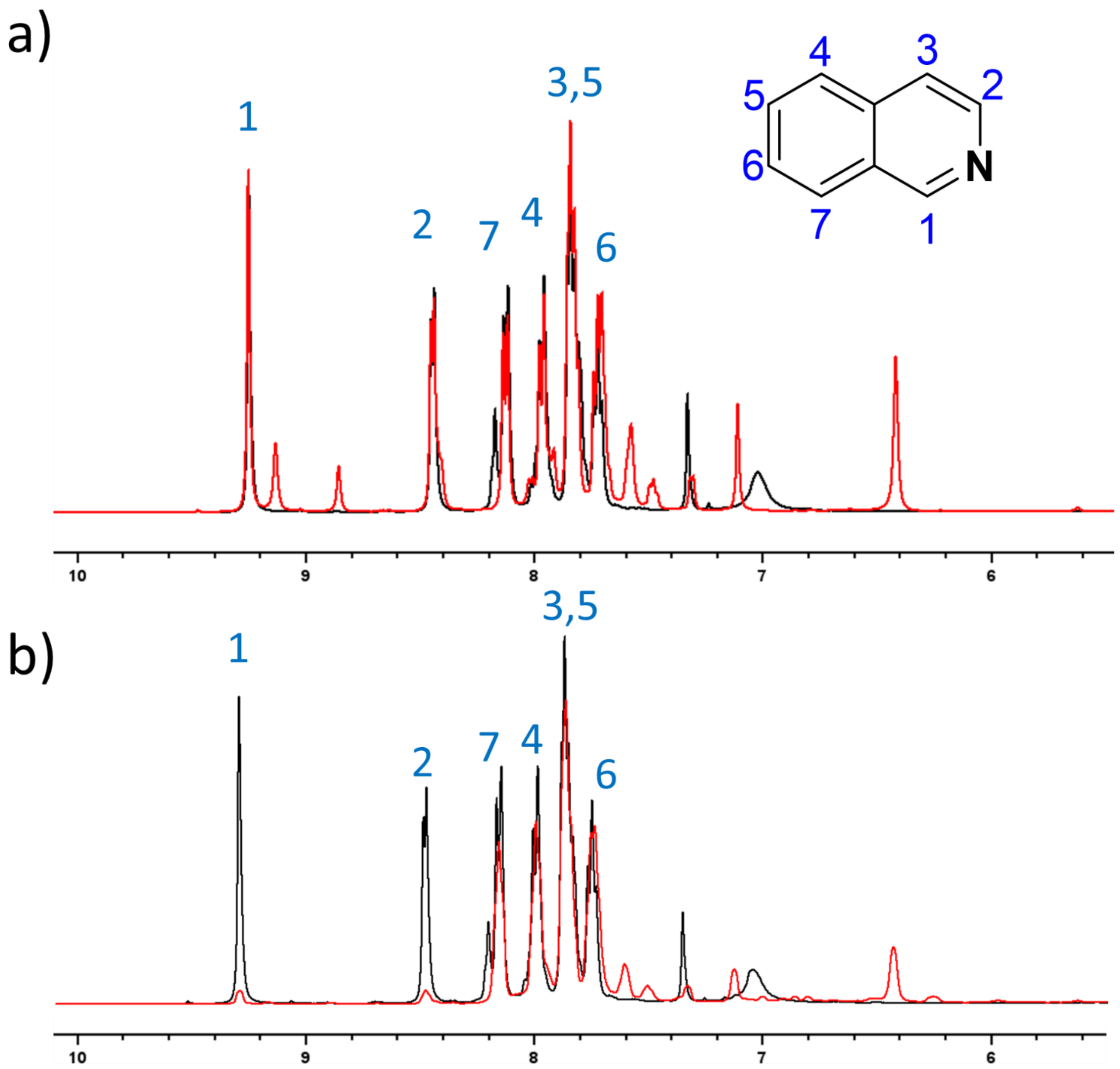

***Figure S25:** Partial ^{1}H NMR spectra of a) C before (black) and after (red) its reaction (50 mM) for 24 hours at room temperature with 1 (5 mM) and H_2 (3 bar) in methanol-d_4 b) C before (black) and after (red) its reaction (50 mM) for 24 hours at room temperature with 1 (5 mM), NaOMe (50 mM) and H_2 (3 bar) in methanol-d_4.*

S5.4: ^{1}H NMR spectroscopy of D

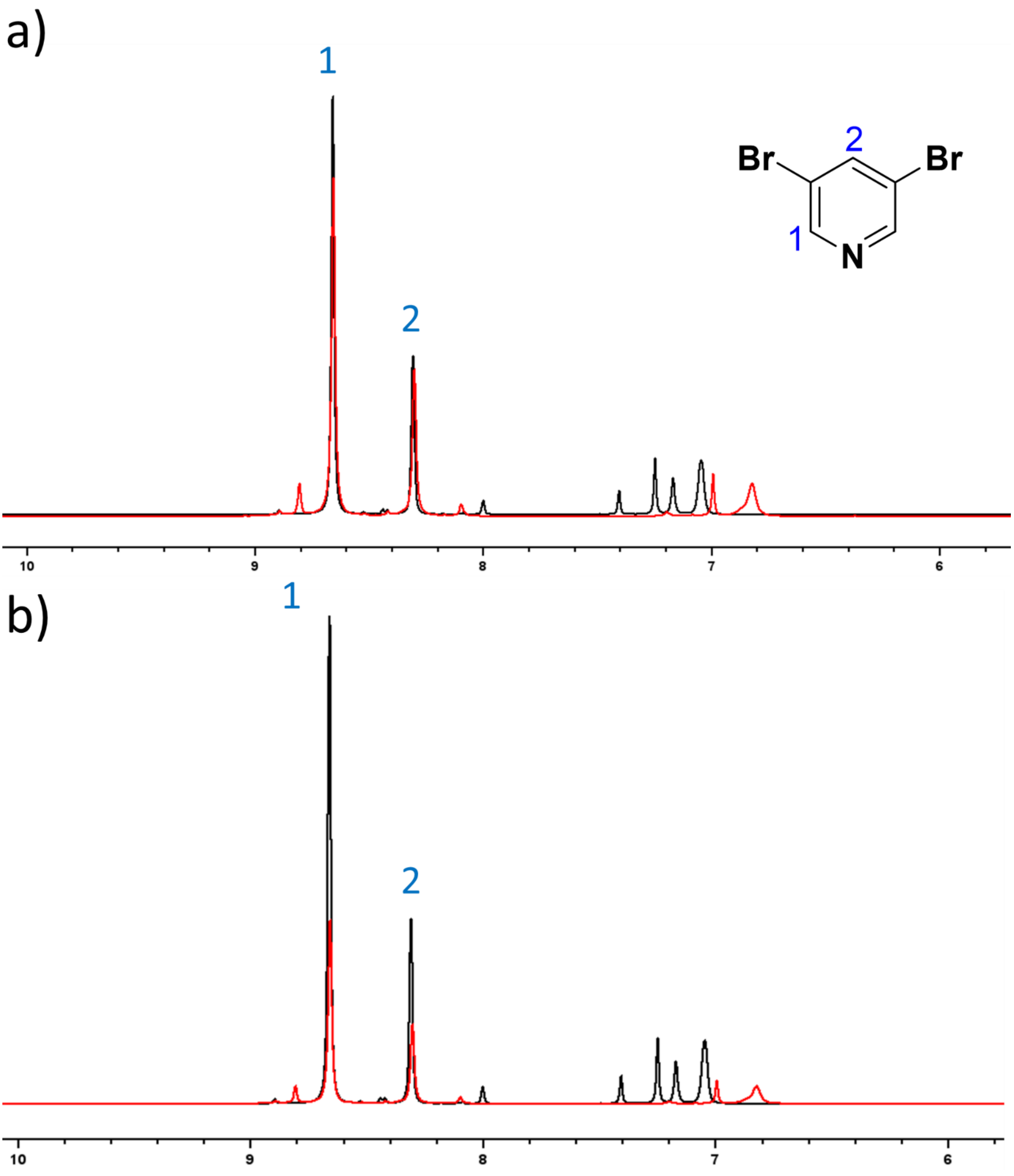

***Figure S26:** Partial ^{1}H NMR spectra of a) D before (black) and after (red) its reaction (50 mM) for 24 hours at room temperature with 1 (5 mM) and H_2 (3 bar) in methanol-d_4 b) D before (black) and after (red) its reaction (50 mM) for 24 hours at room temperature with 1 (5 mM), NaOMe (50 mM) and H_2 (3 bar) in methanol-d_4.*

S5.5: ^1H NMR spectroscopy of **E**

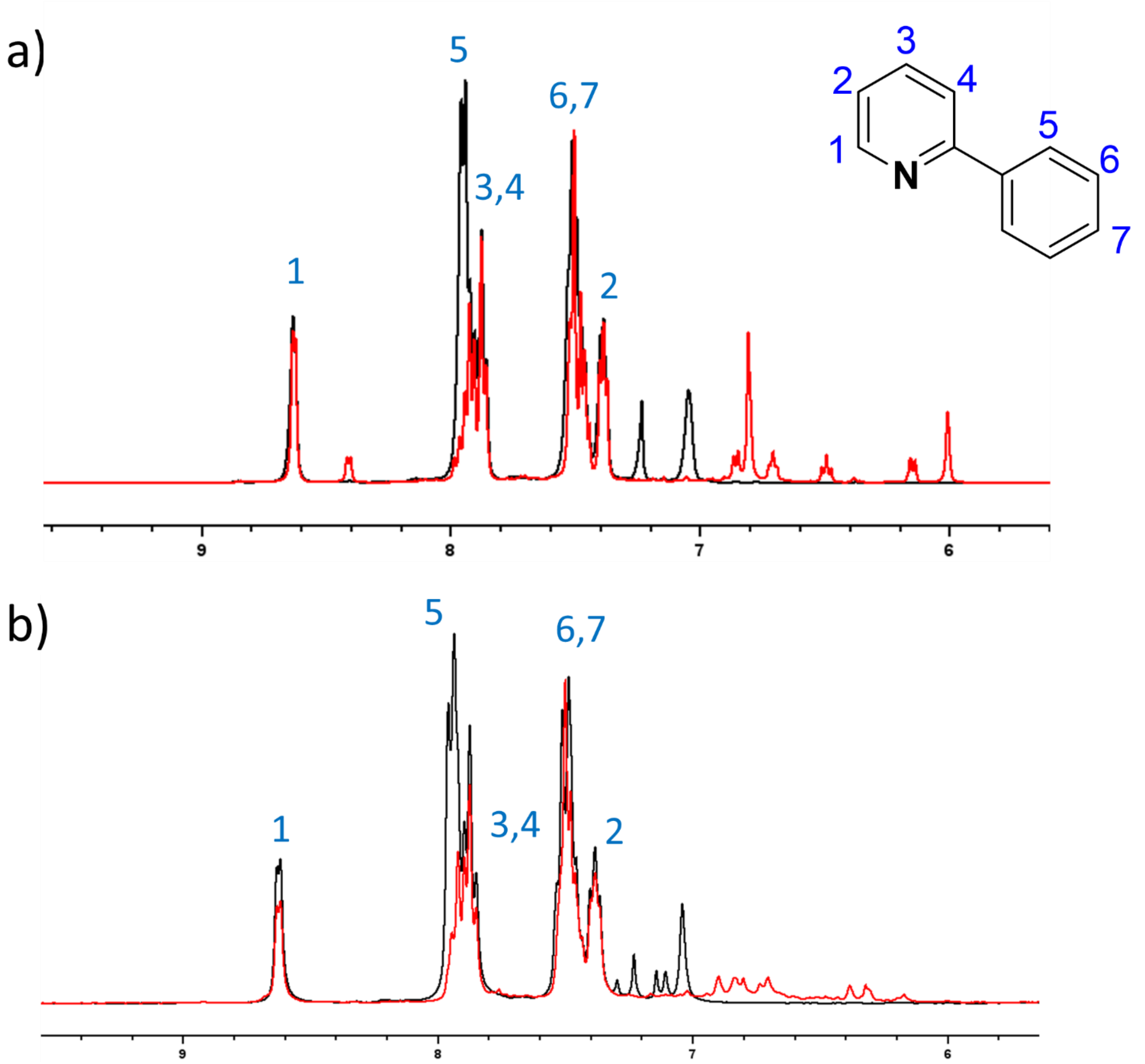

***Figure S27:** Partial ^1H NMR spectra of a) E before (black) and after (red) its reaction (50 mM) for 24 hours at room temperature with 1 (5 mM) and H_2 (3 bar) in methanol-d_4 b) E before (black) and after (red) its reaction (50 mM) for 24 hours at room temperature with 1 (5 mM), NaOMe (50 mM) and H_2 (3 bar) in methanol-d_4.*

S5.6: ^{1}H NMR spectroscopy of **F**

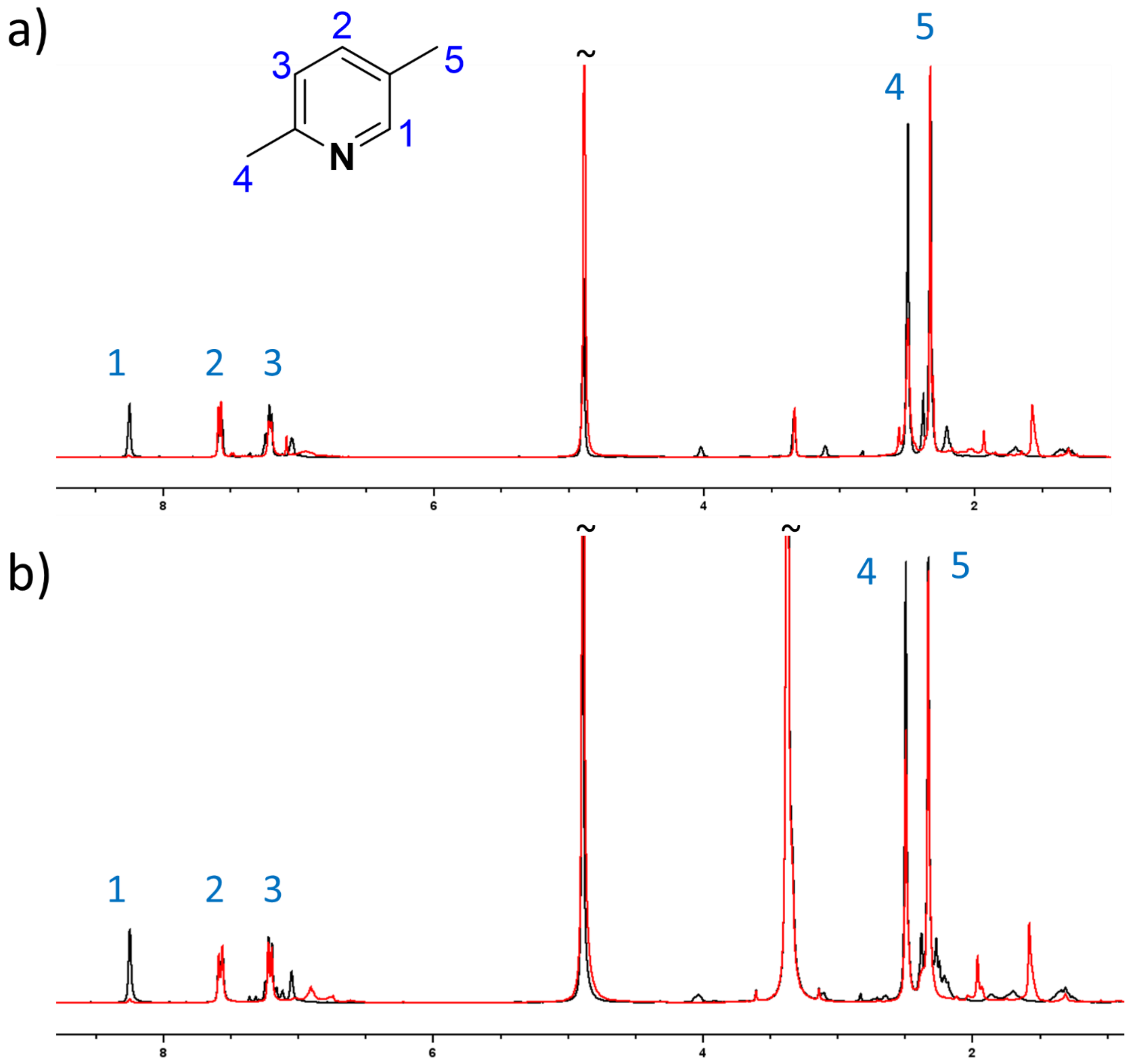

Figure S28: *Partial ^{1}H NMR spectra of a) F before (black) and after (red) its reaction (50 mM) for 24 hours at room temperature with 1 (5 mM) and H_2 (3 bar) in methanol-d_4 b) F before (black) and after (red) its reaction (50 mM) for 24 hours at room temperature with 1 (5 mM), NaOMe (50 mM) and H_2 (3 bar) in methanol-d_4.*

S5.7: ^{1}H NMR spectroscopy of **G**

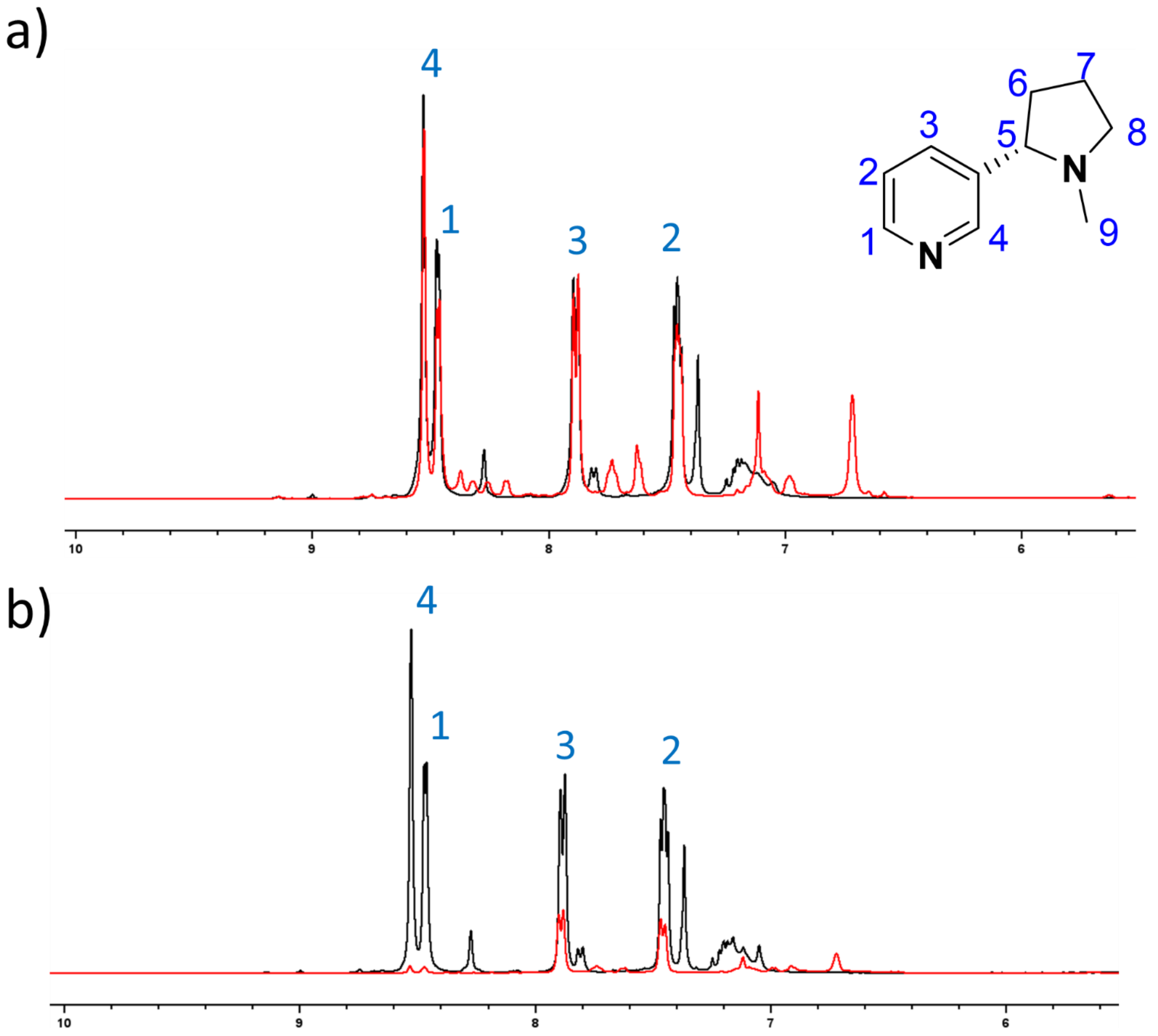

Figure S29: Partial ^{1}H NMR spectra of a) G before (black) and after (red) its reaction (50 mM) for 24 hours at room temperature with 1 (5 mM) and H_2 (3 bar) in methanol-d_4 b) G before (black) and after (red) its reaction (50 mM) for 24 hours at room temperature with 1 (5 mM), NaOMe (50 mM) and H_2 (3 bar) in methanol-d_4.

S5.8: ^{1}H NMR spectroscopy of H

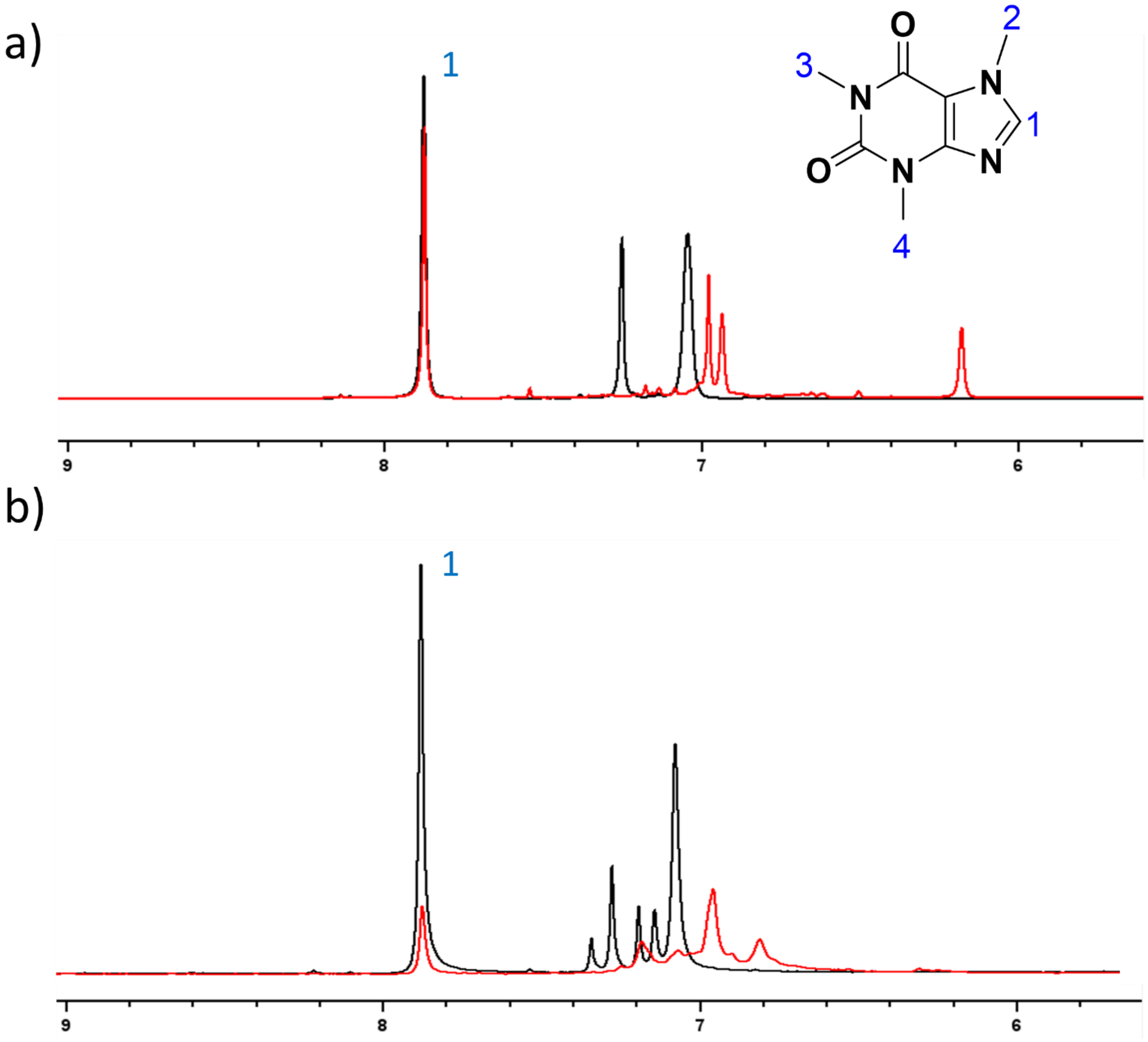

Figure S30: Partial ^{1}H NMR spectra of a) H before (black) and after (red) its reaction (50 mM) for 24 hours at room temperature with 1 (5 mM) and H_2 (3 bar) in methanol-d_4 b) H before (black) and after (red) its reaction (50 mM) for 24 hours at room temperature with 1 (5 mM), NaOMe (50 mM) and H_2 (3 bar) in methanol-d_4.

S5.9: ^{1}H NMR spectroscopy of I

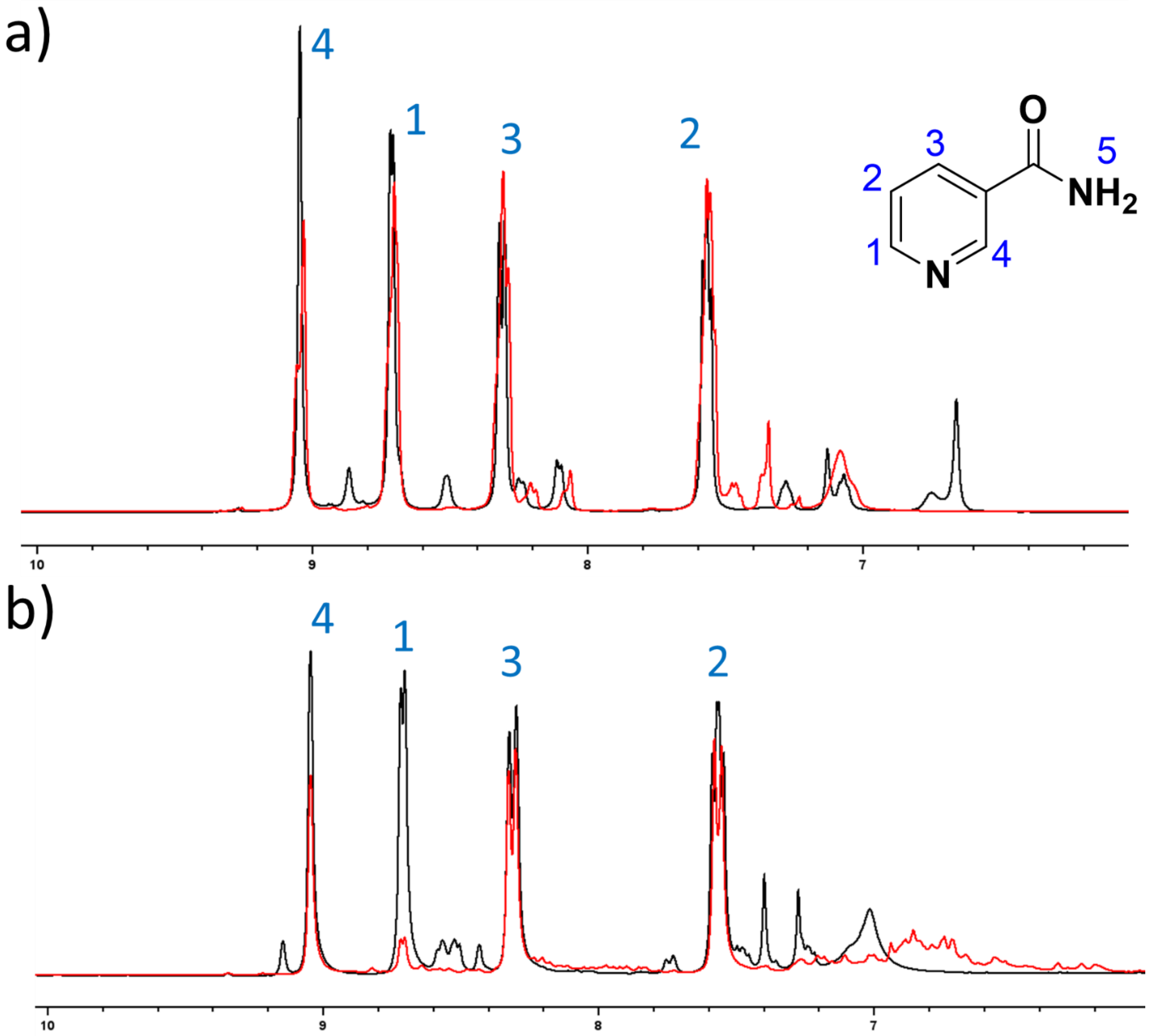

***Figure S31:** Partial ^{1}H NMR spectra of a) I before (black) and after (red) its reaction (50 mM) for 24 hours at room temperature with 1 (5 mM) and H_2 (3 bar) in methanol-d_4 b) I before (black) and after (red) its reaction (50 mM) for 24 hours at room temperature with 1 (5 mM), NaOMe (50 mM) and H_2 (3 bar) in methanol-d_4.*

S5.10: ^{1}H NMR spectroscopy of J

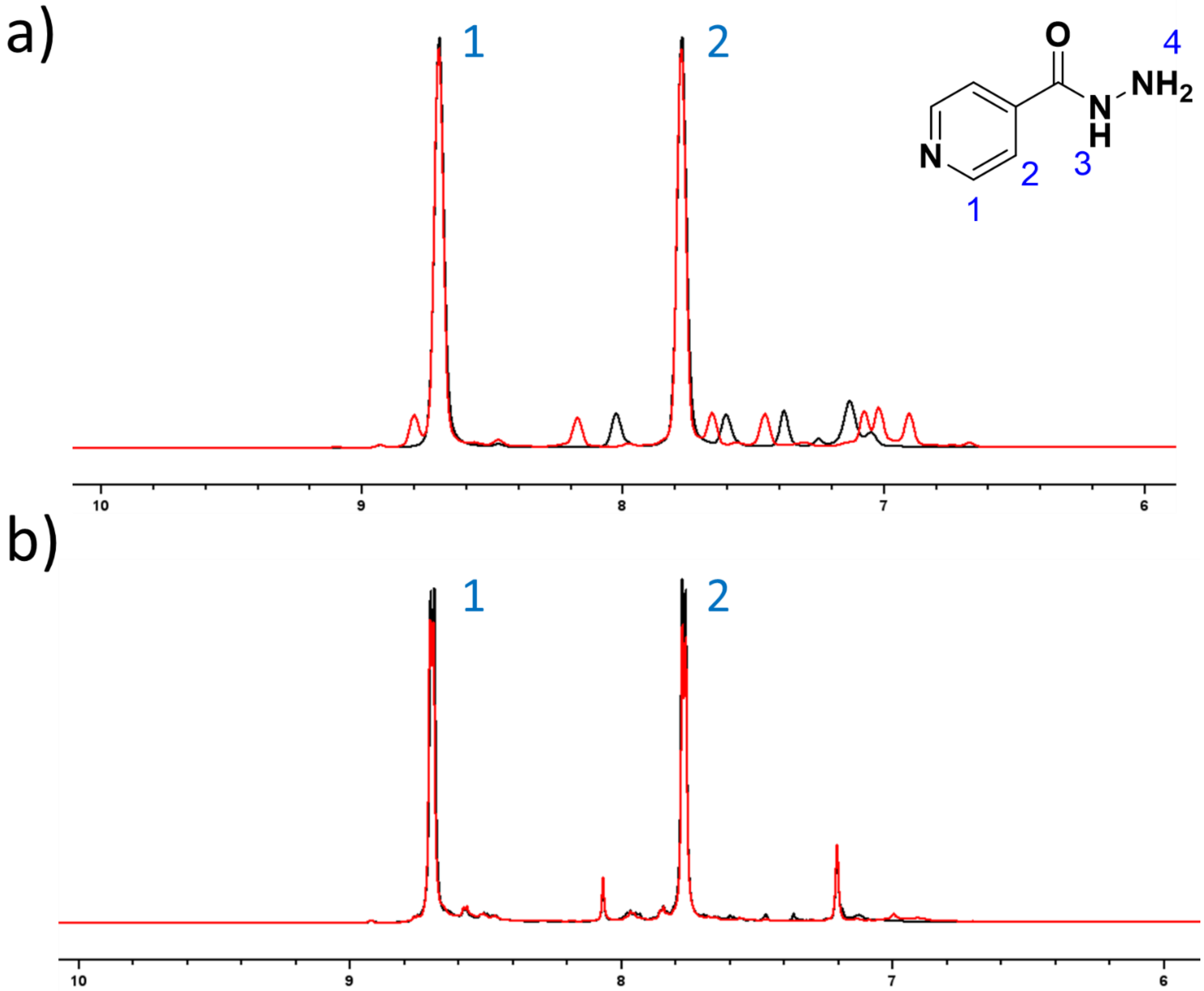

***Figure S32:** Partial ^{1}H NMR spectra of a) J before (black) and after (red) its reaction (50 mM) for 24 hours at room temperature with 1 (5 mM) and H_2 (3 bar) in methanol-d_4 b) J before (black) and after (red) its reaction (50 mM) for 24 hours at room temperature with 1 (5 mM), NaOMe (50 mM) and H_2 (3 bar) in methanol-d_4.*

S5.11: ^{1}H NMR spectroscopy of **K**

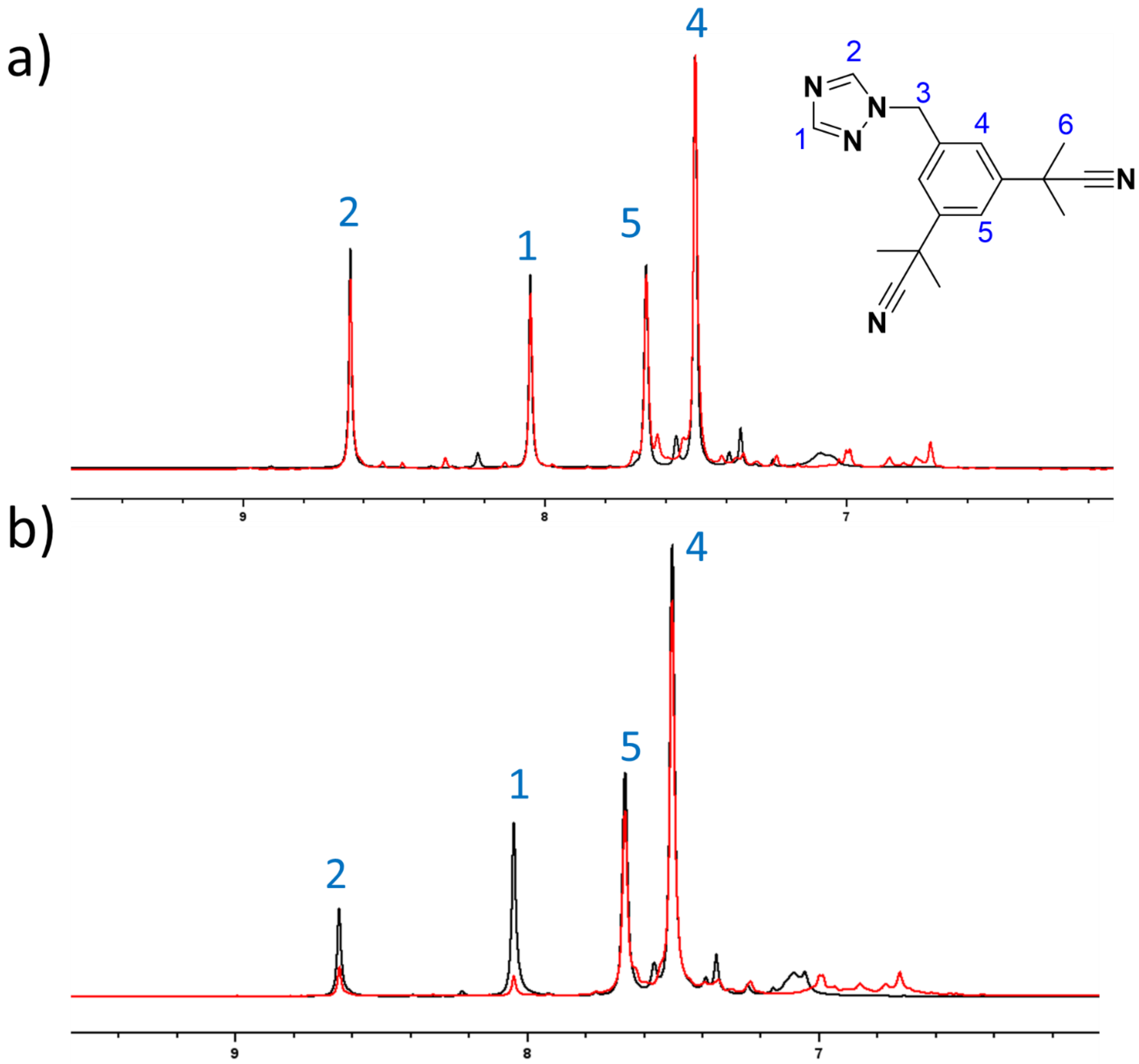

***Figure S33:** Partial ^{1}H NMR spectra of a) K before (black) and after (red) its reaction (50 mM) for 24 hours at room temperature with 1 (5 mM) and H_2 (3 bar) in methanol-d_4 b) K before (black) and after (red) its reaction (50 mM) for 24 hours at room temperature with 1 (5 mM), NaOMe (50 mM) and H_2 (3 bar) in methanol-d_4.*

S5.12: ^{1}H NMR spectroscopy of **L**

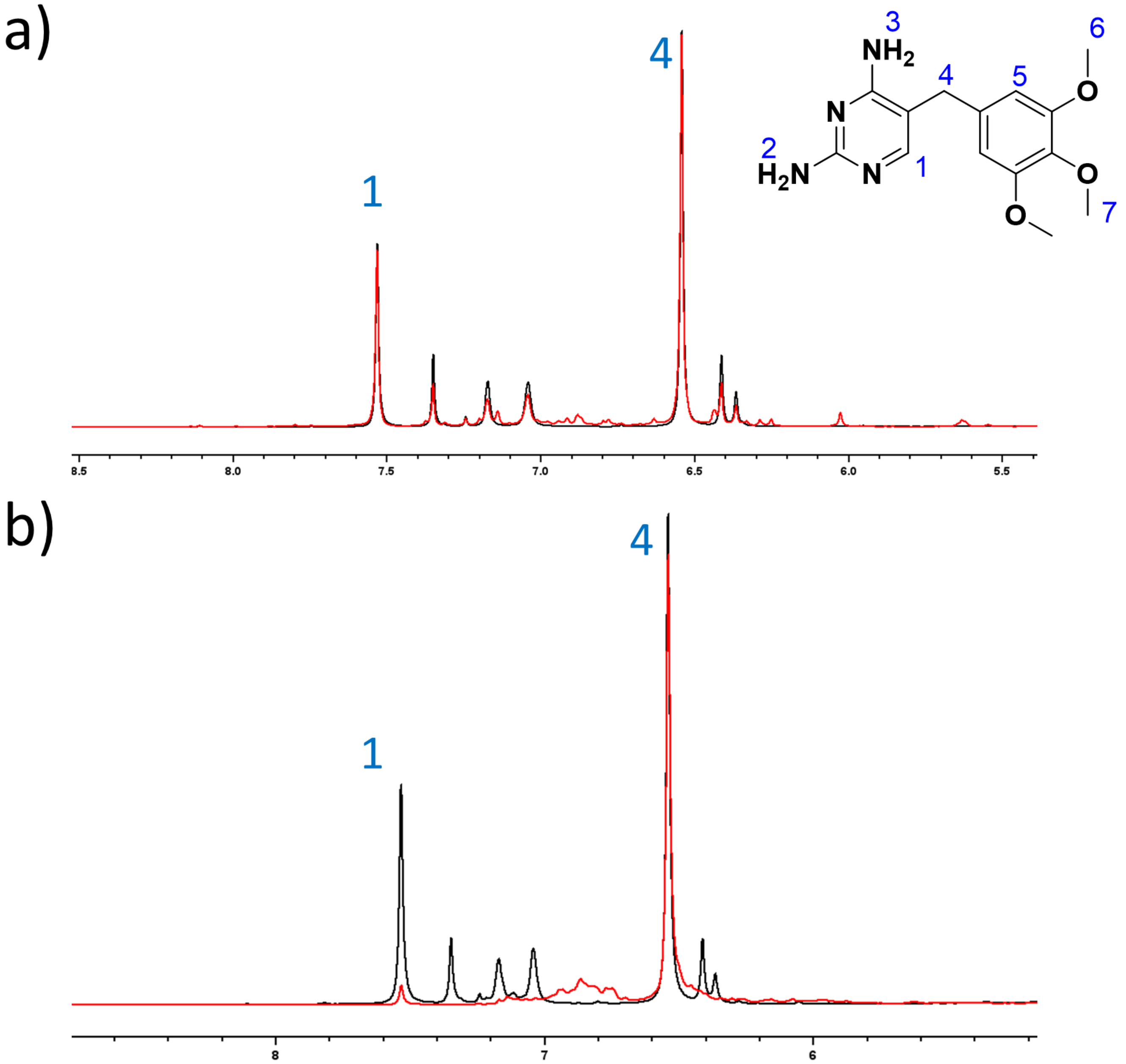

Figure S34: Partial ^{1}H NMR spectra of a) L before (black) and after (red) its reaction (50 mM) for 24 hours at room temperature with 1 (5 mM) and H_2 (3 bar) in methanol-d_4 b) L before (black) and after (red) its reaction (50 mM) for 24 hours at room temperature with 1 (5 mM), NaOMe (50 mM) and H_2 (3 bar) in methanol-d_4.

S5.13: ^{1}H NMR spectroscopy of N

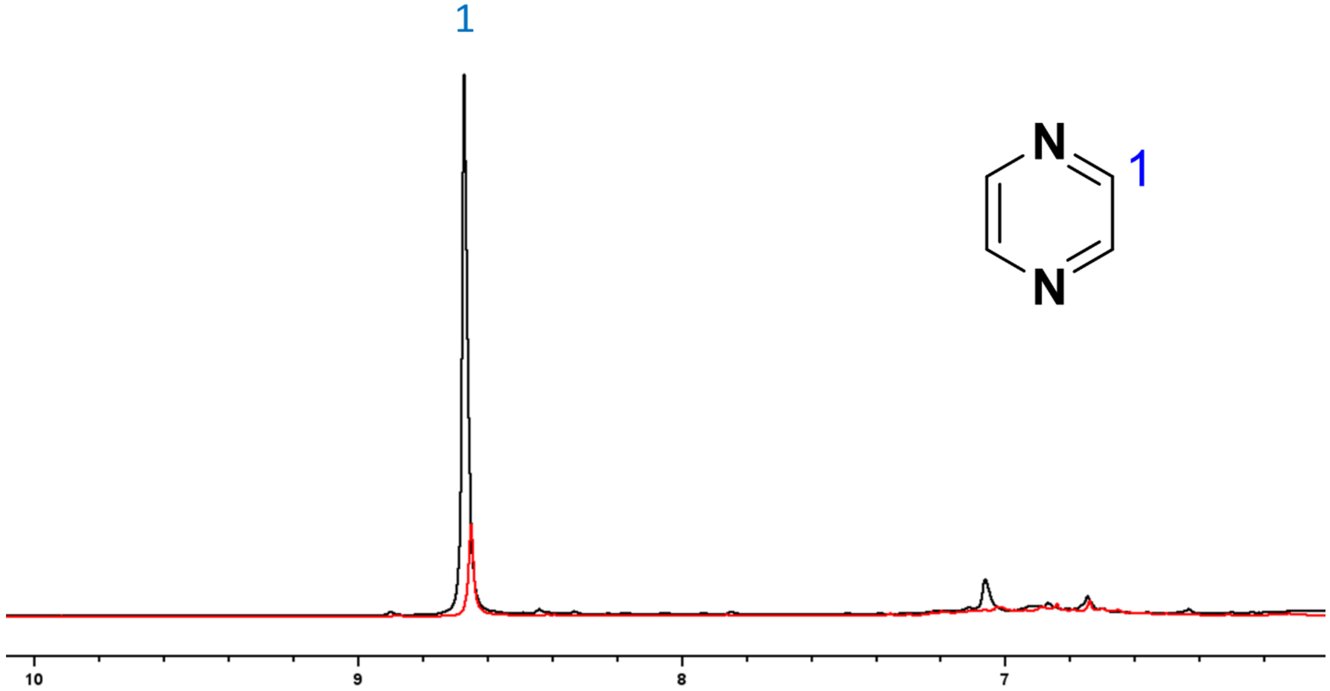

Figure S35: Partial ^{1}H NMR spectra of N immediately after its addition to preformed [Ir(H)$_3$(COD)(IMes)] (black) and after (red) 24 hour reaction in methanol-d$_4$ at room temperature.

S5.14: ^{1}H NMR spectroscopy of O

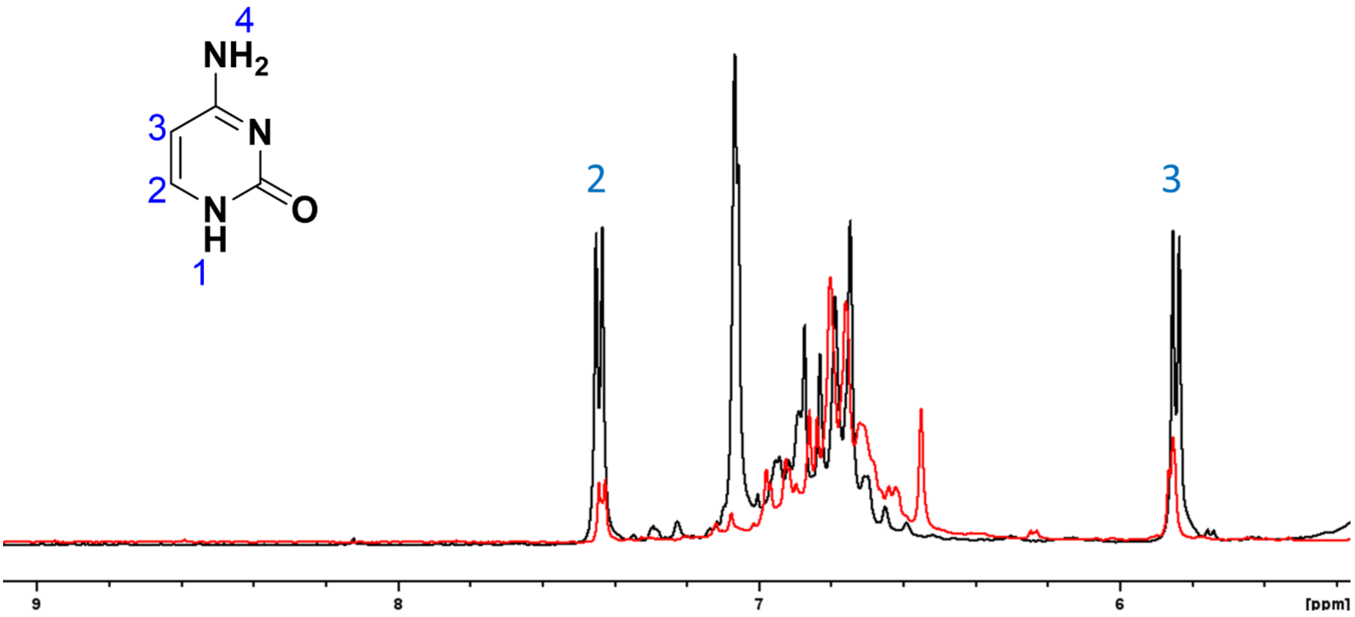

Figure S36: Partial ^{1}H NMR spectra of O immediately after its addition to preformed [Ir(H)$_3$(COD)(IMes)] (black) and after (red) 24 hour reaction in methanol-d$_4$ at room temperature.

S5.15: ^{1}H NMR spectroscopy of P

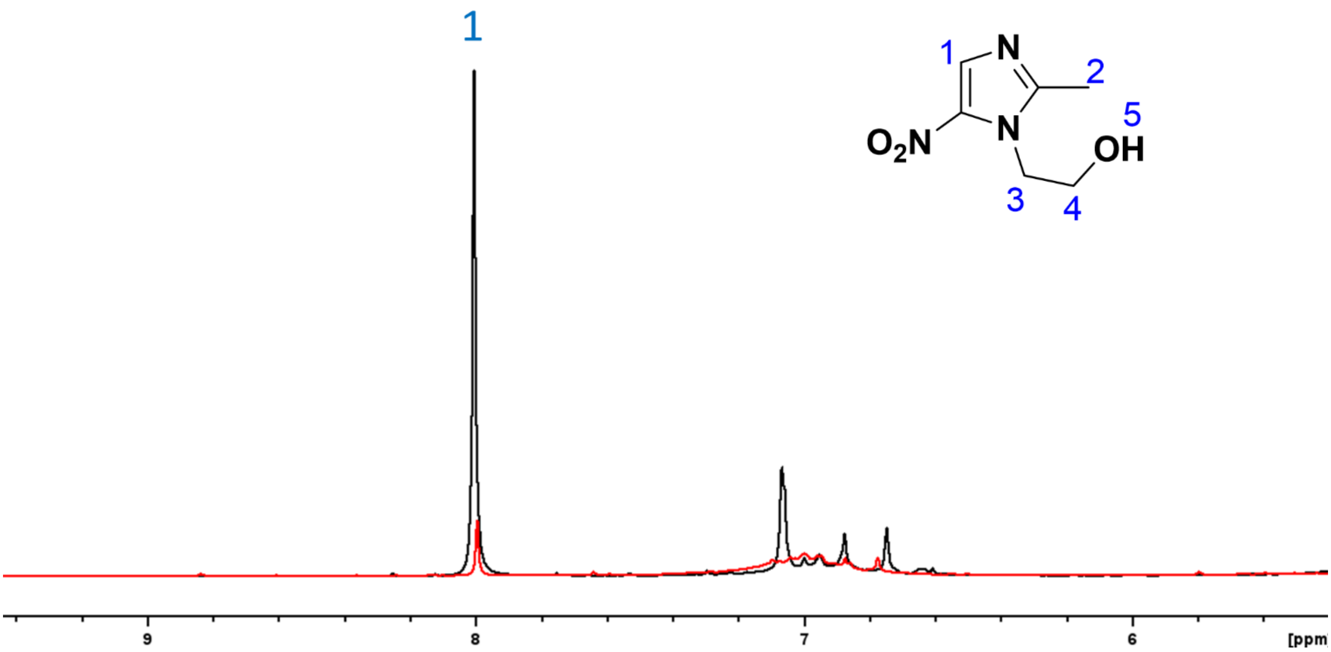

Figure S37: Partial ^{1}H NMR spectra of P immediately after its addition to preformed [Ir(H)$_3$(COD)(IMes)] (black) and after (red) 24 hour reaction in methanol-d$_4$ at room temperature.

S5.16: ^{1}H NMR spectroscopy of Q

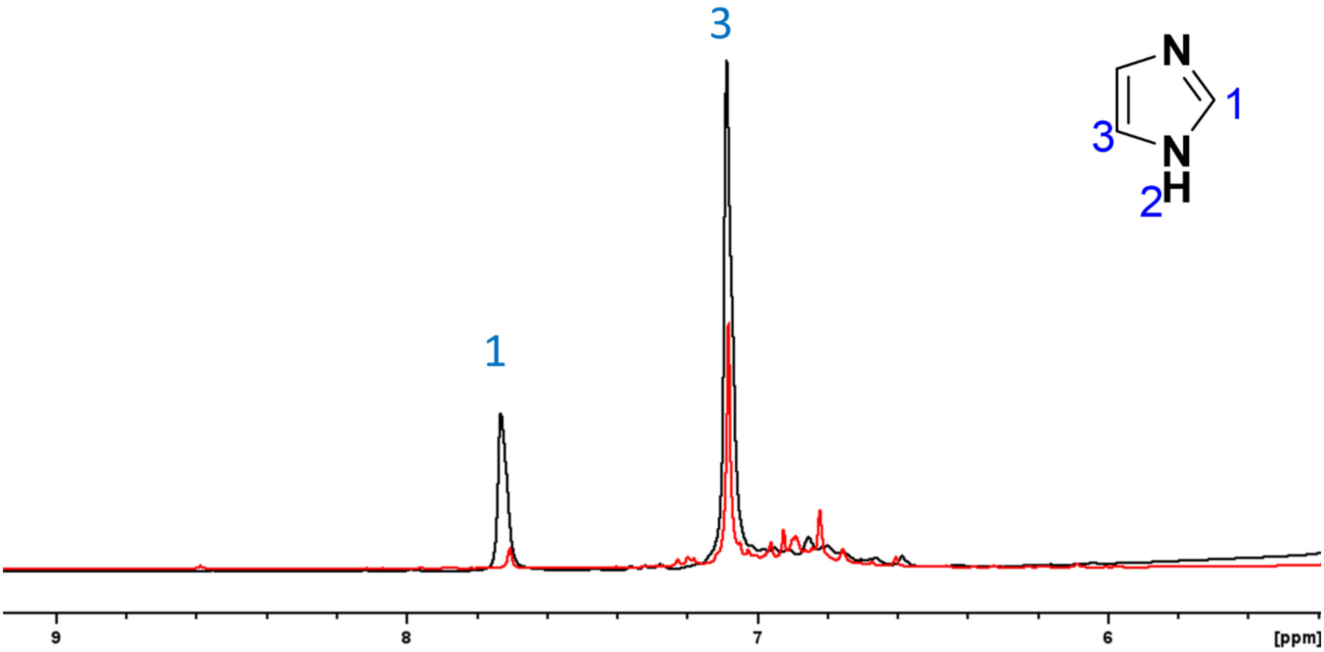

Figure S38: Partial ^{1}H NMR spectra of Q immediately after its addition to preformed [Ir(H)$_3$(COD)(IMes)] (black) and after (red) 24 hour reaction in methanol-d$_4$ at room temperature.

S6: Large Scale Deuteration of Quinoxaline (A)

A (0.201 g, 1.5 mmol), **1** (0.097 g, 0.15 mmol, 10 mol %), NaOMe (0.102 g, 1.9 mmol) were dissolved in methanol-*d*$_4$ (25 mL) under N_2 in an ampoule. The solution was degassed on a Schlenk line H_2 (*ca.* 2.8 bar) was introduced to the ampoule. The reaction mixture was stirred at rt for 4 days. The reaction was monitored by ^{1}H NMR and fresh H_2 (*ca.* 3 bar) was added to the system, and allowed to stir for a further 4 days. The H_2 atmosphere was removed and replaced with air. The mixture was filtered to remove a red precipitate and the filtrate was transferred to a single neck round bottom flask and the solvent was evaporated *in vacuo*. The crude product was dissolved in CH_2Cl_2 (20 mL) and washed with deionized water (3 x 20 mL). The organic layer was dried over $MgSO_4$ and the solvent was evaporated. The crude residue was purified *via* column chromatography (silica, hexane, EtOAc, 1:1 v/v) to give the deuterated product as an orange solid (0.139 g, 66%).

^{1}H NMR analysis revealed the deuteration levels after 8 days were 99 % for H_a, 86 % for H_b and 85 % for H_c (Figure S39).

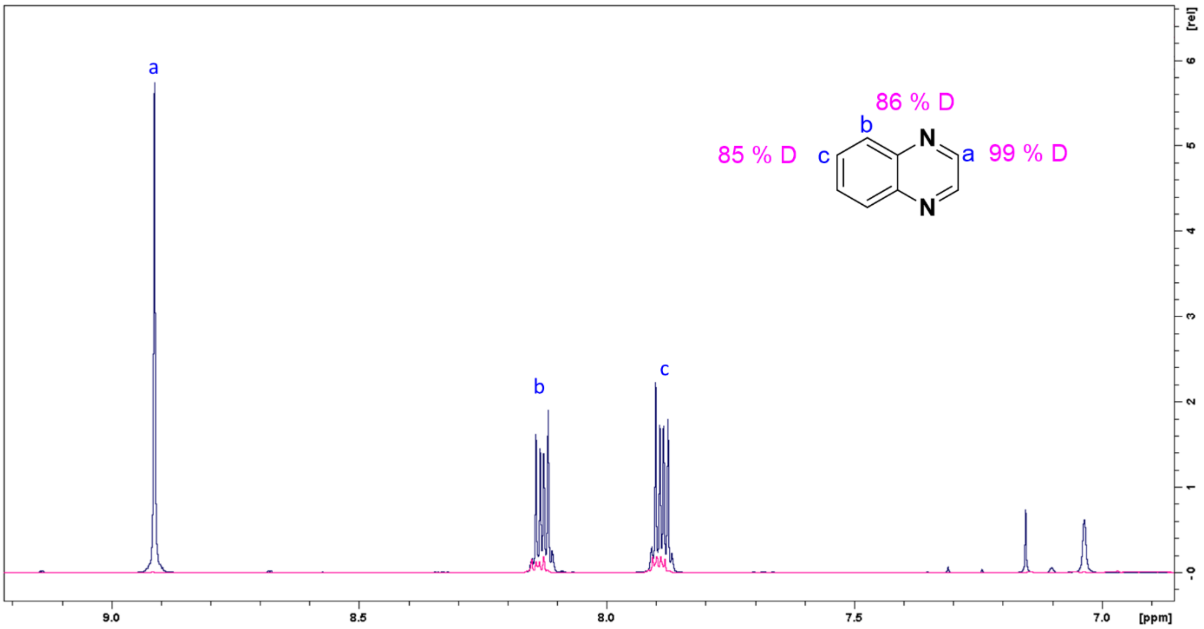

***Figure S39:** Partial ^{1}H NMR spectra of 0.6 mL aliquot of large-scale reaction of A (1.5 mmol), 1 (0.15 mmol) and NaOMe (1.9 mmol) in methanol-d$_4$ (25 mL) before H_2 addition (blue) and after 8 days reaction at room temperature (pink).*

LC-MS analysis (ESI$^+$) of the isolated product revealed multiple deuteration products, with the mass ion for **A-*d*$_6$** showing the highest intensity (Figure S40).

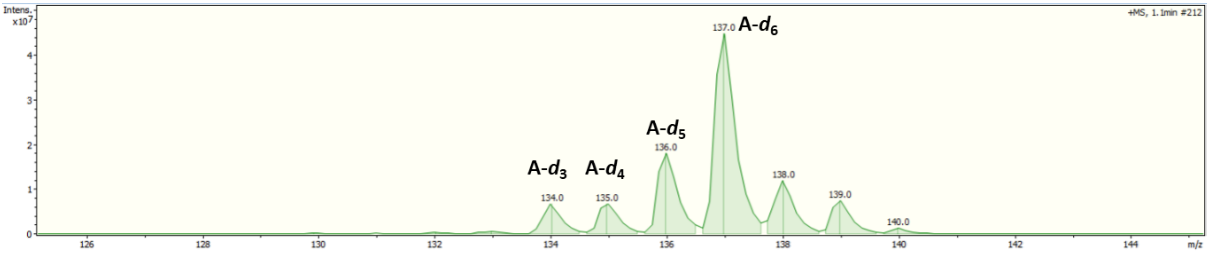

***Figure S40:** Mass spectrum of isolated product from large scale deuteration reaction of A.*

S7: Triplicate Studies on Quinoxaline (A), 3,5-Dichloropyridine (B) and 2-Phenylpyridine (E)

These triplicate experiments were performed under the same conditions per run to establish reproducibility in the level of ^{2}H incorporation. They employed twice the level of base to that used in the substrate scope studies detailed in section 3. These results highlight that both the level of base employed and the reaction time play a role in controlling what is determined to be a reproducible deuteration level. It should be noted that increasing the reaction time consistently increases the deuteration level. In addition, the level of base was found to affect the site deuteration level differently. These studies collectively therefore reveal an impressive level of substrate scope, but it is clear that further optimizations, specific to each substrate, are required to get even higher ^{2}H levels.

1 (2 mg), the indicated substrate (10 equivalents relative to **1**), and NaOMe (25 wt.% in methanol, 20 equivalents relative to **1**) were dissolved in methanol-d_4 (0.6 mL). The solution was degassed via three freeze-pump-thaw cycles using a Schlenk line before filling the tube with H_2 (3 bar). ^{1}H NMR spectra were collected before addition of H_2 and both ^{1}H NMR and were performed after 22-hour reaction at room temperature, and again after 46 hours at room temperature.

Details of the deuteration of substrates in the triplicate study are provided as:
(a) Amount of catalyst
(b) Amount of substrate
(c) Amount of base
(d) Volume of solvent
(e) % D in labelled substrate after 22 h
(f) % D in labelled substrate after 46 h

S7.1: Triplicate Studies of A

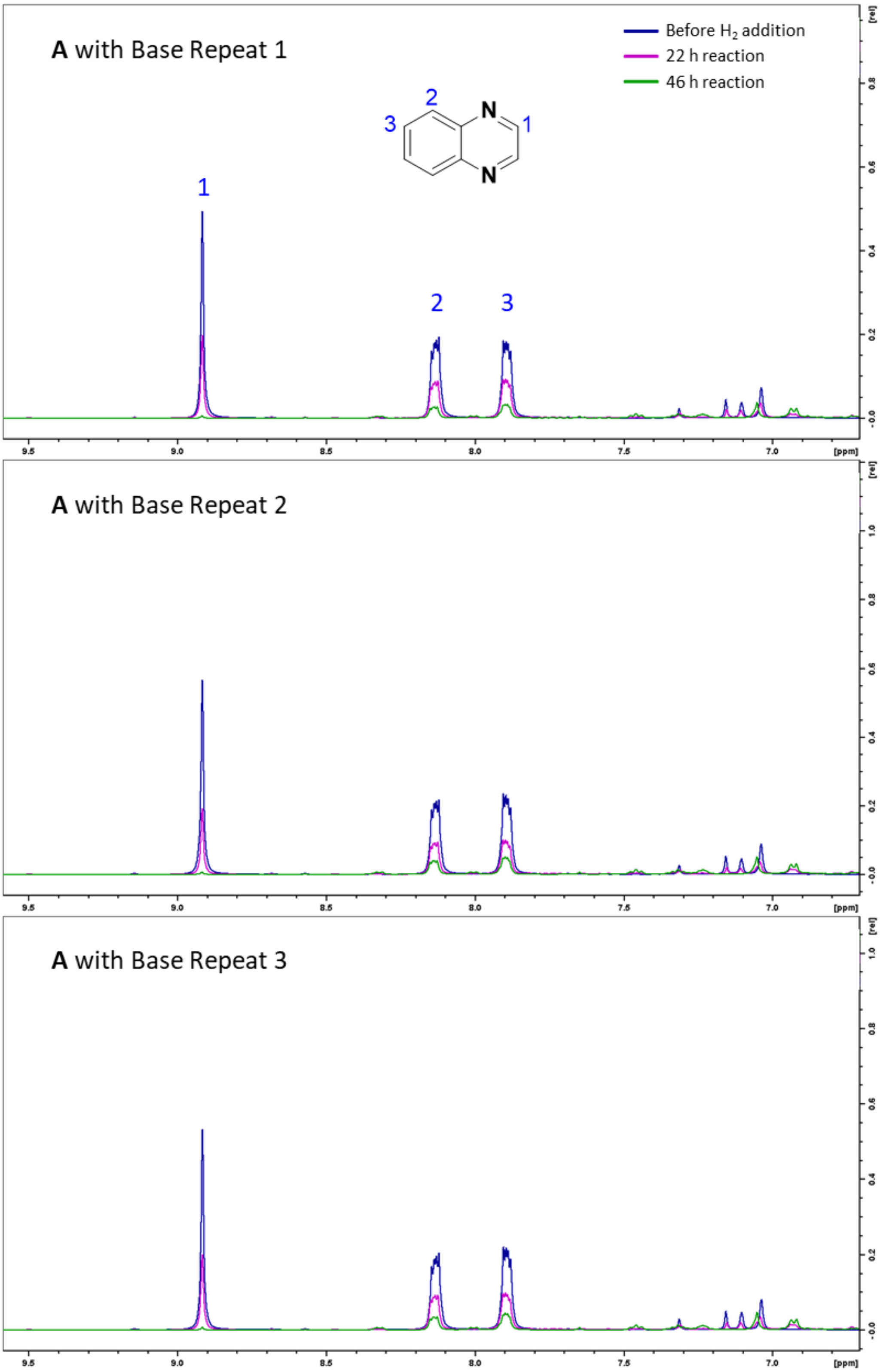

***Figure S41:** Triplicate studies on A. Partial ^{1}H NMR spectra of A before (blue), after 22 h (pink), and after 46 h reaction (50 mM) for room temperature with 1 (5 mM), NaOMe (100 mM) and H_2 (3 bar) in methanol-d_4.*

Repeat 1
(a) **1** (1.90 mg, 0.003 mmol, 9.9 mol %), (b) **A** (3.90 mg, 0.030 mmol), NaOMe (13.7 µL of a 25% w/w solution in MeOH, 0.060 mmol), (d) methanol-d_4 (0.6 mL), (e) H-1 56 % D, H-2 52 % D, H-3 46 % D, (f) H-1 99 % D, H-2 87 % D, H-3 82 % D.

Repeat 2
(a) **1** (1.90 mg, 0.003 mmol, 9.8 mol %), (b) **A** (3.93 mg, 0.030 mmol), NaOMe (13.7 µL of a 25% w/w solution in MeOH, 0.060 mmol), (d) methanol-d_4 (0.6 mL), (e) H-1 65 % D, H-2 56 % D, H-3 53 % D, (f) H-1 98 % D, H-2 80 % D, H-3 77 % D.

Repeat 3
(a) **1** (1.93 mg, 0.003 mmol, 9.9 mol %), (b) **A** (3.97 mg, 0.031 mmol), NaOMe (13.7 µL of a 25% w/w solution in MeOH, 0.060 mmol), (d) methanol-d_4 (0.6 mL), (e) H-1 59 % D, H-2 51 % D, H-3 51 % D, (f) H-1 99 % D, H-2 84 % D, H-3 80 % D.

Table S5: Data for Triplicate Studies of A.

		Deuteration Level (%)			Average Deuteration (%)*
H/D Exchange Site	Reaction Time	Run 1	Run 2	Run 3	
H-1	22 h	56.3	64.5	58.7	59.8 ± 2.4
	46 h	99.3	98.1	99.2	98.9 ± 0.4
H-2	22 h	52.1	55.8	51.4	53.1 ± 1.4
	46 h	86.5	80.3	83.8	83.2 ± 1.8
H-3	22 h	46.0	53.3	50.6	50.0 ± 2.1
	46 h	82.4	77.0	79.2	79.5 ± 1.6

* Errors are given as standard errors of the mean

S7.2: Triplicate Studies of B

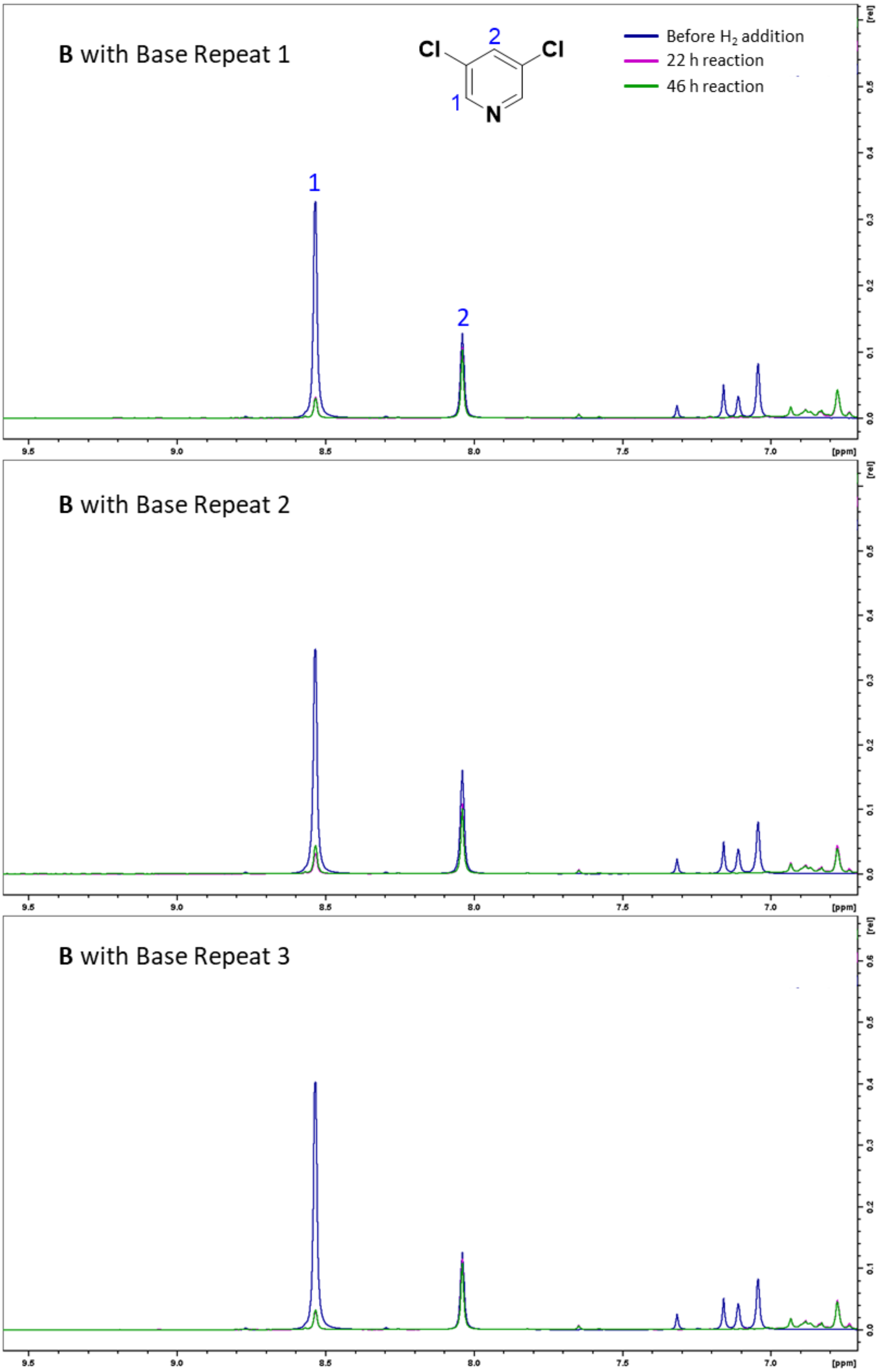

***Figure S42:** Triplicate studies on B. Partial ^{1}H NMR spectra of B before (blue), after 22 h (pink), and after 46 h reaction (50 mM) for room temperature with 1 (5 mM), NaOMe (100 mM) and H_2 (3 bar) in methanol-d_4.*

Repeat 1

(a) **1** (1.92 mg, 0.003 mmol, 10.0 mol %), (b) **B** (4.45 mg, 0.030 mmol), NaOMe (13.7 µL of a 25% w/w solution in MeOH, 0.060 mmol), (d) methanol-d_4 (0.6 mL), (e) H-1 91 % D, H-2 46 % D, (f) H-1 89 % D, H-2 50 % D.

Repeat 2

(a) **1** (1.90 mg, 0.003 mmol, 9.9 mol %), (b) **B** (4.44 mg, 0.030 mmol), NaOMe (13.7 µL of a 25% w/w solution in MeOH, 0.060 mmol), (d) methanol-d_4 (0.6 mL), (e) H-1 92 % D, H-2 27 % D, (f) H-1 92 % D, H-2 30 % D.

Repeat 3

(a) **1** (1.90 mg, 0.003 mmol, 9.9 mol %), (b) **B** (4.44 mg, 0.030 mmol), NaOMe (13.7 µL of a 25% w/w solution in MeOH, 0.060 mmol), (d) methanol-d_4 (0.6 mL), (e) H-1 90 % D, H-2 32 % D, (f) H-1 91 % D, H-2 38 % D.

Table S6: Data for Triplicate Studies of B.

		Deuteration Level (%)			Average Deuteration (%)*
H/D Exchange Site	Reaction Time	Run 1	Run 2	Run 3	
H-1	22 h	90.7	92.1	89.5	90.8 ± 0.7
	46 h	88.8	91.8	91.3	90.7 ± 0.9
H-2	22 h	46.1	27.2	32.2	35.2 ± 5.7
	46 h	49.9	29.7	38.0	39.2 ± 5.9

* Errors are given as standard errors of the mean

S7.3: Triplicate Studies of E

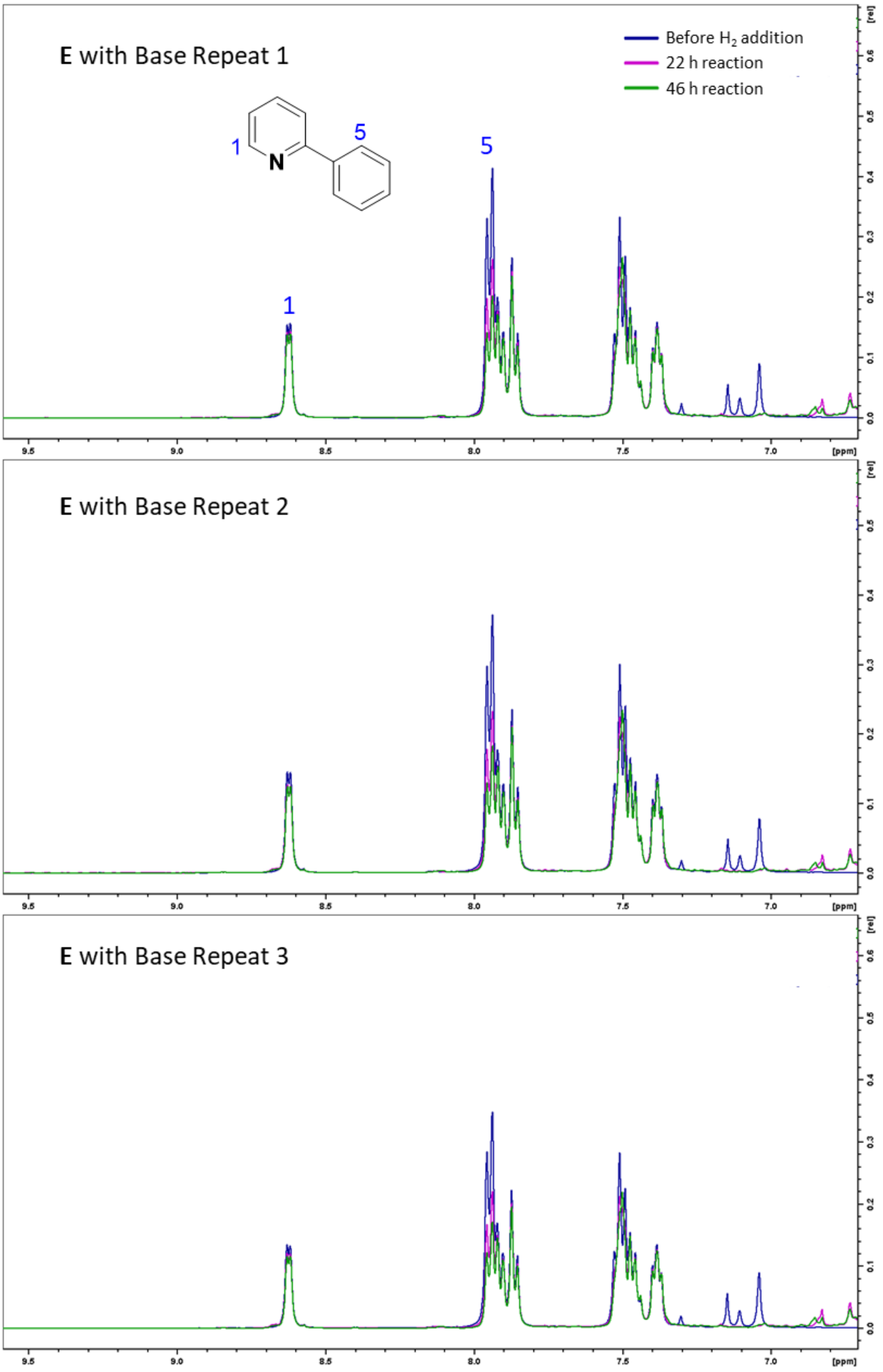

Figure S43: Triplicate studies on E. Partial ^{1}H NMR spectra of E before (blue), after 22 h (pink), and after 46 h reaction (50 mM) for room temperature with 1 (5 mM), NaOMe (100 mM) and H_2 (3 bar) in methanol-d_4.

Repeat 1

(a) **1** (1.87 mg, 0.003 mmol, 9.8 mol %), (b) **E** (4.25 µL, 0.030 mmol), NaOMe (13.7 µL of a 25% w/w solution in MeOH, 0.060 mmol), (d) methanol-d_4 (0.6 mL), (e) H-1 2 % D, H-5 35 % D, (f) H-1 11 % D, H-5 48 % D.

Repeat 2

(a) **1** (1.87 mg, 0.003 mmol, 9.8 mol %), (b) **E** (4.25 µL, 0.030 mmol), NaOMe (13.7 µL of a 25% w/w solution in MeOH, 0.060 mmol), (d) methanol-d_4 (0.6 mL), (e) H-1 11 % D, H-5 35 % D, (f) H-1 18 % D, H-5 49 % D.

Repeat 3

(a) **1** (1.89 mg, 0.003 mmol, 9.9 mol %), (b) **E** (4.26 µL, 0.030 mmol), NaOMe (13.7 µL of a 25% w/w solution in MeOH, 0.060 mmol), (d) methanol-d_4 (0.6 mL), (e) H-1 0 % D, H-5 37 % D, (f) H-15 % D, H-5 49 % D.

Table S7: Data for Triplicate Studies of E.

		Deuteration Level (%)			Average Deuteration (%)*
H/D Exchange Site	Reaction Time	Run 1	Run 2	Run 3	
H-1	22 h	1.9	10.6	0	4.1 ± 3.3
	46 h	10.5	17.7	15.1	14.4 ± 2.1
H-5	22 h	34.5	35.2	37.1	35.6 ± 0.8
	46 h	48.3	49.2	48.5	48.7 ± 0.3

* Errors are given as standard errors of the mean
